# PKMYT1 has an important role in the timing and fidelity of chromosome segregation

**DOI:** 10.1038/s44319-026-00809-1

**Published:** 2026-06-05

**Authors:** Asma Belbelazi, Alexandra Hanna Hendry, Eleanor Wendy Trotter, Charlie Greenaway-Wells, Zoe Edwards, Keren Dawson, Jonathon Pines, Iain Hagan

**Affiliations:** 1 Cell Division, Cancer Research UK Manchester Institute, The University of Manchester, Manchester, Wilmslow Road, M20 4GJ UK; 2 The Institute of Cancer Research, 237 Fulham Road, London, SW3 6JB UK

**Keywords:** Cell Cycle, Signal Transduction

## Abstract

Mitotic entry is a tightly regulated process controlled by CDK1-Cyclin B1. Its activity is negatively regulated by the WEE family kinases, WEE1, WEE2 and PKMYT1, to prevent premature mitotic entry. While WEE1 has an established role in regulating CDK2 during S-phase, the full scope of PKMYT1’s roles in the cell cycle is surprisingly unexplored. Here, we show that PKMYT1 activity increases during anaphase and we reveal a novel mitotic function for PKMYT1 that is distinct from its family member WEE1. Chemical inhibition of PKMYT1 induces premature anaphase, leading to chromosome segregation errors. These errors, including chromatin bridges and micronuclei, consequently activate the cGAS–STING pathway. We further demonstrate that PKMYT1 contributes to maintaining spindle assembly checkpoint integrity, as its inhibition promotes mitotic slippage in the presence of anti-microtubule drugs. Our findings reveal that PKMYT1 provides an additional regulatory mechanism, acting alongside Cyclin B1 degradation, to control CDK1-Cyclin B1 activity and ensure the fidelity of the metaphase-to-anaphase transition.

## Synopsis

Beyond its canonical G2/M role, PKMYT1 is essential for mitotic fidelity. Inhibiting PKMYT1 accelerates mitotic progression and causes chromosome segregation errors, ultimately activating cGAS–STING signalling by altering CDK1-Cyclin B1 regulation at anaphase onset.

- PKMYT1 activity increases during metaphase–anaphase transition, having a role distinct from its canonical G2/M function and its family member WEE1.
- PKMYT1 inhibition accelerates metaphase–anaphase transition, triggering chromosome segregation errors and subsequent cGAS–STING pathway activation.
- PKMYT1 regulates CDK1–Cyclin B1 activity during metaphase–anaphase transition to ensure precise timing of mitotic exit.
- PKMYT1 contributes to the maintenance of spindle assembly checkpoint (SAC) robustness during microtubule stress.

## Abstract (web summary)

Beyond its canonical G2/M role, PKMYT1 is essential for mitotic fidelity. Inhibiting PKMYT1 accelerates mitotic progression and causes chromosome segregation errors, ultimately activating cGAS–STING signalling by altering CDK1-Cyclin B1 regulation at anaphase onset.

## Graphical abstract

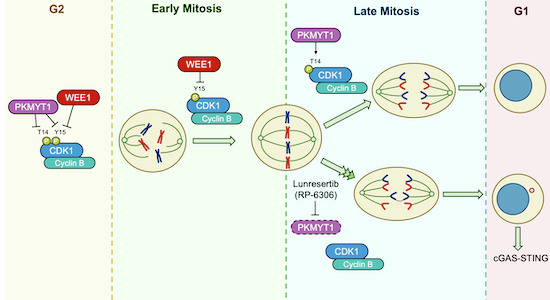

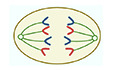

## Introduction

Cell division is a highly regulated process that ensures the faithful duplication of the genome and its equal distribution to two daughter cells. A critical step, the commitment to mitosis, is governed by the activity of the master kinase, cyclin-dependent kinase 1 (CDK1)-Cyclin B1 (Lee and Nurse, 1987; Evans et al, 1983; Dorée and Hunt, 2002). This activity is negatively controlled by the WEE family kinases that include WEE1, WEE2, and PKMYT1. These kinases act as crucial gatekeepers of the G2/M checkpoint, phosphorylating and inactivating CDK1 to serve as a surveillance mechanism that safeguards genomic stability (Thuriaux et al, 1978; Mueller et al, 1995; Ciccia and Elledge 2010; Schmidt et al, 2017).

While WEE1 is a well-characterized nuclear kinase that phosphorylates CDK1 and CDK2 on tyrosine 15 (Y15), and is predominantly active in somatic cells, its family member WEE2 is thought to play a more specific role in regulating the early embryonic cell cycle (Parker and Piwnica-Worms, 1992; Nakanishi et al, 2000). In contrast, significantly less is known about their functional counterpart, PKMYT1. PKMYT1 is a dual-specificity kinase that phosphorylates CDK1 on both Y15 and threonine 14 (T14). Although WEE1 is known to be highly regulated during mitosis and contributes to the spindle assembly checkpoint (SAC) strength, the precise role of PKMYT1 in regulating proper mitotic progression, particularly its function during chromosome segregation, remains largely unexplored. (Mueller et al, 1995; Liu et al, 1997; Visconti et al, 2015; Lianga et al, 2013; Watanabe et al, 2004). The known and predicted binding partners of PKMYT1—including Polo-like kinase (Plk), checkpoint kinase 1 (Chk1), protein phosphatase 1 (PP1), and CDK1—strongly indicate a more diverse functional role for PKMYT1 in mitosis (Aiba et al, 2022; Heroes et al, 2013; Nakajima et al, 2003; Joo et al, 2025). This warrants a comprehensive investigation into its complete role in mitotic regulation.

The prometaphase stage of mitosis, where chromosomes attach to microtubules, is a critical step for accurate chromosome segregation and is tightly governed by SAC. The SAC functions as a surveillance mechanism, delaying the onset of anaphase by inhibiting the anaphase-promoting complex/cyclosome (APC/C) until all kinetochores have achieved proper, bipolar attachment to the mitotic spindle (Fang et al, 1998; Hwang et al, 1998; Clute and Pines, 1999; Peters, 2002; Izawa and Pines, 2015).

The inhibitory SAC signal originates from the Mitotic Checkpoint Complex (MCC), which directly inhibits the APC/C. MCC assembly is initiated at unattached kinetochores through a molecular cascade that requires the recruitment of the kinase Mps1 to the outer kinetochore component KNL1, alongside MAD1-MAD2 complexes. This SAC signal requires the continuous presence of unattached kinetochores (Yamagishi et al, 2012; Shepperd et al, 2012; McAinsh and Kops, 2023). This persistence of unattached kinetochores is mediated by the chromosomal passenger complex (CPC), which acts as the primary error correction system. Localized to the inner centromere, the CPC’s catalytic component, Aurora B kinase, functions as a tension sensor. Aurora B detects and promotes the turnover of faulty, low-tension microtubule attachments, thereby forcing misattached kinetochores back into an unattached state until stable bipolar tension is achieved. This crucial error remediation mechanism ensures the continuous input required for MCC formation (Tanaka et al, 2002; Nezi and Musacchio, 2009; Musacchio and Salmon, 2007; Santaguida et al, 2011). Following the satisfaction of the SAC and the initiation of anaphase, the CPC rapidly relocates to the spindle midzone and then to the midbody (Fuller et al, 2008; Cooke et al, 1987; Vader et al, 2006).

The core mitotic kinase, CDK1-Cyclin B1, actively coordinates the SAC mechanism. Its presence is required at unattached kinetochores to establish robust SAC signaling (Jackman et al, 2020; Hayward et al, 2019; Allan et al, 2020). Furthermore, CDK1-Cyclin B1 directly regulates key components of both the SAC and the CPC through phosphorylation. This regulatory function highlights the kinase’s central role in coordinating the entire mitotic network and ensuring high-fidelity chromosome segregation (D’Angiolella et al, 2003; Yamaguchi et al, 2003; Vanoosthuyse and Hardwick, 2003; Goto et al, 2006).

Here, we provide direct evidence of PKMYT1 activity during mitosis. Our data indicate that PKMYT1’s activity increases as cells approach anaphase. Using a combination of biochemical analysis, cell synchronization, and live-cell imaging, we found that inhibiting mitotic PKMYT1 at metaphase is sufficient to induce a premature anaphase onset, which leads to chromosome segregation errors such as chromatin bridges and micronuclei. In turn, these errors activate the c-GAS-STING signaling pathway.

Furthermore, our results indicate that PKMYT1 contributes to SAC robustness, as its inhibition during a prometaphase arrest enhances mitotic slippage. This work uncovers a novel role for PKMYT1 as a critical regulator of the metaphase-to-anaphase transition.

## Results and discussion

### Initial observations suggest a mitotic role for PKMYT1

To investigate whether PKMYT1’s function extends beyond regulating the initiation of mitosis, we knocked out PKMYT1 in the hTERT-immortalized retinal pigment epithelial (RPE1) cell line. These are diploid cells with a stable karyotype that have all their cell cycle checkpoints intact, and are thus a good model to study cell cycle control. Three different PKMYT1-knockout clones showed a significant increase in micronucleation compared to the wild-type cells (Appendix Fig. S1A,B), indicating a role for PKMYT1 in maintaining genomic stability. Characterization of these clones revealed a marked reduction in CDK1 T14 phosphorylation, whereas Y15 phosphorylation remained comparable to WT levels (Appendix Fig. S1C). This confirms that PKMYT1 is the predominant kinase for T14 in this context.

To investigate the kinetics of this process, we synchronized cells at the G_2_/M transition using sequential CDK4/6 (Palbociclib) and CDK1 (RO-3306) inhibition (Appendix Fig. S2A). Following washout of RO-3306, treatment with the PKMYT1 inhibitor RP 6306 significantly accelerated the rate of mitotic progression relative to DMSO controls (Appendix Fig. S2B–D). Notably, this accelerated phenotype was absent in PKMYT1 −/− cells, where RP 6306 failed to further accelerate the timing of mitotic progression. These data indicate that the observed acceleration is a specific consequence of PKMYT1 inhibition and not an off-target effect of the inhibitor (Appendix Fig. S2E–G).

To further validate this mitotic acceleration at the single-cell level, we performed live-cell imaging of unsynchronized cells progressing through mitosis. To decouple this phenotype from any potential G_2_-phase regulation, we only tracked cells that were in prometaphase at the time of inhibitor addition. In WT cells, PKMYT1 inhibition significantly shortened the duration from prometaphase to anaphase onset compared to DMSO-treated controls (Appendix Fig. S3A,B). Consistent with our previous observations, this acceleration was not observed in PKMYT1−/− cells, confirming that PKMYT1 specifically regulates the kinetics of mitotic progression and that the observed effects were not due to off-target activity (Appendix Fig. S3A,B). Collectively, these findings establish PKMYT1 as a regulator of mitotic timing, independent of its role at the G_2_/M transition.

### Mitotic PKMYT1 activity increases during metaphase

**Figure 1:**
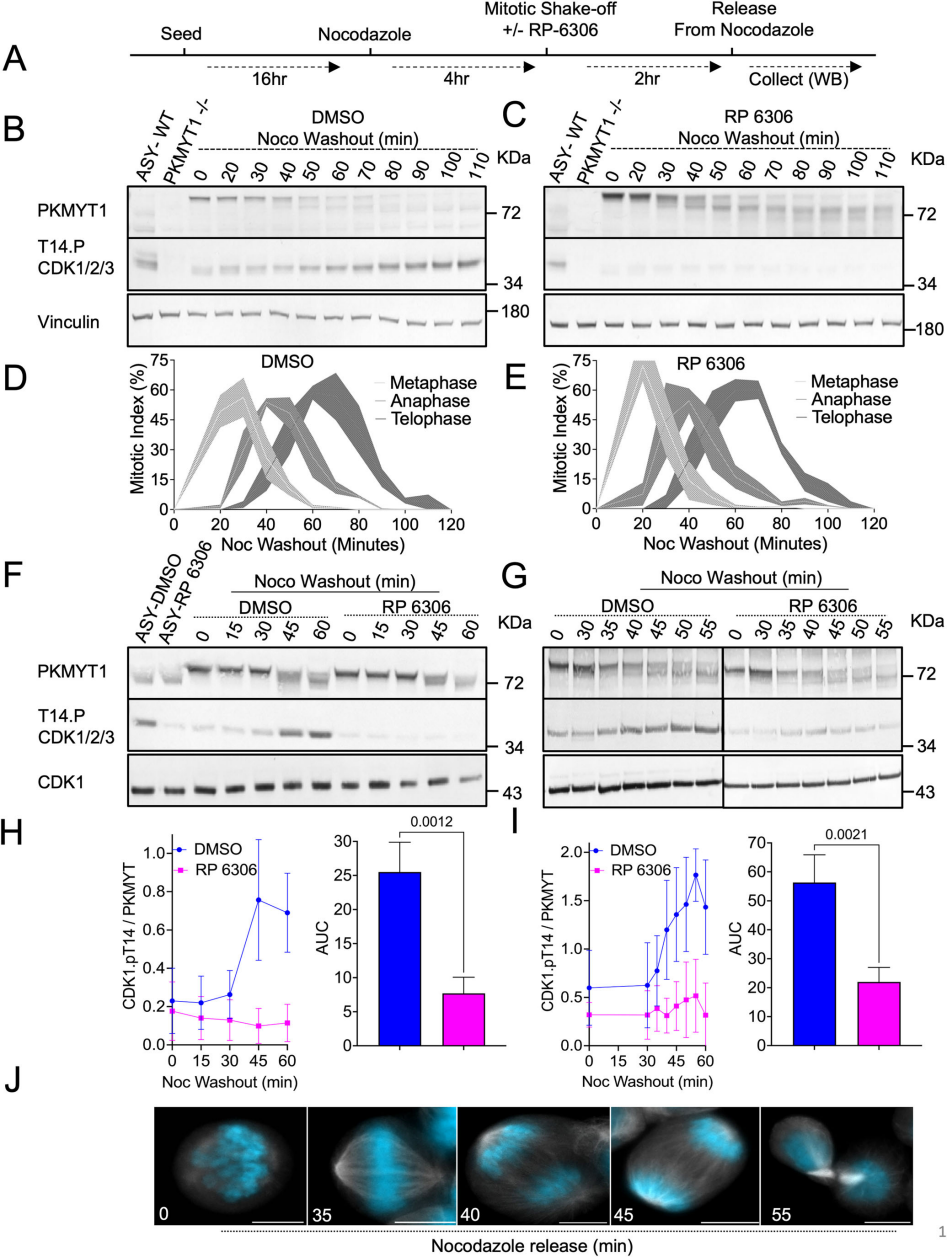
Mitotic PKMYT1 activity increases during the metaphase-to-anaphase transition. (**A**) Outline of the experimental workflow for nocodazole shake-off synchronization of RPE1 cells. (**B**,** C**) Nocodazole-arrested RPE1 cells were maintained in DMEM supplemented with 10% fetal bovine serum. RP 6306 (500 nM) or DMSO were added to the nocodazole-arrested cells 2 h prior to their release. Cells were subsequently released from the nocodazole block using fresh warm media containing either DMSO or RP 6306. Cell lysates were collected every 10 min. “ASY” indicates an asynchronous cell culture control. Equal amounts of protein lysate, derived from a single master mix, were resolved by 12% SDS-PAGE. PKMYT1 and p-T14-CDK1 were detected on the same membrane, with Vinculin run on a parallel membrane as a sample processing control. (**D**, **E**) The progression of RPE1 cells through mitosis was quantified by visual classification, after release from a nocodazole into fresh media containing either DMSO or RP 6306. Each line represents the mean ± SEM, *n* = 3 biological replicates, with at least 100 cells counted at each time point. (**F**,** G**) In vitro kinase assays were performed using PKMYT1 immunoprecipitated from mitotic RPE1 cells. These cells were collected at 15-min intervals following a nocodazole release for (**F**), or at 5-min intervals after the initial 30 min post-release for (**G**). The reaction, containing purified PKMYT1, recombinant CDK1-Cyclin B1, and ATP, was incubated with either DMSO or RP 6306 for 5 min before being halted. “ASY” indicates an asynchronous cell culture control. Samples were resolved by 12% SDS-PAGE and immunoblotted for PKMYT1 and p-T14-CDK1. Total CDK1 was detected on a parallel membrane as a sample processing and loading control. (**H**,** I**) Quantification of P-T14-CDK1 phosphorylation relative to PKMYT1 levels from the in vitro kinase assays shown in (**F**,** G**). Data were presented as mean ± SD, *n* = 3 biological replicates. Statistical analysis was performed on the area under the curve (AUC) by Student’s *t*-test. (**J**) Representative fluorescence confocal images of RPE1 cells post-nocodazole release, showing progression through mitosis. Cells were stained with DAPI (to visualize DNA) and Tat1 (an antibody for α-tubulin, to visualize the mitotic spindle). Scale bar, 5 µm. Source data are available online for this figure.

To examine PKMYT1 activity in mitosis, we isolated nocodazole-arrested cells by mitotic shake-off and released them into fresh media containing either DMSO or a PKMYT1 inhibitor (RP 6306) (Fig. 1A). We observed a dynamic regulation of PKMYT1 phosphorylation during mitosis. Western blot analysis showed that the protein is highly phosphorylated in prometaphase and metaphase, followed by a stepwise dephosphorylation as cells progress into anaphase. This coincided with an increase in T14 phosphorylation on CDK1 in the DMSO-treated cells, indicating that the dephosphorylation of PKMYT1 activates its kinase activity (Fig. 1B,D). Conversely, cells treated with the PKMYT1 inhibitor exhibited a clear reduction in the T14 phosphorylation signal (Fig. 1C,E).

**Figure EV1:**
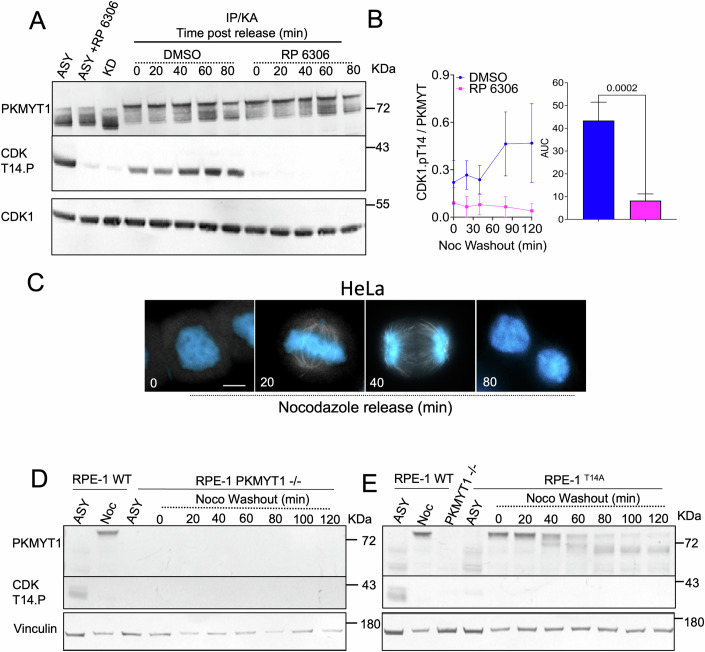
Validation of PKMYT1 Kinase Activity and CDK1 Phosphorylation Kinetics in Mitotic Exit. (**A**) In vitro kinase assay was performed using PKMYT1 immunoprecipitated from mitotic HeLa cell. Lysates were collected every 20 min following release from a nocodazole shake-off. The reaction was conducted as described in Fig. 1**F**,** G**. The samples were analyzed by 12% SDS-PAGE and immunoblotted for PKMYT1, P-T14-CDK1. Total CDK1 was detected on a parallel membrane as a sample processing and loading control. “ASY” indicates an asynchronous cell culture control and “KD” indicates Kinase-Dead purified PKMYT1. (**B**) Quantification of P-T14-CDK1 phosphorylation relative to PKMYT1 levels from the in vitro kinase assays shown in (**A**). Data were presented as mean ± SD, *n* = 3 biological replicates. Statistical analysis was performed using the area under the curve (AUC) by a Student’s *t*-test. (**C**) Representative confocal microscopy images of HeLa cells fixed post-nocodazole release, showing progression through mitosis. Scale bar, 5 μm. (**D**,** E**) Analysis of mitotic lysates from two cell lines with altered PKMYT1 activity: RPE1 PKMYT1 knockout (−/−) and RPE1 CDK1^T14A^. Cells were arrested with nocodazole and then released into fresh media. Cell lysates were collected every 10 min post-release, separated by 12% SDS-PAGE, and immunoblotted for PKMYT1, phospho-threonine 14 CDK1 (P-T14-CDK1), with Vinculin run on a parallel membrane as a sample processing control. Data from the PKMYT1 −/− cell line are shown in (**D**), and data from the CDK1 ^T14A^ cell line are shown in (**E**).

To determine whether mitotic PKMYT1 can directly phosphorylate CDK1, we performed an in vitro kinase assay. We isolated 3xFLAG-PKMYT1 from RPE-FRT cells that had been arrested with nocodazole, then released into fresh media, and cell lysates were collected every 15 min post-release. Co-incubation of the isolated PKMYT1 with recombinant CDK1-Cyclin B1 for 5 min resulted in a noticeable CDK1 phospho-T14 signal, which increased between 30 and 45 min post-release (Fig. 1F,H). This result demonstrates that mitotic PKMYT1 can directly phosphorylate CDK1 at the T14 site. As expected, this phosphorylation was significantly reduced when the PKMYT1 inhibitor was added to the kinase assay reaction. We noted that the increase in the CDK1 phospho-T14 signal between 30 and 45 min coincided with the beginning of PKMYT1 dephosphorylation. This led us to conduct a higher temporal resolution time-course, isolating PKMYT1 every 5 min from 30 to 55 min post-release. We observed the same pattern: dephosphorylation of PKMYT1 alongside an increase in the CDK1 phospho-T14 signal (Fig. 1G,I). Immunofluorescence (IF) images showed that cells began to segregate their chromosomes during this time (Fig. 1J). A similar pattern of PKMYT1 dephosphorylation and an increase in p-CDK1 T14 signal was also observed in HeLa cells (Fig. EV1A–C). The specificity of our signals was confirmed by examining the PKMYT1 knockout and CDK1 ^T14A^ RPE1 cell lines during mitosis. As expected, the PKMYT1 signal was absent in the knockout cells, while the p-CDK1 T14 signal was absent in the CDK1 ^T14A^ cells (Fig. EV1D,E).

Our results indicate that mitotic PKMYT1 is highly phosphorylated and minimally active during early mitosis. It subsequently undergoes stepwise dephosphorylation and reactivation as cells approach anaphase onset, a transition that coincides with increased T14 phosphorylation on CDK1-Cyclin B1. The timing of this reactivation appears essential; recent work demonstrated that Chk1 phosphorylates and inhibits PKMYT1 during prometaphase and metaphase to ensure chromosome segregation fidelity (Joo et al, 2025). This elegant regulatory cascade ensures that while Chk1 suppresses PKMYT1 at earlier stages to permit sustained CDK1-Cyclin B1 activity, the subsequent activation of PKMYT1 is required for precise CDK1 regulation at the onset of anaphase and for coordinating later mitotic events.

### Inhibiting mitotic PKMYT1 Accelerates anaphase onset

**Figure 2:**
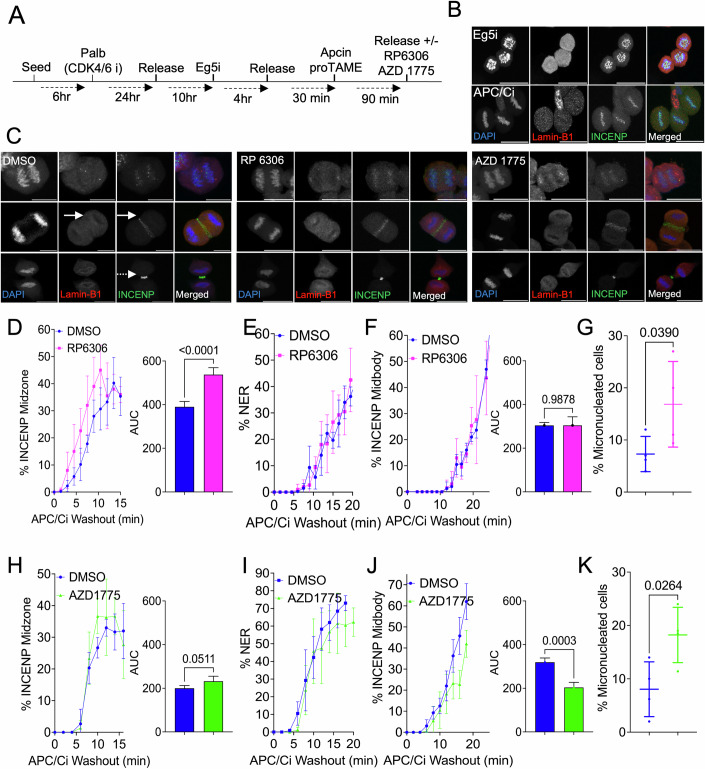
Inhibiting Mitotic PKMYT1 Accelerates Anaphase Onset. (**A**) Outline of the experimental workflow for metaphase synchronization of RPE1 cells using an Eg5 inhibitor and Apcin/ProTAME. (**B**) Representative maximum intensity projections of fluorescence confocal images of RPE1 cells arrested with either an Eg5i or Apcin/ProTAME. Cells were stained with DAPI (DNA, blue), INCENP (green), and Lamin B1 (red). Scale bar, 5 µm. (**C**) Representative maximum intensity projections of RPE1 cells released from metaphase arrest into fresh warm media containing either DMSO, RP 6306, or AZD 1775. Cells were fixed and stained with DAPI (DNA, blue), INCENP (green), and Lamin B1 (red). Scale bar, 5 µm. The solid arrow shows the localization of INCENP at the midzone and NER, and the dotted arrow shows the localization of INCENP to the midbody. (**D**–**G**) Quantification of mitotic progression in cells released into RP 6306. (**D**) The percentage of cells with midzone localization of INCENP. (**E**) The percentage of cells that had begun NER. (**F**) The percentage of cells with midbody localization of INCENP. (**G**) Rate of micronucleation at 3 h post-release. (**H**–**K**) Quantification of mitotic progression in cells released into AZD 1775. (**H**) The percentage of cells with midzone localization of INCENP. (**I**) The percentage of cells that had begun NER. (**J**) The percentage of cells with midbody localization of INCENP. (**K**) Rate of micronucleation at 3 h post-release. Data for all quantifications are presented as mean ± SD, *n* = 3 biological replicates (at least 100 cells counted at each time point for each replicate). Statistical analysis was performed on the area under the curve (AUC) by Student’s *t*-test. Source data are available online for this figure.

To investigate the functional impact of PKMYT1’s dynamic regulation during this critical stage of mitosis, we arrested cells at metaphase using an APC/C inhibitor. The arrested cells were then released into fresh media containing either DMSO, a PKMYT1 inhibitor (RP 6306), or a WEE1 inhibitor (AZD 1775) (Fig. 2A). Given that both PKMYT1 and WEE1 inhibit CDK1, we used the WEE1 inhibitor to test whether these kinases have a similar function during mitosis (Fig. 2B–K). Cells were stained for INCENP (a component of the chromosomal passenger complex) and Lamin B to visualize anaphase onset and nuclear envelope reformation (NER), respectively. Anaphase onset was defined by the initiation of INCENP relocalization from the kinetochores to the spindle midzone (Fig. 2B,C). In all treatment conditions, mitotic cells successfully initiated chromosome segregation, which was accompanied by NER and the expected relocalization of INCENP (Fig. 2C). However, inhibiting PKMYT1 at metaphase resulted in an accelerated anaphase onset, as evidenced by premature relocalization of INCENP to the midzone compared to DMSO-treated controls (Fig. 2D). This phenotype is unlikely to be driven by off-target effects as the timing of INCENP relocalization was unaffected by RP 6306 treatment in asynchronous PKMYT1 −/− cells (Appendix Fig. S4A,B). In contrast, WEE1 inhibition did not induce premature anaphase onset; the rate of INCENP relocalization was similar to that observed in DMSO-treated cells (Fig. 2H).

We observed a further functional distinction between PKMYT1 and WEE1 during late mitosis. While inhibition of PKMYT1 had no effect on the relocalization of INCENP to the midbody, WEE1 inhibition caused a significant delay (Fig. 2F,J). This observation is consistent with the known localization of WEE1 to the midbody bridge (Baldin and Ducommun, 1995). Despite these divergent effects on late-mitotic events, inhibition of either PKMYT1 or WEE1 resulted in a significant increase in the micronucleation rate (Fig. 2G,K). In other mitotic events, we found no significant differences in the NER rate when cells were treated with either the PKMYT1 or WEE1 inhibitors (Fig. 2E,I).

**Figure 3:**
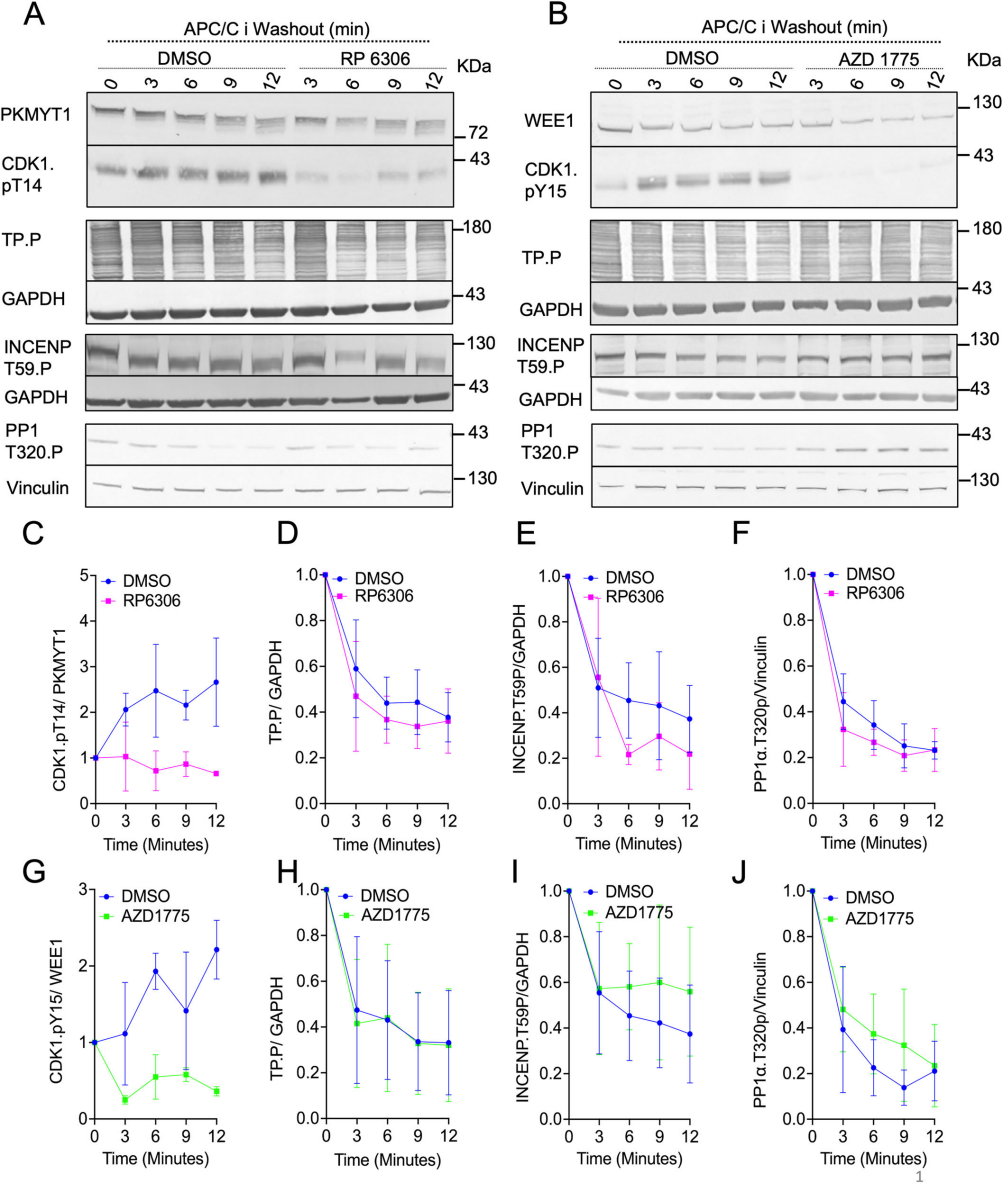
Inhibiting mitotic PKMYT1 accelerates anaphase onset. (**A**,** B**) Western blot analysis of mitotic exit kinetics. Mitotic hTERT-RPE1 cells were synchronized as described in (Fig. 2A) and released into fresh media containing either the PKMYT1 inhibitor RP 6306 (**A**) or the WEE1 inhibitor AZD 1775 (**B**), alongside a DMSO control. Cell lysates for each condition were collected at 3-min intervals, quantified for equal protein loading, and separated by 12% SDS-PAGE. For RP 6306-treated samples (**A**), PKMYT1 and pT14-CDK1 were detected on the same membrane. For AZD 1775-treated samples (**B**), WEE1 and pY15-CDK1 were detected on the same membrane. Additional parallel membranes were processed for each experiment to monitor the overall threonine-proline phosphorylation signal (TP.P), pT59-INCENP, and pT320-PP1, with GAPDH and Vinculin serving as loading controls for their respective membranes. (**C**–**F**) Quantification of phosphorylation signals from the western blots in (**A**). (**C**) pT14-CDK1 relative to total PKMYT1 levels. (**D**) TP.P relative to GAPDH. (**E**) INCENP T59p relative to GAPDH. (**F**) PP1.T320p relative to Vinculin. (**G**–**J**) Quantification of phosphorylation signals from the western blots in (**B**). (**G**) pY15-CDK1 relative to total WEE1 levels. (**H**) TP.P relative to GAPDH. (**I**) INCENP T59p relative to GAPDH. (**J**) PP1.T320p relative to Vinculin. For all quantifications, data represent mean ± SD from *n* = 3 biological replicates. The value of each replicate was normalized to the *t* = 0 time point. Source data are available online for this figure.

To better understand the rapid anaphase onset observed upon PKMYT1 inhibition, we monitored CDK1 activity using the experimental setup shown in Fig. 2A. Cell lysates were collected at 3-min intervals following metaphase release and analyzed for key phosphosites (Fig. 3A–J). We probed for CDK1 phospho-Y15 and phospho-T14, overall threonine/proline (TP) phosphorylation, and phosphorylation of key CDK1 substrates: INCENP phospho-T59 and PP1 phospho-T320 (Fig. 3A,B). As anticipated, PKMYT1 inhibition reduced the CDK1 phospho-T14 signal, while WEE1 inhibition reduced the CDK1 phospho-Y15 signal (Fig. 3C,G). In DMSO-treated cells, both overall TP phosphorylation (Fig. 3D,H) and phosphorylation at specific CDK1 sites (Fig. 3E,F,I,J) decreased during metaphase and anaphase, consistent with the drop in CDK1 activity that follows Cyclin B1 degradation (Murray and Kirschner, 1989; Murray et al, 1989). Notably, WEE1 inhibition resulted in a prolonged INCENP phospho-T59 and PP1 phospho-T320 signal compared to controls (Fig. 3I,J). In contrast, PKMYT1 inhibition resulted in a different phenotype: CDK1-Cyclin B1 activity appeared to decrease more rapidly than in control cells, as evidenced by reduced overall TP phosphorylation and specific CDK1 TP phosphorylation sites (Fig. 3D–F). This biochemical outcome aligns with the premature anaphase onset observed by immunofluorescence.

These results demonstrate that inhibiting PKMYT1 during metaphase accelerates anaphase onset, likely via the regulation of CDK1-Cyclin B1 activity. In human cells, CDK1 activity typically peaks at metaphase and declines sharply as Cyclin B1 is degraded by the APC/C^CDC20^ (King et al, 1996; Gavet and Pines, 2010). This degradation is a tightly regulated, sequential process where Cyclin B1 disappears first from the chromosomes, then the centromeres and spindle, and finally the cytoplasm (Clute and Pines, 1999; Wolf et al, 2006; Cirillo et al, 2024). While this global decline is essential for mitotic exit, a specific pool of CDK1-Cyclin B1 is reported to be transiently maintained in the cytoplasm and at the midzone (Afonso et al, 2014; Afonso et al, 2019; Maiato et al, 2015). Our results indicate that PKMYT1 may be the key regulator of this residual CDK1-Cyclin B1 pool during the metaphase-to-anaphase transition.

The observation that inhibiting PKMYT1 accelerates chromosome segregation may seem counterintuitive given its established role in inhibiting CDK1 to maintain G_2_ arrest. However, several mechanistic possibilities warrant investigation. One potential explanation is that the accelerated mitotic exit stems from localized CDK1 hyper-activation, which triggers a premature negative feedback loop. For instance, CDK1 can facilitate the phosphorylation of APC3, thereby promoting the assembly of the APC/C^CDC20^ and the subsequent degradation of Securin and Cyclin B1 (Kraft et al, 2003; Fujimitsu et al, 2016). In this model, the increase in CDK1 activity upon mitotic PKMYT1 inhibition shifts the biochemical equilibrium, accelerating the APC/C assembly that drives mitotic exit.

Alternatively, PKMYT1 may regulate mitotic exit by modulating components of the SAC. Under this hypothesis, PKMYT1 could influence the biochemical equilibrium required for SAC robustness; a perturbation in this balance may weaken the SAC by altering CDK1/PLK1-mediated phosphorylation of BUBR1, which is essential for the kinetochore recruitment of PP2A-B56 (Kruse et al, 2013; Song et al, 2024; Qi et al, 2006). Such direct modulation would impair the checkpoint’s ability to delay anaphase, leading to the accelerated transition and subsequent genomic instability observed in our subsequent analyses. Finally, PKMYT1 might also regulate CDH1, the APC/C co-activator that drives mitotic exit from anaphase onwards and is not subject to SAC inhibition.

### PKMYT1 and WEE1 inhibition in mitosis induces micronucleation and activates cGAS-STING signaling

**Figure 4:**
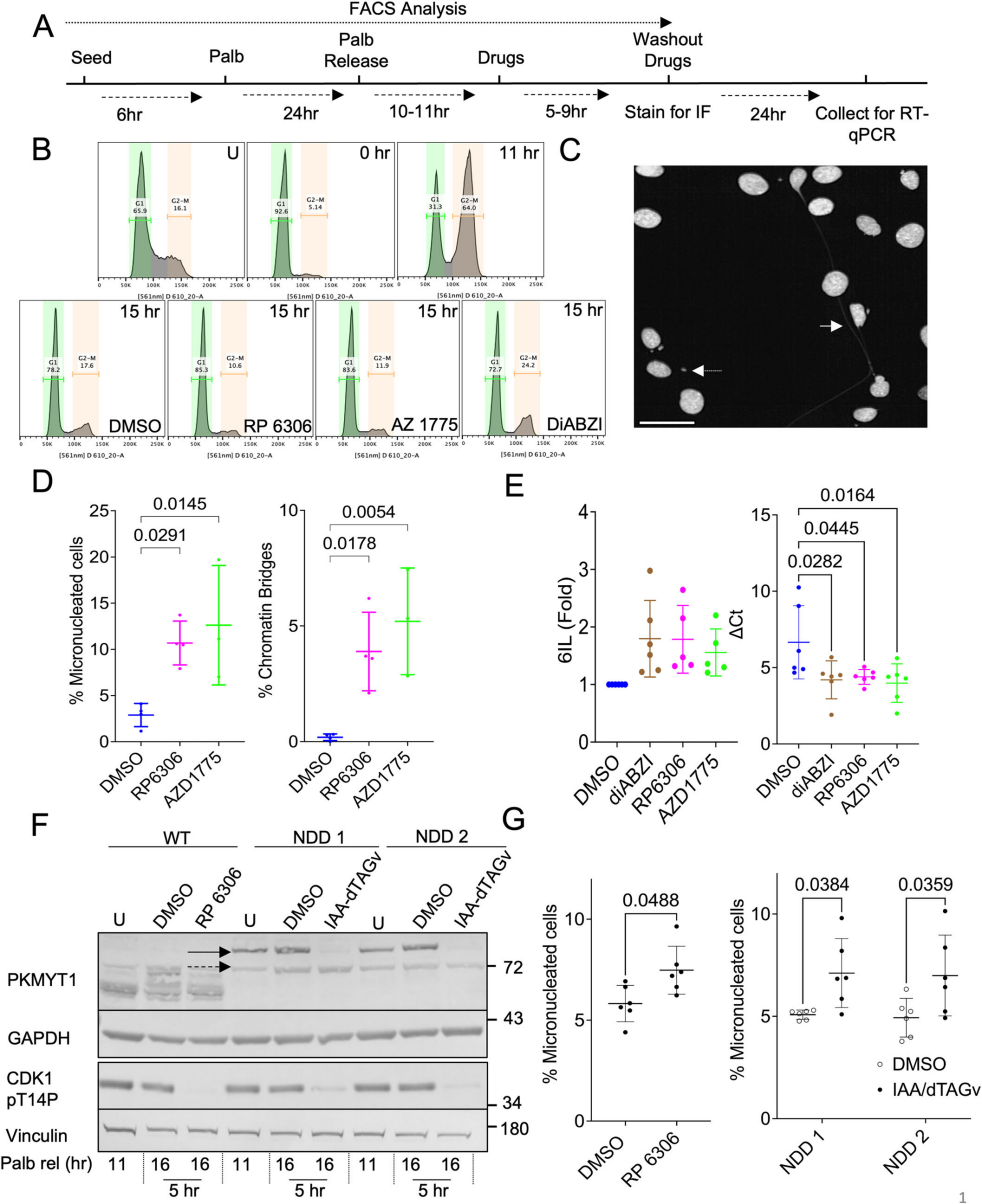
Mitotic PKMYT1 and WEE1 inhibition induces micronucleation and activates cGAS-STING signaling. (**A**) Outline of the experimental workflow for palbociclib synchronization of RPE1 cells. (**B**) Cell cycle analysis of RPE1 cells released from a 24-h palbociclib (150 nM) block, as determined by propidium iodide (PI) FACS analysis. Numbers next to the plots indicate hours post-release, with “U” representing an untreated asynchronous population. (**C**) Representative confocal images of RPE1 cells showing micronucleation (dotted arrow) and chromatin bridges (solid arrow). Cells synchronized as in (**A**) were treated with DMSO, RP 6306, or AZD 1775 for 9 h (from 11 to 20 h post-palbociclib release). Cells were pulsed with EdU to visualize DNA. Scale bar, 20 μm. (**D**) Quantification of the micronucleation and chromatin bridges as shown in (**C**). Data were presented as mean ± SD, *n* = 3–4 biological replicates. One-way ANOVA was used for statistical analysis. (**E**) RT-qPCR analysis of IL-6 gene induction. Cells were exposed to DMSO, DiABZI, AZD 1775, or RP 6306 for 5 h (from 11 to 16 h post-palbociclib release). The drugs were then washed out, and the cells were allowed to progress to the next cell cycle before total RNA was isolated. Data were presented as mean ± SD, *n* = 6 biological replicates. One-way ANOVA was used for statistical analysis. (**F**) RPE1 wild-type (WT) cells and two RPE1 clones with a degron-tagged PKMYT1 (clones NDD 1 and NDD 2) were synchronized as in (**A**). WT cells were treated with either DMSO or RP 6306, and NDD clones were treated either with DMSO or 0.5 μM 5-Ph-IAA - 0.1 μM dTAGv for 5 h after 11 h post-palbociclib release. “U” represents an untreated population. Cell lysates were collected at 11 h post-palbociclib release (before drug treatments) or 16 h post-palbociclib release (5 h of drug treatments). Equal protein amounts were separated by 12% SDS-PAGE and immunoblotted. PKMYT1 and pT14-CDK1 were detected on separate membranes, with GAPDH and Vinculin serving as their respective loading controls on the same membrane. The solid arrow indicates the tagged PKMYT1, and the dotted arrow indicates a non-specific band. (**G**) Visual quantification of micronucleation in WT cells or NDD clones, treated as indicated in (**F**). Data were presented as mean ± SD, *n* = 6 biological replicates. Statistical significance was determined using a Student’s *t*-test for comparisons involving WT cells, or a two-way ANOVA for comparisons involving NDD clones. Source data are available online for this figure.

To understand the consequences of inhibiting mitotic WEE1 and PKMYT1 on the subsequent cell cycle, we synchronized cells in G_1_ phase with the CDK4/6 inhibitor, palbociclib. The cells were then released into fresh media containing EdU, allowing them to progress through S-phase without perturbation (Fig. 4A). PKMYT1 or WEE1 inhibitors were added when the cells were accumulating at the end of G_2_-phase, as indicated by FACS analysis (Fig. 4B), and cells were allowed to complete mitosis and proceed into the next cycle. Immunofluorescence staining of DNA revealed a significant increase in both micronucleation and chromatin bridges following mitosis in cells treated with either the WEE1 or PKMYT1 inhibitor (Fig. 4C,D).

**Figure EV2:**
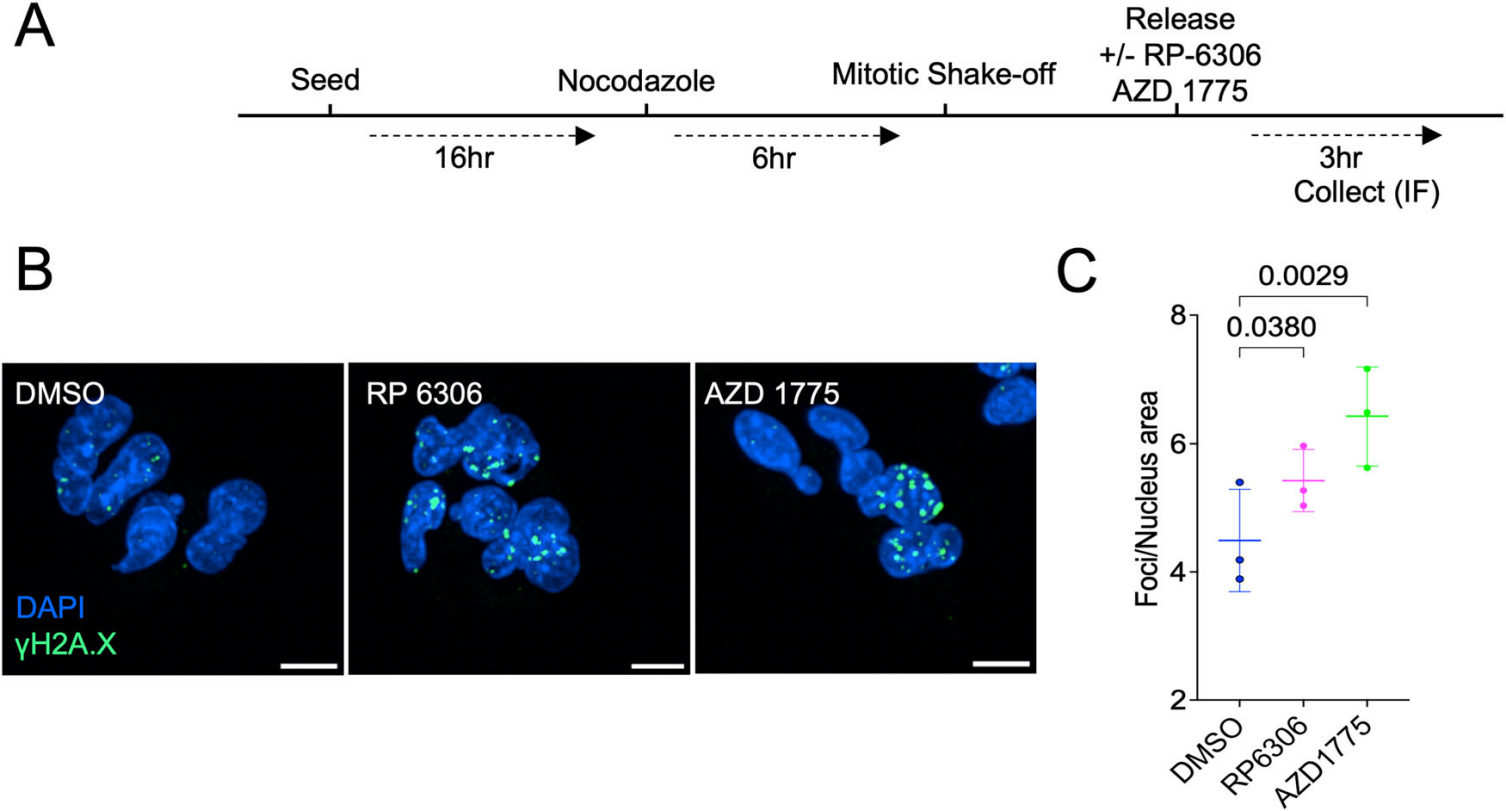
Mitotic PKMYT1 and WEE1 Inhibition Induces DNA Damage. (**A**) Outline of the experimental workflow for nocodazole shake-off of RPE1 cells. (**B**) Representative maximum intensity projections of confocal images of cells post-nocodazole release, exposed to either DMSO, RP 6306 (500 nM), or AZD 1775 (300 nM). Cells were stained with DAPI (DNA) and γH2A.X as an indicator for double-strand breaks. Scale bar, 10 μm. (**C**) Quantification of γH2A.X-positive foci per nucleus. Mean ± SD, *n* = 3 biological replicates is shown. One-way ANOVA was used for statistical analysis.

To confirm that the genomic instability was a direct consequence of mitotic dysfunction rather than G_2_-phase defects, we isolated mitotic RPE1 cells via nocodazole shake-off. These cells were released into fresh, prewarmed media containing either the WEE1 or PKMYT1 inhibitor (Fig. EV2A). Analysis of the resulting G_1_ population showed a significant elevation in γH2AX foci, a marker of DNA double-strand breaks. Consistent with our previous observations, inhibition of either PKMYT1 or WEE1 exclusively during mitosis resulted in a significant increase in DNA damage in the subsequent G_1_ phase (Fig. EV2B,C).

To determine whether the observed genomic instability triggers the cGAS-STING signaling pathway, cells were synchronized as described in Fig. 4A. Following the removal of inhibitors and completion of an additional cell cycle to allow for micronuclei formation and subsequent sensing, total RNA was isolated. RT-qPCR was performed to quantify the expression of *IL-6*, a pro-inflammatory cytokine and established marker of STING activation. Both the WEE1 and PKMYT1 inhibitors increased IL-6 transcription to a similar extent as the STING agonist, diABZI (Fig. 4E). We also noted that inhibiting PKMYT1 immediately after palbociclib release did not affect the rate of S-phase progression compared to the DMSO control, as indicated by the EdU staining pattern (Appendix Fig. S5A). However, consistent with other research (Li et al, 2024; Gallo et al, 2022), the addition of the PKMYT1 inhibitor at late G_2_ induced premature mitotic entry, as indicated by the Cyclin B1 phospho-S126 signal (Appendix Fig. S5B,C).

**Figure EV3:**
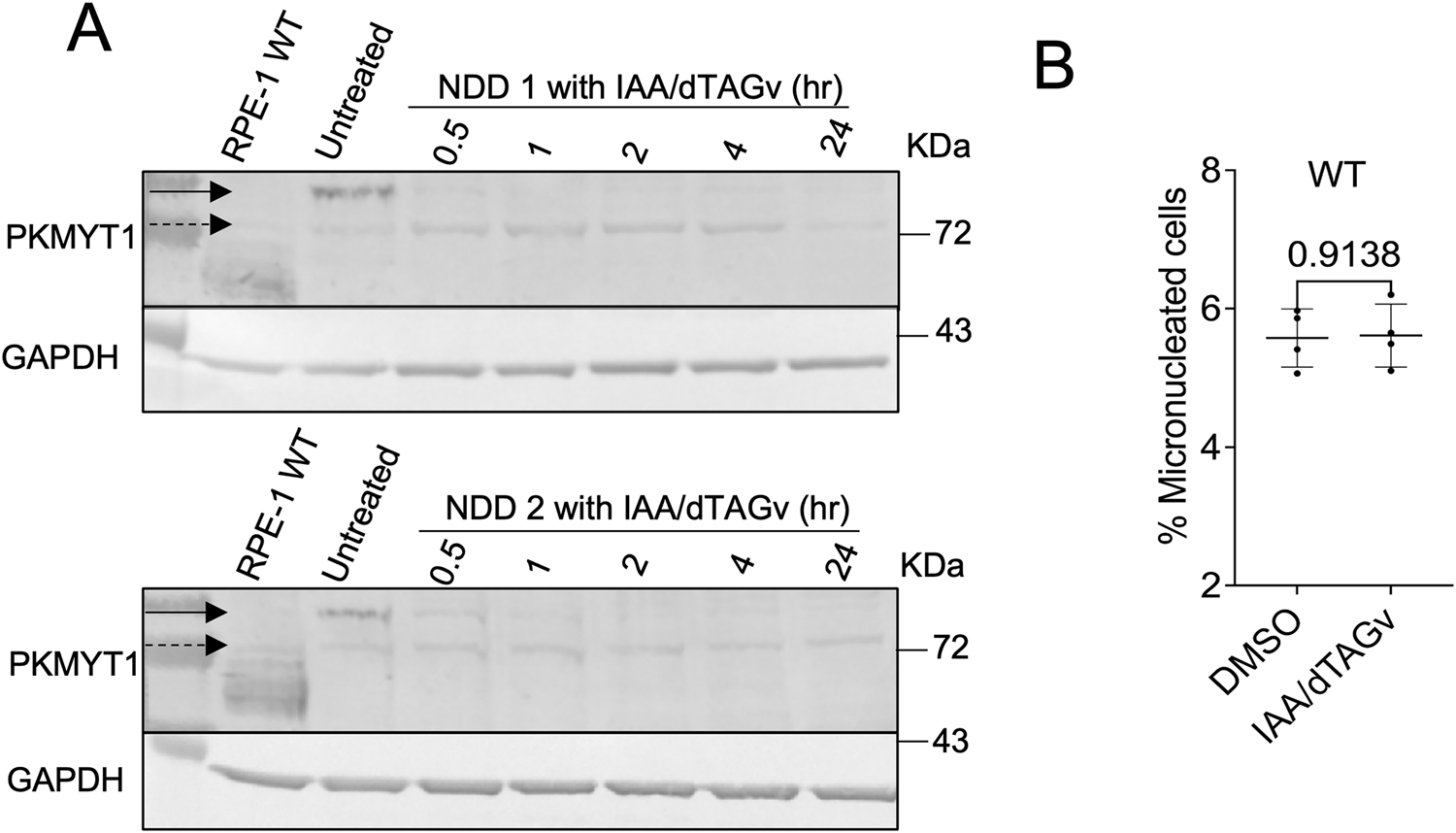
Use of a degron-tagged system to induce acute PKMYT1 degradation. (**A**) Acute degradation of degron-tagged PKMYT1 (clones NDD 1 and NDD 2). Cells with degron-tagged PKMYT1 were treated with 5-Ph-IAA-dTAGv for the indicated time points. Cell lysates were collected, separated by 12% SDS-PAGE, and immunoblotted for PKMYT1 and GAPDH detected on the same membrane to serve as a loading control. The solid arrow indicates the tagged PKMYT1, and the dotted arrow indicates a non-specific band. (**B**) Rate of micronucleation of WT RPE1 cells treated with IAA-dTAGv. Untagged RPE1 cells were synchronized with palbociclib for 24 h and then released into fresh medium. After 11 h, cells were treated with either DMSO or 5-Ph-IAA -dTAGv for 5 h. Cells were then fixed and stained for DNA to quantify micronucleation. Data represent the mean ± SD, *n* = 4 biological replicates. Statistical significance was determined using a Student’s *t*-test.

To establish whether there was a causal link between PKMYT1 loss and micronucleation, we generated RPE1 cell lines utilizing an auxin-inducible degron (AID) system for the rapid and selective depletion of PKMYT1. Treatment with 5-Ph-IAA/dTAGv resulted in a robust reduction of PKMYT1 protein levels within 1–2 h (Fig. EV3A). To investigate the consequences of PKMYT1 removal, two independent clones (NDD 1 and NDD 2) were synchronized in G_1_ with Palbociclib and released into fresh media. After 11 h, 5-Ph-IAA/dTAGv was added to induce PKMYT1 degradation as cells entered late G_2_ (Fig. 4F,G). Similar to our chemical inhibition results, the acute depletion of PKMYT1 led to a significant increase in micronucleation (Fig. 4G). Importantly, no increase in micronucleation was observed when the degradation ligands were added to untagged RPE1 cells, confirming the specificity of the system (Fig. EV3B). Western blot analysis further confirmed that PKMYT1 depletion during mitotic progression directly correlated with a reduction in CDK1 T14 phosphorylation (Fig. 4F).

**Figure EV4:**
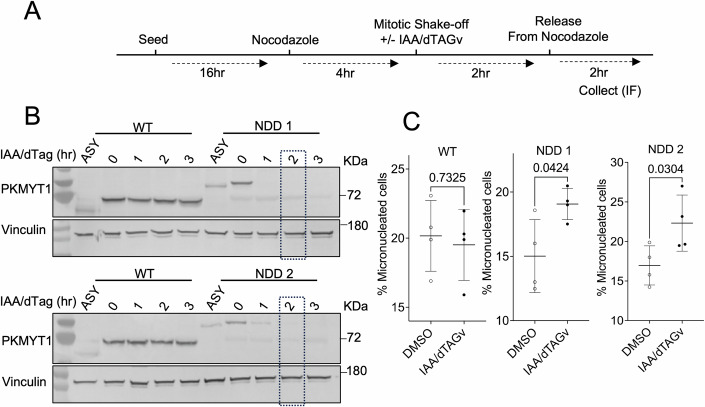
Use of a dual-degron system to induce acute mitotic PKMYT1 degradation. (**A**) Outline of the experimental workflow for nocodazole shake-off of RPE1 cells. RPE1 wild-type (WT) and two RPE1 clones expressing degron-tagged PKMYT1 (clones NDD 1 and NDD 2) were synchronized as indicated. (**B**) WT and NDD clones were treated with DMSO or a ligand (0.5 μM 5-Ph-IAA and 0.1 μM dTAGv). Cell lysates were collected every hour for 3 h while cells remained arrested in nocodazole. Equal amounts of protein lysate were resolved by 12% SDS-PAGE. Membranes were immunoblotted for PKMYT1, while Vinculin was detected on a parallel membrane to serve as a sample processing control. ASY denotes asynchronous cells. (**C**) Quantification of the micronucleation rate in WT and NDD clones post-nocodazole washout. WT and NDD clones were synchronized as in (**A**) and treated with DMSO or the degradation ligand (0.5 μM 5-Ph-IAA and 0.1 μM dTAGv) for 2 h during the arrest. Cells were then released into fresh media to allow for mitotic progression. After 2 h of nocodazole washout, cells were fixed and stained with DAPI to visualize micronucleation. Data represent the mean ± SD, *n* = 4 biological replicates. Statistical significance was determined using a Student’s *t*-test.

To further exclude G_2_-phase contributions, we employed a mitotic shake-off approach. After determining that PKMYT1 levels were significantly depleted within 2 h in prometaphase-arrested cells (Fig. EV4A,B), isolated mitotic cells were treated with degradation ligands while maintaining the arrest. Upon release into fresh media, both NDD 1 and NDD 2 clones—but not WT cells—exhibited a marked increase in micronucleation two hours post-release (Fig. EV4C). Collectively, these results demonstrate that the loss of PKMYT1 specifically during mitosis is sufficient to drive genomic instability.

The observation that mitotic PKMYT1 loss drives micronucleation and subsequent cGAS-STING activation highlights its essential role in governing mitotic progression and preserving genomic integrity.

### Inhibiting mitotic PKMYT1 causes chromosome missegregation and weakens the spindle assembly checkpoint

**Figure 5:**
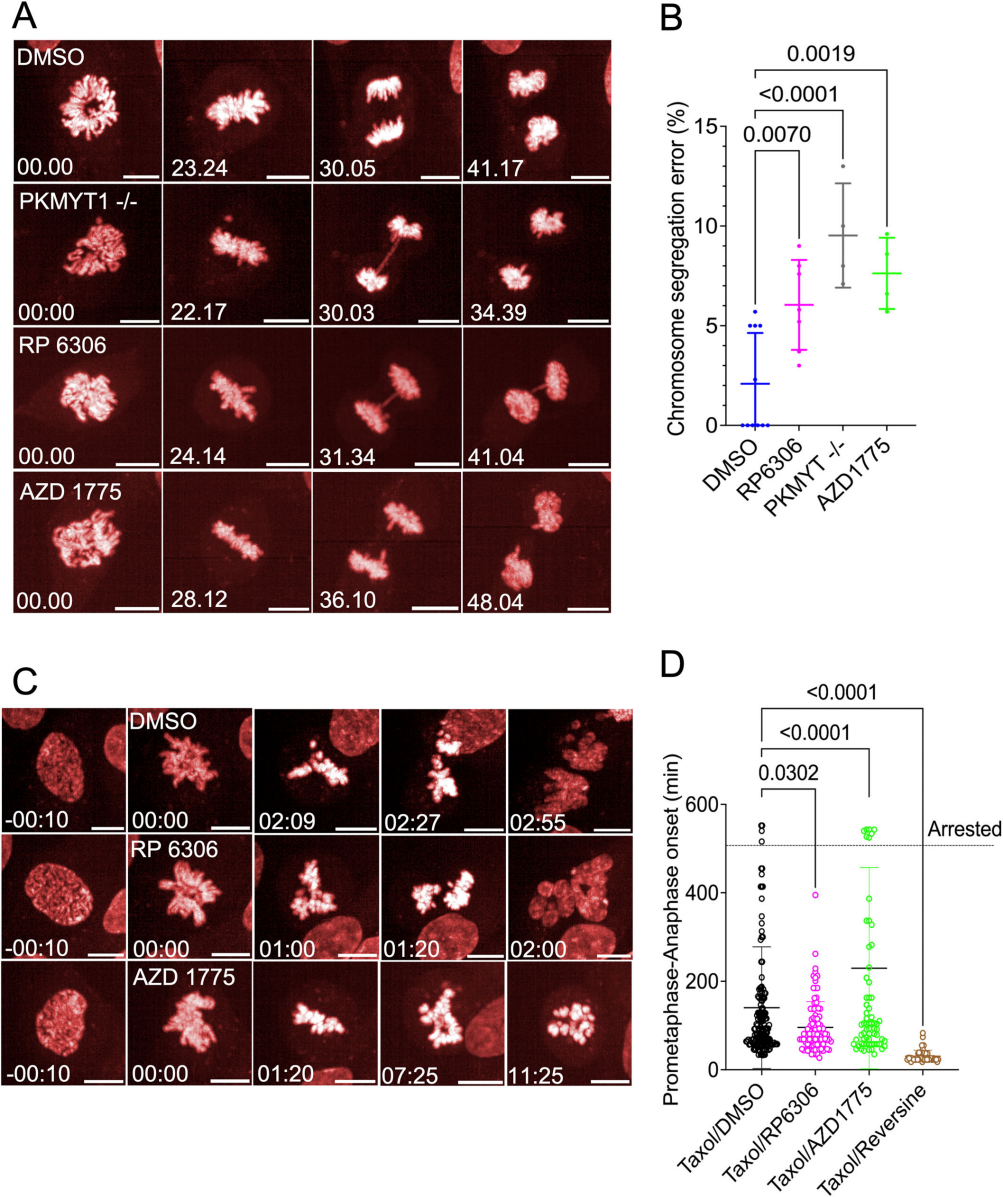
Inhibiting mitotic PKMYT1 causes chromosome missegregation and weakens the spindle assembly checkpoint. (**A**) Representative maximum intensity projections of confocal images over time of an RPE1 progressing through mitosis. Cells were stained with SiR-DNA 2 h before imaging. Immediately prior to imaging, cells were treated with DMSO, RP 6306, or AZD 1775. To minimize the laser toxicity, images were acquired at 4–7 min intervals for up to 1 h using an Opera confocal microscope maintained at 37 °C and 5% CO_2_. (**B**) Quantification of chromosome segregation defects from the live-cell imaging described in (**A**). Only cells that were in prophase at the start of imaging were included in the quantification. Data are presented as mean ± SD, *n* = 4–7 biological replicates, with a minimum of 100 cells, followed in total. One-way ANOVA was used for statistical analysis. (**C**) Representative maximum intensity projections of confocal images over time of an RPE1 going through mitotic slippage. Cells were stained with SiR-DNA for 2 h and then treated with 5 nM paclitaxel to induce prometaphase arrest. After 1 h of initial imaging, fresh media containing either DMSO, RP 6306, AZD 1775, or reversine along with paclitaxel (5 nM) was added. To determine the time of mitotic slippage, cells were then imaged for an additional 9–11 h with images taken at 10–15 min intervals. Imaging was performed on an Opera confocal microscopy system maintained at 37 °C and 5% CO_2_. (**D**) Quantification of the time from prometaphase arrest to mitotic slippage in the single-cell assay described in (**C**). Data for DMSO and RP 6306 represent the mean ± SD, *n* = 5 biological replicates, with at least 100 cells followed in total. For AZD 1775, data represent mean ± SD, *n* = 3 biological replicates with 75 cells, followed in total. For the reversine condition, data represent mean ± SD, *n* = 2 biological replicates with 45 cells, followed in total. One-way ANOVA was used for statistical analysis. Scale bar, 10 µm. Source data are available online for this figure.

To investigate the impact of inhibiting PKMYT1 or WEE1 on mitosis without the complication of other drug treatments, we assayed asynchronous RPE1 cells by live-cell imaging. PKMYT1 or WEE1 inhibitors were added immediately prior to imaging, and only cells already in prophase at the time of drug addition were included in the analysis. Despite apparently typical progression through mitosis, inhibition of either kinase resulted in a significant increase in chromosome missegregation (Fig. 5A,B). The effect of PKMYT1 inhibition was further validated in a PKMYT1 knockout cell line, which also showed an increase in chromosome segregation errors relative to wild-type cells (Fig. 5A,B). These observations indicate that PKMYT1 is required for SAC-mediated regulation, as its inhibition allows anaphase to proceed despite improper chromosome attachments. While WEE1 has been previously reported to contribute to SAC strength (Visconti et al, 2015), our findings indicate that PKMYT1 may also play a role in governing the timing of anaphase onset by modulating checkpoint robustness.

To test whether inhibiting PKMYT1 also impacts SAC strength, we treated cells with a low concentration of paclitaxel, sufficient to activate the SAC without causing prolonged arrest, and assayed the length of mitotic delay by time-lapse microscopy (Ikui et al, 2005). To ensure that the observed phenotypes were due specifically to mitotic activity, cells were first allowed to arrest in mitosis before the addition of either a WEE1 or PKMYT1 inhibitor (Fig. 5C). Their effects were compared to DMSO or an MPS1 inhibitor as negative and positive controls, respectively. Consistent with previous literature, the WEE1 inhibitor prolonged mitotic arrest compared to the DMSO control. In contrast, the PKMYT1 inhibitor accelerated mitotic exit, albeit not as rapidly as treatment with the MPS1 inhibitor (Fig. 5D). To determine whether this acceleration stemmed from structural defects in the spindle, we examined the tubulin cytoskeleton in cells that escaped the paclitaxel-induced arrest. Both DMSO- and PKMYT1 inhibitor-treated cells exhibited comparable tubulin structures, indicating that the accelerated exit was not a secondary consequence of spindle destabilization (Appendix Fig. S6A). Instead, these data indicate that PKMYT1 inhibition directly interferes with the regulatory signaling of the checkpoint machinery itself.

We conclude that while both PKMYT1 and WEE1 are required for faithful chromosome segregation, they appear to operate through distinct mechanistic pathways to govern mitotic exit and checkpoint maintenance.

Despite their shared roles as CDK1 inhibitory kinases at the G_2_/M transition, PKMYT1 and WEE1 inhibition produce strikingly different mitotic phenotypes. While PKMYT1 inhibition accelerates mitotic exit and SAC slippage, WEE1 inhibition results in prolonged arrest and late-mitotic delays. These divergent effects can be attributed to their unique mechanistic and spatial properties. Mechanistically, WEE1 is restricted to phosphorylating CDK1 on Y15, whereas PKMYT1 is a dual-specificity kinase with a strong preference for T14 (Watanabe et al, 1995; Liu et al, 1997). These distinct phosphorylation patterns likely result in differing kinetics of CDK1 inhibition.

Spatially, PKMYT1 is a membrane-associated kinase localized to the ER and Golgi, whereas WEE1 is predominantly nuclear, relocating to the cytoplasm in prophase and the midbody bridge in late mitosis (Liu et al, 1997; Baldin and Ducommun, 1995). This spatial segregation provides distinct microenvironments where each kinase is governed by separate local regulatory networks, reinforcing their non-redundant roles in governing mitotic exit and checkpoint maintenance.

## Conclusion

Our results have uncovered a previously undescribed mitotic function for PKMYT1, moving beyond its established canonical role with WEE1. We found that PKMYT1 is involved in regulating the timing of sister chromatid separation. Notably, inhibiting mitotic PKMYT1 leads to premature anaphase onset that has significant consequences for genomic integrity, as evidenced by the increased formation of micronuclei. Furthermore, our observations point toward a possible role for PKMYT1 in preventing mitotic slippage in the presence of anti-microtubule poisons, indicating it is necessary to maintain the checkpoint delay required for accurate cell division.

Collectively, we have identified a previously unrecognized role for PKMYT1 as a regulator of CDK1-Cyclin B1 activity during the metaphase-to-anaphase transition. This control layer operates alongside established Cyclin B1 degradation to ensure mitotic fidelity. While further study is required to dissect the exact biochemical pathways involved, these findings reveal that mitotic PKMYT1 is a key factor in preventing accelerated mitotic exit and subsequent genomic instability.

## Methods

**Table:** Reagents and tools table

Reagent/resource	Reference or Source	Identifier or Catalog Number
**Experimental models**		
hTERT-RPE1	ATCC	CRL-4000
HeLa	ATCC	CCL-2
**Recombinant DNA**		
eSpCas9(1.1)_No_FLAG_ATP1A1_G3_Dual_sgRNA	Addgene plasmid	86613
pcDNA5/FRT/TO	Thermo Fisher Scientific	V652020
**Antibodies**		
Rabbit anti-PKMYT1	Cell Signalling	4282
Rabbit anti-PKMYT1	Thermo Fisher Scientific	A302-424A
Rabbit anti-pT14-CDK1/2/3	Abcam	ab32384
Rabbit anti-WEE1	Cell Signalling	13084
Rabbit anti-pY15 Cdc2	Cell Signalling	9111
Rabbit anti-GAPDH	Cell Signalling	2118
Rabbit anti-Vinculin	Abcam	ab207440
Rabbit anti-Cdk1	Cell Signalling	77055
Mouse anti-INCENP	Abcam	ab23956
Rabbit anti-INCENP T59P	Antibodies	AB307146
Rabbit anti-PP1 T320P	Cell Signalling	25815
Mouse anti-P-Thr-Pro	Cell Signalling	9391S
Rabbit anti-Lamin B	Abcam	ab16048
Rabbit anti-Cyclin B1 S126P	Abcam	ab133439
Goat anti-Mouse IgG Alexa Fluor 488	Invitrogen	A32723
Goat anti- Rabbit IgG Alexa Fluor 546	Thermo Fisher Scientific	A-11010
Mouse anti-Tat1 (Tubulin)	a gift from K. Gull (Woods et al, 1989)	N/A
DAPI	Invitrogen	D1306
EdU	Thermo Fisher Scientific	C10337
SiR-DNA	Universal Biologicals	SC007
**Chemicals, enzymes, and other reagents**		
Trypsin-EDTA	Gibco	15400
Dulbecco’s modified eagle medium (DMEM)	Sigma-Aldrich	D6546
Fetal bovine serum (FBS)	Gibco	10270
Penicillin streptomycin	Gibco	15140
GlutaMAX	Gibco	35050
Sodium pyruvate	Gibco	11360070
24-well sensoplate	Greiner	662892
Nocodazole	Sigma-Aldrich	M1404
poly-L-lysine	Sigma-Aldrich	P4707
RP 6306	MedChemExpress	HY-145817A
AZD 1775	Selleckchem	S1525
DMSO	Avantor	ICNA0219605580
diABZI	Selleckchem	S8796
Reversine	Cambridge BioScience	CAY10004412
Paclitaxel	Selleckchem	S1150
Eg5 Inhibitor III	Sigma-Aldrich	324622
proTAME	Cambridge Bioscience	HY-124955
5-PH-IAA	HY-134653	MedChemExpress
dTAGv1	HY-145514D	MedChemExpress
Lipofectamine	Invitrogen	18324010
Plus Reagent	Invitrogen	11514015
Apcin	Selleckchem	S9605
Palbociclib	Sigma-Aldrich	PD-0332991
Propidium iodide	Sigma-Aldrich	P4170
Doxycycline	Scientific Laboratory	D3072
Zeocin	Gibco	R25005
Ouabain	Sigma-Aldrich	O3125
Hygromycin B	Invitrogen	10687010
Geneticin	Gibco	10131035
DirectPCR lysis reagent	Viagen Biotech	302-C
Proteinase K	Apollo Scientific	BIP4206
JumpStart RedTaq ReadyMix	Sigma-Aldrich	P0982
NEB lysis buffer	Cell Signalling	NEB 9803S
cOmplete protease inhibitor	Merck	11697498001
Phosstop tablet	Merck	4906845001
Bio-Rad reagent	Bio-Rad	500-0006
PMSF	Sigma-Aldrich	93482
12% bis-tris gels	Thermo Fisher	NP0343
Nitrocellulose Membrane	AMERSHAM	10600003
PVDF	Immobilon	IPVH00010
ProLong Diamond Antifade Mounting Media	Invitrogen	P36965
H2A.X	Merck	05-636
0.2% Triton X-100	Sigma-Aldrich	X-100
TRIzol™ Reagent	Thermo Fisher	15596026
Fast SYBR™ Green Master Mix	Thermo Fisher	4344463
High-Capacity RNA-to-cDNA™ Kit	Thermo Fisher	4387406
BSA	Sigma-Aldrich	A7638
**Software**		
CRISPOR	N/A	http://crispor.tefor.net/crispor.py
**Other**		
Neon transfection system	Invitrogen	MPK5000

### Plasmid construction

To generate the stable PKMYT1-mAID-FKBP12 cell line, we employed a co-selection strategy. The OsTIR1(F74G) and zeocin resistance genes were simultaneously inserted into the safe harbor AAVS site, while the mAID-FKBP12 double degron was inserted at the C-terminus of endogenous PKMYT1. Zeocin selection was then used to enrich for correctly modified clones.

The Cas9 plasmid used was a modified variant of eSpCas9-No-FLAG-ATP1A1-G3-Dual-sgRNA (Addgene plasmid #86613, gift from Yannick Doyon). This plasmid was altered by fusing the C-terminus of the eSpCas9 enzyme to amino acids 1–110 of human Geminin to create eSpCas9-hGeminin (Addgene plasmid #199344, Morrison et al, 2023). The G3 ATP1A1 guide was replaced with a guide to the AAVS safe harbor site (5’-GGGGCCACTAGGGACAGGAT-3’), and a second sgRNA targeting the C-terminus of PKMYT1 (5’-GCAGAGTCTGGGGCTCAGGT-3’) was added between two BbsI sites (esPcas9-hGeminin-AAVS-PKMYT1). All sgRNAs were designed using CRISPOR software with 20 bp guides selected within 100 bp of the target region. This plasmid enabled the simultaneous targeting of two separate cassettes.

The template plasmid for inserting OsTIR1(F74G) into the AAVS site contained a cassette with the OsTIR1(F74G) gene (sequence from Yesbolatova et al, 2020, ordered from GeneArt), an FRT site, and zeocin resistance (amplified from pFRT/lacZeo, Invitrogen), all flanked by homology arms to the AAVS safe harbor site (AAVS- OsTIR1(F74G)-ZEO-FRT). The template plasmid for the double degron insertion (PKMYT1-C-FKBP12-mAID) contained a cassette with both FK506-binding protein 12 (FKBP12) and the auxin-inducible degron (mAID, Yesbolatova et al, 2020), flanked by homology arms to the C-terminus of PKMYT1 (GeneArt).

PKMYT1 was deleted using another modified version of eSpCas9-hGeminin. This plasmid contained the G2 sgRNA (5’-CATCCAAGCTGCTACAGAAG-3’, Agudelo et al, 2017) in place of G3, and a sgRNA to PKMYT1 (5’-GGGCCATGGCTCCTACGGAG-3’, Gallo et al, 2022) inserted between two BbsI sites (eSpCas9-hGeminin-G3-MYT1del).

### Cell culture

RPE1 and HeLa cells were cultured in high-glucose DMEM supplemented with 10% FBS, 1% GlutaMAX, and 1% Penicillin/Streptomycin. Cells were maintained at 37 °C and 5% CO_2_ in a humidified incubator. Cultures were kept at less than 70% confluency and passaged no more than ten times prior to experiments.

Cell lines were routinely screened for mycoplasma contamination through the Cancer Research UK Manchester Institute Molecular Biology Core Facility (CRUK MI MBCF). Only mycoplasma-free cultures were used for the experiments reported in this study.

### Cell transfection

To create the degron lines, RPE1 cells were harvested by trypsinization using 0.05% Trypsin-EDTA. About 5 × 10^6^ cells were co-transfected with three plasmids: esPcas9-hGeminin-AAVS-PKMYT1, AAVS-OsTIR1(F74G)-ZEO-FRT, and PKMYT1-C-FKBP12-mAID, using 10 μL of each plasmid all at 1 μg/μL. Cells were electroporated using the Neon Transfection System at 1050 V, 30 ms, two pulses. Electroporated cells were incubated in 15 cm round dishes for 24 h, then treated with 10 μM Zeocin. Media and drugs were replaced every few days for 14–20 days. Colonies were picked and placed in 12-well plates, then expanded into six-well plates for cryopreservation and DNA analysis.

For the PKMYT1 deletion lines, cells were electroporated as above with 10 μg of eSpCasp-hGeminin-G3-MYT1del. They were then treated with 2 μM ouabain. Media and drugs were replaced every few days for 7–14 days before being expanded as described above.

### Cell line screening

To screen for successfully modified cell lines, cell pellets were lysed in PCR direct lysis reagent plus Proteinase K for 18 h at 55 °C, then 95 °C for 1 h. DNA was precipitated for 30 min at 4 °C with 0.3 M NaCl and 40% Isopropanol, pelleted at 4 °C for 15 min and washed once with 70% ethanol before resuspending in 40 μL H2O. About 1 μL of this was used in a 10 μL PCR reaction using JumpStart RedTaq ReadyMix with primers F: 5’-CAGAGCTGGTTTCATCCG-3’ and R: 5’-ACATGAGCAAGCTTGGGT-3’ to amplify the C-terminus, and F: 5’-CTGACTCCAAACTGCCTTGCTCTT-3’ and R: 5’-GTGCCAGACTCTGGACAA-3’ to amplify the region of the deletion guide using the following conditions: 95 °C for 1 min; 35 cycles of 95 °C 30 s, 56 °C 30 s, 72 °C 1 min; 72 °C 5 min. Gel-purified PCR products were sequenced by Sanger Sequencing (Genewiz).

### Degradation of PKMYT1

Degron-tagged PKMYT1 was degraded on the addition of 500 nM 5-PH-IAA and 100 nM dTAGv1.

### Generation of Flp-In T-REx cell lines

#### Flp-In T-REx HeLa cells

Flp-In T-REx HeLa cells, a gift from Stephen Taylor, were used to generate the stable cell line. A cassette containing the N-terminally 3xFLAG-tagged human PKMYT1 cDNA (GeneArt) was cloned into pcDNA5/FRT/TO using restriction cloning between HindIII and BamHI sites. To create the PKMYT1-Kinase dead mutant, N238A and D251A mutations were introduced into a 2xFLAG-tagged version of pcDNA5/FRT/TO-PKMYT1 using QuikChange II (Agilent Technologies) with the following primers: F: 5’-GGCCCCAGGAAGATGGCGGCAGGCTTGACATC-3’, R: 5’-GATGTCAAGCCTGCCGCCATCTTCCTGGGGCC-3’ for N238A; and F: 5’-CCAGCAGTCCGAAGGCACCCAGCTTGCAG-3’, R: CTGCAAGCTGGGTGCCTTCGGACTGCTGG-3’ for D251A.

The resulting vectors were co-transfected into Flp-In T-REx HeLa-FRT cells with the Flp recombinase-encoding plasmid pOG44 using Lipofectamine according to the manufacturer’s instructions. Briefly, 100 ng of pcDNA5/FRT/TO-FLAG-PKMYT1 and 900 ng of pOG44 were transfected into cells in a six-well plate using Lipofectamine and Plus Reagent. Twenty-four hours post-transfection, the cells were released with trypsin and transferred to a 10 cm dish. Subsequently, 200 µg/ml Hygromycin B was added. The media was changed every 3–4 days until single colonies were picked and expanded. 3xFLAG-PKMYT1 expression was induced using 1 μg/ml doxycycline.

#### Flp-In T-REx RPE-FRT cells

Flp-In T-REx RPE-FRT was a gift from Jon Pines. A cassette containing the N-terminally 3xFLAG-tagged human PKMYT1 cDNA (GeneArt) was cloned into pcDNA5/FRT/TO with hygromycin resistance switched to neomycin resistance (pcDNA5/FRT/TO-neo, a gift from Jon Pines) by restriction cloning between HindIII and BamHI sites. About 1 μg pcDNA5/FRT/TO-neo-PKMT1 and 9 μg pOG44 were co-transfected into Flp-In T-REx RPE-FRT cells by electroporation using the Neon Transfection System at 1050 V, 30 ms, two pulses. Electroporated cells were incubated in 15 cm round dishes for 24 h, then treated with 500 μg/ml Geneticin. Media and drugs were replaced every 3–4 days for 14–20 days. Single colonies were picked and expanded. 3xFLAG-PKMYT1 expression was induced using 1 μg/ml doxycycline.

### Cell synchronization and drug treatments

#### G1/S synchronization

To synchronize hTERT-RPE1 cells at G1/S, cells (2.5 × 10^5^) were seeded in 10-cm plates and cultured in DMEM with 10% serum for 6–7 h. Palbociclib (150 nM) was then added for 24 h to arrest the cells.

For release, cells were washed 3x with fresh, warm media. S-phase status was monitored by measuring 5-ethynyl-2’-deoxyuridine (EdU) incorporation using the Click-iT™ Plus EdU Alexa Fluor 488 Flow Cytometry Assay Kit according to the manufacturer’s instructions. A final concentration of 1 μM EdU was added 1 h post-release, and cells were fixed hourly for immunofluorescence up to 13 h after the initial release.

To measure the impact of the drug treatments following mitosis, cells were treated with either AZD 1775 (300 nM), RP 6306 (500 nM), diABZI (1 µM), or a DMSO. Drug treatments began at 11 h post-Palbociclib release and lasted for 5 h. Following treatment, cells were either fixed for immunofluorescence or the drugs were washed out with 3x fresh media. For subsequent RT-qPCR analysis, cells were allowed to progress through an additional cell cycle before collection.

#### G_2_/M synchronization

To synchronize cells at the G_2_/M boundary, RPE1 cells (2.5 × 10^5^) were seeded onto 10-cm plates and allowed to adhere for 6–7 h. Cells were then treated with 150 nM Palbociclib for 24 h to induce a G_1_ arrest. Following the G_1_ block, cells were washed twice with prewarmed fresh media and incubated for 10–11 h to allow progression into late S/G_2_. To specifically arrest cells at the G_2_/M transition, 10 µM RO-3306 was added for 6 h. Finally, cells were washed three times with fresh, prewarmed media to release them from the block before collection for FACS analysis.

#### Prometaphase synchronization

To synchronize cells at prometaphase, RPE1 cells (1 × 10^6^) were seeded on 15-cm plates and allowed to grow overnight. The following day, cells were treated with nocodazole (330 nM) for 6 h. Mitotic cells were then collected by firmly tapping the plates to dislodge rounded cells, and the shaken-off cells were collected in culture media. The cells were centrifuged and washed 3x with fresh, warm media before being re-seeded onto 10-cm plates. For experiments involving PKMYT1 inhibition, RP 6306 (500 nM), DMSO or IAA/dTAGv was added 2 h prior to the release of nocodazole. For Fig. 1A,B, the cell lysates were collected every 10 min. For Fig. 1F,G, the cell lysates were collected every 15 or 5 min after the initial 30 min of release, respectively.

#### Metaphase synchronization

To synchronize RPE1 cells at metaphase, cells were seeded and synchronized at G_1_/S by Palbociclib (150 nM) for 24 h. The cells were then washed 3x with fresh, warm media. At 10–11 h post-release, cells were treated with Eg5i (2 μM) for 4 h. The cells were subsequently washed 3x with fresh, warm media and allowed to grow for 30 min before treating them with Apicn (200 μM) and PROtame (24 μM) for 90 min. For immunofluorescence, the cells were washed 3x with fresh, warm media, and coverslips were collected every 90 s. For western blot analysis, arrested mitotic cells were collected by firmly tapping the plates to dislodge rounded cells. The shaken-off cells were collected and washed 3x with fresh, warm media before re-culturing them on a 10-cm plate. Cell lysates were collected every 3 min.

### FACS analysis

For cell cycle analysis, cells were collected and analyzed as described in Trotter and Hagan, 2020. Briefly, cells were harvested using trypsin and washed with PBS. Cells were then fixed in ice-cold 70% Ethanol and stored at −20 °C for up to two weeks. For staining, fixed cells were washed with PBS, treated with 100 μg/ml RNase, and stained with 50 μg/ml Propidium Iodide (PI). Samples were analyzed on a BD LSR II flow cytometer using FACS-Diva™ software within 30 min of PI addition. Data analysis was performed using FlowJo software.

### RNA extraction and quantitative real-time PCR (RT-qPCR)

Total RNA was extracted from collected cells using TRIzol™ Reagent according to the manufacturer’s protocol. The RNA was reverse transcribed into cDNA using the high-capacity RNA-to-cDNA™ Kit.

Quantitative real-time PCR (RT-qPCR) was performed to determine the gene expression of IL-6. The reaction was carried out using Fast SYBR™ Green Master Mix with specific primers for both IL-6 (5’-GTGCCAGACTCTGGACAA-3’ and 5’-AGACAGCCACTCACCTCTTCAG-3’) and the housekeeping gene HPRT (hypoxanthine-guanine phosphoribosyltransferase; 5’- ATCAGACTGAAGAGCTATTGTAATGA-3’ and 5’- TGGCTTATATCCAACACTTCGTG-3’). Gene expression was quantified by measuring the cycle threshold (Ct) value using QuantStudio™ Design & Analysis Software, and relative expression levels were determined by normalizing to HPRT using the ΔΔCt method.

### In vitro kinase assay

3xFLAG PKMYT1 was induced in HeLa-FRT and RPE-FRT cells overnight using doxycycline (1 μg/ml). Prometaphase cells were isolated as described in the cell synchronization section. Cells were lysed using ELB buffer (50 mM HEPES, 250 mM sodium chloride, 0.1% IGEPAL630, 1x protease inhibitor, 1x PhosSTOP, 1 mM DTT and 10 μM RO-3306). Lysates were normalized using Bio-Rad reagent, and an equal amount was applied to Pierce FLAG beads. The mixture was rotated for 1 h at 4 °C. The beads were then washed three times with ELB buffer and one time with KAB buffer (50 mM Tris-HCl, pH 7.5, 10 mM magnesium chloride, 1 mM DTT, and 10 μM RO 3306). The beads were resuspended using 49 μL of KAB buffer followed by 0.25 μg CDK1-Cyclin B1 and 25 μl 300 μM ATP, with either DMSO or RP 6306 (500 nM). The reaction was kept at room temperature for 5 min before adding 40 μL of sample buffer with 285 mM DTT. The reaction was heated for 10 min at 70 °C. The beads were removed before separating the supernatant on a 12% SDS-PAGE gel.

### Western blotting

For Western blotting experiments, cells were lysed (except for the in vitro kinase assay experiment) for 30 min at 4 °C in NEB Lysis Buffer containing 1X cOmplete protease inhibitor, 1X PhosSTOP phosphatase inhibitor, and 1 mM PMSF. After determining protein concentrations with the Bio-Rad reagent, equal amounts of protein were separated by SDS-PAGE on NuPAGE 12% Bis-Tris gels. Proteins were then transferred to either a PVDF membrane or a nitrocellulose membrane (for the anti-p-T-P antibody). Following transfer, membranes were blocked for 1 h at room temperature. A 5% bovine serum albumin (BSA) in PBS-T solution was used for the anti-Wee1 and pY15 Cdc2 antibodies, while for the other antibodies, membranes were blocked with a 5% milk solution in TBS-T. Membranes were incubated with primary antibodies with the following concentrations: anti-PKMYT1 (1 in 250), anti-Wee1, anti-CDK Y15p and anti-PP1 T320p (1 in 1000), anti-INCENP T59p (1 in 8000), anti-CDK1 (1 in 5000), anti-p-T-P (1 in 5000) and GAPDH and Vinculin (1 in 10,000) overnight at 4 °C. Following this, membranes were incubated with appropriate HRP-coupled secondary antibodies (anti-rabbit, 1:10,000; anti-mouse, 1:10,000). The signal was detected using AP buffer (100 m M NaCl, 100 mM Tris (pH 9.5), 5 mM MgCl_2_), with 6.6% 4-nitro blue tetrazolium chloride (NBT) (50 mg/ml in 70% dimethylformamide (DMF)) and 3.3% 5-bromo-4-chloro-3-indolyl-phosphate disodium (BCIP) salt (50 mg/ml in 30% DMF). Development was stopped by transferring the membranes to a container of ice water with EDTA. Membranes were allowed to dry before imaging on the Bio-Rad Chemidoc.

### Immunofluorescence imaging

Cells grown on 0.01% Poly-L-lysine-coated coverslips (18 mm × 18 mm) were fixed with 3.7% paraformaldehyde for 10 min, followed by permeabilization with 0.2% Triton X-100 for another 10 min, with PBS washes between each step. Coverslips were then blocked for 1 h with 1% BSA in PBS-T (PBS with 0.05% Tween). Primary antibodies were diluted 1:500 in blocking buffer and incubated overnight at 4 °C. After washing, secondary antibodies (1:1000) and 2 mg/ml DAPI were applied in blocking buffer for 1 h at room temperature. Finally, coverslips were mounted using ProLong Diamond Antifade Mounting media and visualized with a Zeiss Confocal Microscope. Images were processed with ImageJ.

For experiments assessing DNA damage markers—including γH2A.X foci, micronucleation, and chromatin bridges—cells were grown in 24-well Sensoplates. Staining was performed according to the immunofluorescence protocol described above. Plates were imaged using a PerkinElmer Opera Phenix High-Content Screening System, and data were analyzed using the associated Harmony software or ImageJ.

Blinding was not used during data collection or analysis; however, automated analysis was employed where possible to ensure objectivity.

### Live-cell imaging

Cells were seeded in a 24-well Sensoplate for live-cell imaging. The media was FluoroBrite DMEM (Gibco) supplemented with 10% FBS, 1% Penicillin, 1% Sodium Pyruvate and 1% Streptomycin. Where indicated, cells were stained with 70 nM siR-DNA following the manufacturer’s protocol. Imaging was performed on a PerkinElmer Opera Phenix High-Content Screening System. Prior to imaging, the microscope chamber was pre-equilibrated for at least 4 h at 37 °C and 5% CO_2_. All image acquisitions utilized a 647 nm laser at 25% power with a 50 ms exposure time. For the chromosome segregation errors experiment, time-lapse images were acquired every 4–7 min for 60 min, taking nine individual Z-stacks (Step size = 1 μm) at each interval, which were subsequently processed by maximum intensity projection over the Z-axis.

For single-cell kinetic assays, imaging parameters remained identical, with acquisition intervals adjusted based on the experiment: 10–15 min for 9–11 h to monitor SAC slippage, and 1.5–2 min for 50 min to determine the duration from prometaphase to anaphase. To evaluate the acute effects of inhibitors during these assays, a brief break of less than 60 s was introduced to exchange the media with prewarmed media containing the indicated treatments.

## Supplementary information

Appendix

Peer Review File

Source data Fig. 1

Source data Fig. 2

Source data Fig. 3

Source data Fig. 4

Source data Fig. 5

Expanded View Figures

## Data Availability

This study includes no data deposited in external repositories. The source data of this paper are collected in the following database record: biostudies:S-SCDT-10_1038-S44319-026-00809-1.

## References

[CR1] Afonso O, Castellani CM, Cheeseman LP, Ferreira JG, Orr B, Ferreira LT, Bicho T, Maiato H (2019) Spatiotemporal control of mitotic exit during anaphase by an Aurora B-CDK1 crosstalk. eLife 8:e47646 31424385 10.7554/eLife.47646 PMC6706241

[CR2] Afonso O, Matos I, Pereira AJ, Aguiar P, Lampson MA, Maiato H (2014) Feedback control of chromosome separation by a midzone Aurora B gradient. Science 345:332–336 24925910 10.1126/science.1251121 PMC5240038

[CR3] Agudelo D, Duringer A, Bozoyan L, Huard CC, Carter S, Loehr J, Synodinou D, Drouin M, Salsman J, Dellaire G et al (2017) Marker-free coselection for CRISPR-driven genome editing in human cells. Nat Methods 14:615–620 28417998 10.1038/nmeth.4265

[CR4] Aiba Y, Kim J, Imamura A, Okumoto K, Nakajo N (2022) Regulation of Myt1 kinase activity via its N-terminal region in Xenopus meiosis and mitosis. Cells Dev 169:203754 34695617 10.1016/j.cdev.2021.203754

[CR5] Allan LA, Camacho Reis M, Ciossani G, Huis in ‘t Veld PJ, Wohlgemuth S, Kops GJ, Musacchio A, Saurin AT (2020) Cyclin B1 scaffolds MAD 1 at the kinetochore corona to activate the mitotic checkpoint. EMBO J 39:e103180 32202322 10.15252/embj.2019103180 PMC7298293

[CR6] Baldin V, Ducommun B (1995) Subcellular localisation of human wee1 kinase is regulated during the cell cycle. J Cell Sci 108:2425–2432 7673359 10.1242/jcs.108.6.2425

[CR7] Ciccia A, Elledge SJ (2010) The DNA damage response: making it safe to play with knives. Mol Cell 40:179–204 20965415 10.1016/j.molcel.2010.09.019 PMC2988877

[CR8] Cirillo L, Young R, Veerapathiran S, Roberti A, Martin M, Abubacar A, Tuck EC, Pines J (2024) Spatial control of the APC/C ensures the rapid degradation of cyclin B1. EMBO J 43:4324–4355 39143240 10.1038/s44318-024-00194-2 PMC11445581

[CR9] Clute P, Pines J (1999) Temporal and spatial control of cyclin B1 destruction in metaphase. Nat Cell Biol 1:82–87 10559878 10.1038/10049

[CR10] Cooke CA, Heck MM, Earnshaw WC (1987) The inner centromere protein (INCENP) antigens: movement from inner centromere to midbody during mitosis. J Cell Biol 105:2053–2067 3316246 10.1083/jcb.105.5.2053 PMC2114862

[CR11] D’Angiolella V, Mari C, Nocera D, Rametti L, Grieco D (2003) The spindle checkpoint requires cyclin-dependent kinase activity. Genes Dev 17:2520–2525 14561775 10.1101/gad.267603 PMC218146

[CR12] Dorée M, Hunt T (2002) From Cdc2 to CDK1: when did the cell cycle kinase join its cyclin partner? J Cell Sci 115:2461–2464 12045216 10.1242/jcs.115.12.2461

[CR13] Evans T, Rosenthal ET, Youngblom J, Distel D, Hunt T (1983) Cyclin: a protein specified by maternal mRNA in sea urchin eggs that is destroyed at each cleavage division. Cell 33:389–396 6134587 10.1016/0092-8674(83)90420-8

[CR14] Fang G, Yu H, Kirschner MW (1998) The checkpoint protein MAD2 and the mitotic regulator CDC20 form a ternary complex with the anaphase-promoting complex to control anaphase initiation. Genes Dev 12:1871–1883 9637688 10.1101/gad.12.12.1871 PMC316912

[CR15] Fujimitsu K, Grimaldi M, Yamano H (2016) Cyclin-dependent kinase 1–dependent activation of APC/C ubiquitin ligase. Science 352:1121–1124 27103671 10.1126/science.aad3925

[CR16] Fuller BG, Lampson MA, Foley EA, Rosasco-Nitcher S, Le KV, Tobelmann P, Brautigan DL, Stukenberg PT, Kapoor TM (2008) Midzone activation of aurora B in anaphase produces an intracellular phosphorylation gradient. Nature 453:1132–1136 18463638 10.1038/nature06923 PMC2724008

[CR17] Gallo D, Young JT, Fourtounis J, Martino G, Álvarez-Quilón A, Bernier C, Duffy NM, Papp R, Roulston A, Stocco R et al (2022) CCNE1 amplification is synthetic lethal with PKMYT1 kinase inhibition. Nature 604:749–756 35444283 10.1038/s41586-022-04638-9 PMC9046089

[CR18] Gavet O, Pines J (2010) Progressive activation of CyclinB1-CDK1 coordinates entry to mitosis. Dev Cell 18:533–543 20412769 10.1016/j.devcel.2010.02.013 PMC3325599

[CR19] Goto H, Kiyono T, Tomono Y, Kawajiri A, Urano T, Furukawa K, Nigg EA, Inagaki M (2006) Complex formation of Plk1 and INCENP required for metaphase–anaphase transition. Nat Cell Biol 8:180–187 16378098 10.1038/ncb1350

[CR20] Hayward D, Alfonso-Pérez T, Cundell MJ, Hopkins M, Holder J, Bancroft J, Hutter LH, Novak B, Barr FA, Gruneberg U (2019) CDK1-CCNB1 creates a spindle checkpoint–permissive state by enabling MPS1 kinetochore localization. J Cell Biol 218:1182–1199 30674582 10.1083/jcb.201808014 PMC6446832

[CR21] Heroes E, Lesage B, Görnemann J, Beullens M, Van Meervelt L, Bollen M (2013) The PP1 binding code: a molecular-lego strategy that governs specificity. FEBS J 280:584–595 22360570 10.1111/j.1742-4658.2012.08547.x

[CR22] Hwang LH, Lau LF, Smith DL, Mistrot CA, Hardwick KG, Hwang ES, Amon A, Murray AW (1998) Budding yeast Cdc20: a target of the spindle checkpoint. Science 279:1041–1044 9461437 10.1126/science.279.5353.1041

[CR23] Ikui AE, Yang CPH, Matsumoto T, Horwitz SB (2005) Low concentrations of taxol cause mitotic delay followed by premature dissociation of p55CDC from Mad2 and BubR1 and abrogation of the spindle checkpoint, leading to aneuploidy. Cell Cycle 4:1385–1388 16138009 10.4161/cc.4.10.2061

[CR24] Izawa D, Pines J (2015) The mitotic checkpoint complex binds a second CDC20 to inhibit active APC/C. Nature 517:631–634 25383541 10.1038/nature13911 PMC4312099

[CR25] Jackman M, Marcozzi C, Barbiero M, Pardo M, Yu L, Tyson AL, Choudhary JS, Pines J (2020) Cyclin B1-CDK1 facilitates MAD1 release from the nuclear pore to ensure a robust spindle checkpoint. J Cell Biol 219:e201907082 32236513 10.1083/jcb.201907082 PMC7265330

[CR26] Joo YK, Parrado CR, Li W, Yang R, Black E, Bleichert F, Liu Y, Kabeche L (2025) The mitotic ATR-Chk1 pathway promotes CDK1 activity for faithful chromosome segregation. Cell Rep 44:116019 40705605 10.1016/j.celrep.2025.116019 PMC12451630

[CR27] King RW, Deshaies RJ, Peters JM, Kirschner MW (1996) How proteolysis drives the cell cycle. Science 274:1652–1659 8939846 10.1126/science.274.5293.1652

[CR28] Kraft C, Herzog F, Gieffers C, Mechtler K, Hagting A, Pines J, Peters JM (2003) Mitotic regulation of the human anaphase-promoting complex by phosphorylation. EMBO J 22:6598–6609 14657031 10.1093/emboj/cdg627 PMC291822

[CR29] Kruse T, Zhang G, Larsen MSY, Lischetti T, Streicher W, Kragh Nielsen T, Bjørn SP, Nilsson J (2013) Direct binding between BubR1 and B56–PP2A phosphatase complexes regulate mitotic progression. J Cell Sci 126:1086–1092 23345399 10.1242/jcs.122481

[CR30] Lee MG, Nurse P (1987) Complementation used to clone a human homologue of the fission yeast cell cycle control gene cdc2. Nature 327:31–35 3553962 10.1038/327031a0

[CR31] Li M, Lulla AR, Wang Y, Tsavaschidis S, Wang F, Karakas C, Nguyen TD, Bui TN, Pina MA, Chen MK et al (2024) Low–molecular weight cyclin E confers a vulnerability to PKMYT1 inhibition in triple-negative breast cancer. Cancer Res 84:3864–3880 39186665 10.1158/0008-5472.CAN-23-4130 PMC11567801

[CR32] Lianga N, Williams EC, Kennedy EK, Doré C, Pilon S, Girard SL, Deneault J, Rudner AD (2013) A WEE1 checkpoint inhibits anaphase onset. J Cell Biol 201:843–862 23751495 10.1083/jcb.201212038 PMC3678162

[CR33] Liu F, Stanton JJ, Wu Z, Piwnica-Worms H (1997) The human Myt1 kinase preferentially phosphorylates Cdc2 on threonine 14 and localizes to the endoplasmic reticulum and Golgi complex. Mol Cell Biol 17:571–583 9001210 10.1128/mcb.17.2.571 PMC231782

[CR34] Maiato H, Afonso O, Matos I (2015) A chromosome separation checkpoint: a midzone Aurora B gradient mediates a chromosome separation checkpoint that regulates the anaphase-telophase transition. Bioessays 37:257–266 25470791 10.1002/bies.201400140

[CR35] McAinsh AD, Kops GJ (2023) Principles and dynamics of spindle assembly checkpoint signalling. Nat Rev Mol Cell Biol 24:543–559 36964313 10.1038/s41580-023-00593-z

[CR36] Morrison KR, Wang T, Chan KY, Trotter EW, Gillespie A, Michael MZ, Oakhill JS, Hagan IM, Petersen J (2023) Elevated basal AMP-activated protein kinase activity sensitizes colorectal cancer cells to growth inhibition by metformin. Open Biol 13:230021 37042113 10.1098/rsob.230021 PMC10090877

[CR37] Mueller PR, Coleman TR, Kumagai A, Dunphy WG (1995) Myt1: a membrane-associated inhibitory kinase that phosphorylates Cdc2 on both threonine-14 and tyrosine-15. Science 270:86–90 7569953 10.1126/science.270.5233.86

[CR38] Murray AW, Kirschner MW (1989) Cyclin synthesis drives the early embryonic cell cycle. Nature 339:275–280 2566917 10.1038/339275a0

[CR39] Murray AW, Solomon MJ, Kirschner MW (1989) The role of cyclin synthesis and degradation in the control of maturation promoting factor activity. Nature 339:280–286 2566918 10.1038/339280a0

[CR40] Musacchio A, Salmon ED (2007) The spindle-assembly checkpoint in space and time. Nat Rev Mol Cell Biol 8:379–393 17426725 10.1038/nrm2163

[CR41] Nakajima H, Toyoshima-Morimoto F, Taniguchi E, Nishida E (2003) Identification of a consensus motif for Plk (Polo-like kinase) phosphorylation reveals Myt1 as a Plk1 substrate. J Biol Chem 278:25277–25280 12738781 10.1074/jbc.C300126200

[CR42] Nakanishi M, Ando H, Watanabe N, Kitamura K, Ito K, Okayama H, Miyamoto T, Agui T, Sasaki M (2000) Identification and characterization of human WEE1B, a new member of the WEE1 family of CDK-inhibitory kinases. Genes Cells 5:839–847 11029659 10.1046/j.1365-2443.2000.00367.x

[CR43] Nezi L, Musacchio A (2009) Sister chromatid tension and the spindle assembly checkpoint. Curr Opin Cell Biol 21:785–795 19846287 10.1016/j.ceb.2009.09.007

[CR44] Parker LL, Piwnica-Worms H (1992) Inactivation of the p34 cdc2-cyclin B complex by the human WEE1 tyrosine kinase. Science 257:1955–1957 1384126 10.1126/science.1384126

[CR45] Peters JM (2002) The anaphase-promoting complex: proteolysis in mitosis and beyond. Mol Cell 9:931–943 12049731 10.1016/s1097-2765(02)00540-3

[CR46] Qi W, Tang Z, Yu H (2006) Phosphorylation-and polo-box–dependent binding of Plk1 to Bub1 is required for the kinetochore localization of Plk1. Mol Biol Cell 17:3705–3716 16760428 10.1091/mbc.E06-03-0240 PMC1525235

[CR61] Santaguida S, Vernieri C, Villa F, Ciliberto A, Musacchio A (2011) Evidence that Aurora B is implicated in spindle checkpoint signalling independently of error correction. EMBO J 30:1508 10.1038/emboj.2011.70 PMC3102279 21407176

[CR47] Schmidt M, Rohe A, Platzer C, Najjar A, Erdmann F, Sippl W (2017) Regulation of G2/M transition by inhibition of WEE1 and PKMYT1 kinases. Molecules 22:2045 29168755 10.3390/molecules22122045 PMC6149964

[CR48] Shepperd LA, Meadows JC, Sochaj AM, Lancaster TC, Zou J, Buttrick GJ, Rappsilber J, Hardwick KG, Millar JB (2012) Phosphodependent recruitment of Bub1 and Bub3 to Spc7/KNL1 by Mph1 kinase maintains the spindle checkpoint. Curr Biol 22:891–899 22521786 10.1016/j.cub.2012.03.051 PMC3780767

[CR49] Song C, Zhang M, Kruse T, Møller MH, López-Méndez B, Zhang Y, Zhai Y, Wang Y, Lei T, Kettenbach AN et al (2024) Self-priming of Plk1 binding to BubR1 ensures accurate mitotic progression. Commun Biol 7:1473 39516273 10.1038/s42003-024-07205-2 PMC11549336

[CR50] Tanaka TU, Rachidi N, Janke C, Pereira G, Galova M, Schiebel E, Stark MJ, Nasmyth K (2002) Evidence that the Ipl1-Sli15 (Aurora kinase-INCENP) complex promotes chromosome bi-orientation by altering kinetochore-spindle pole connections. Cell 108:317–329 11853667 10.1016/s0092-8674(02)00633-5

[CR51] Thuriaux P, Nurse P, Carter B (1978) Mutants altered in the control co-ordinating cell division with cell growth in the fission yeast *Schizosaccharomyces pombe*. Mol Gen Genet 161:215–220 672898 10.1007/BF00274190

[CR52] Vader G, Kauw JJ, Medema RH, Lens SM (2006) Survivin mediates targeting of the chromosomal passenger complex to the centromere and midbody. EMBO Rep 7:85–92 16239925 10.1038/sj.embor.7400562 PMC1369225

[CR53] Vanoosthuyse V, Hardwick KG (2003) The Complexity of Bub1 regulation: phosphorylation, phosphorylation, phosphorylation. Cell Cycle 2:118–119 12695661 10.4161/cc.2.2.343

[CR54] Visconti R, Della Monica R, Palazzo L, D’Alessio F, Raia M, Improta S, Villa MR, Del Vecchio L, Grieco D (2015) The Fcp1-Wee1-Cdk1 axis affects spindle assembly checkpoint robustness and sensitivity to antimicrotubule cancer drugs. Cell Death Differ 22:1551–1560 25744022 10.1038/cdd.2015.13 PMC4532778

[CR55] Watanabe N, Arai H, Nishihara Y, Taniguchi M, Watanabe N, Hunter T, Osada H (2004) M-phase kinases induce phospho-dependent ubiquitination of somatic WEE1 by SCFβ-TrCP. Proc Natl Acad Sci USA 101:4419–4424 15070733 10.1073/pnas.0307700101 PMC384762

[CR56] Watanabe N, Broome M, Hunter T (1995) Regulation of the human WEE1Hu CDK tyrosine 15-kinase during the cell cycle. EMBO J 14:1878–1891 7743995 10.1002/j.1460-2075.1995.tb07180.x PMC398287

[CR62] Woods A, Sherwin T, Sasse R, MacRae TH, Baines AJ, Gull K (1989) Definition of individual components within the cytoskeleton of Trypanosoma brucei by a library of monoclonal antibodies. J Cell Sci 93:491 10.1242/jcs.93.3.491 2606940

[CR57] Wolf F, Wandke C, Isenberg N, Geley S (2006) Dose-dependent effects of stable cyclin B1 on progression through mitosis in human cells. EMBO J 25:2802–2813 16724106 10.1038/sj.emboj.7601163 PMC1500859

[CR58] Yamagishi Y, Yang CH, Tanno Y, Watanabe Y (2012) MPS1/Mph1 phosphorylates the kinetochore protein KNL1/Spc7 to recruit SAC components. Nat Cell Biol 14:746–752 22660415 10.1038/ncb2515

[CR59] Yamaguchi S, Decottignies A, Nurse P (2003) Function of Cdc2p-dependent Bub1p phosphorylation and Bub1p kinase activity in the mitotic and meiotic spindle checkpoint. EMBO J 22:5077–5088 10.1093/emboj/cdg100 PMC150333 12606573

[CR60] Yesbolatova A, Saito Y, Kitamoto N, Makino-Itou H, Ajima R, Nakano R, Nakaoka H, Fukui K, Gamo K, Tominari Y et al (2020) The auxin-inducible degron 2 technology provides sharp degradation control in yeast, mammalian cells, and mice. Nat Commun 11:5701 33177522 10.1038/s41467-020-19532-z PMC7659001

