# Supplementary material for: PKMYT1 has an important role in the timing and fidelity of chromosome segregation
Source: EMBO Rep. 2026 Jun 5;27(13):3564–84. doi: 10.1038/s44319-026-00809-1 (PMC13354794; doi:10.1038/s44319-026-00809-1)
Supplement: Supplementary file 1 — Appendix [file 44319_2026_809_MOESM1_ESM.pdf]

Appendix for PKMYT1 has an Important Role in the Timing and Fidelity of Chromosome Segregation

Appendix Figures	Legend Title	Page Number
Appendix Figure S1	Characterization of PKMYT1 knockout clones and impact on CDK1 phosphorylation.	1
Appendix Figure S2	Inhibiting PKMYT1 significantly accelerates the G2/M to G1 transition.	2-3
Appendix Figure S3	Acute PKMYT1 inhibition triggers an accelerated prometaphase-to-anaphase transition in RPE-1 cells	5
Appendix Figure S4	Spatial Localization of INCENP and Lamin B in RPE-1 (PKMYT1 -/-)	6
Appendix Figure S5	PKMYT1 activity is required for regulated mitotic entry but not for S-phase progression.	7-8
Appendix Figure S6	Tubulin structure of cells undergoing mitotic slippage following Paclitaxel treatment.	6

Appendix Figure S 1

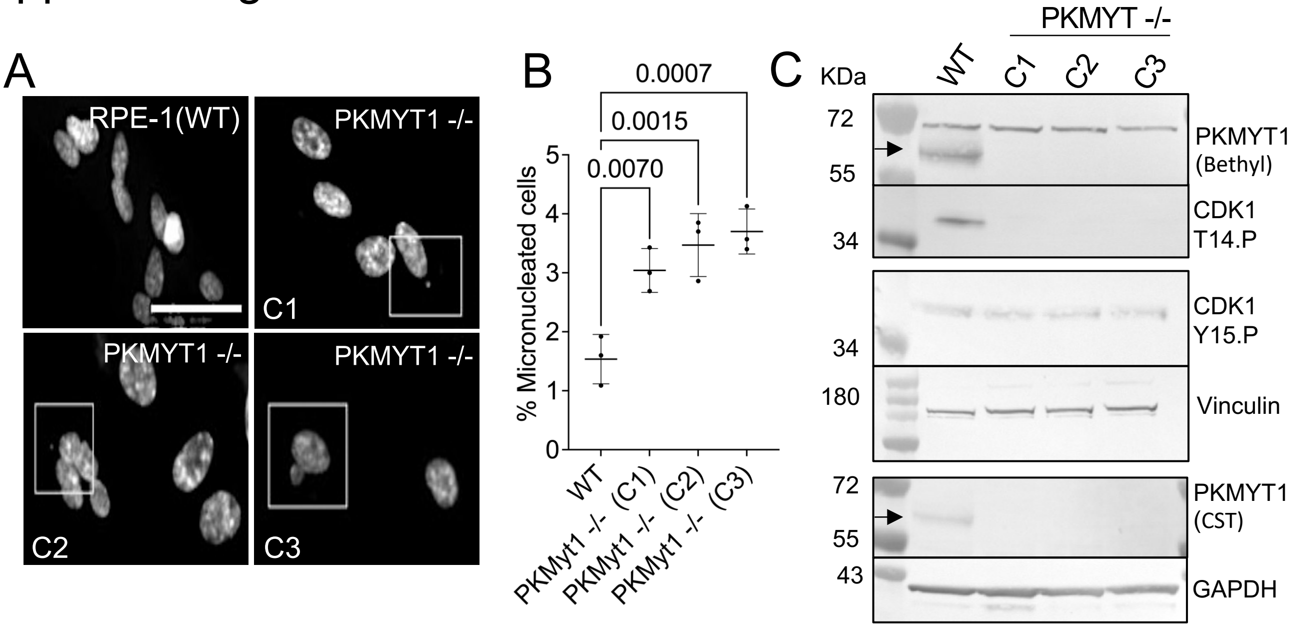

Appendix Figure S 1. Characterization of PKMYT1 knockout clones and impact on CDK1 phosphorylation.

(A) Representative fluorescence confocal images of asynchronous WT RPE1 cells or three different PKMYT1 knockout clones (PKMYT1−/− clones 1, 2, and 3). Cells were fixed and stained with DAPI to visualize DNA. Boxed regions show representative examples of micronucleation. (B) Quantification of micronucleus frequency for the cells imaged in (A). Data are presented as mean ± SD, n = 3 biological replicates. Statistical analysis was performed by one-way ANOVA. (C) Biochemical validation of PKMYT1 ablation and substrate specificity. Analysis of asynchronous lysates from WT and PKMYT1−/− RPE-1 cells. Equal protein lysates were separated by 12% SDS-PAGE. PKMYT1 (Bethyl) was detected with pT14-CDK1; PKMYT1 (CST) was detected with GAPDH; and pY15-CDK1 was detected with Vinculin. GAPDH and Vinculin served as loading controls. The use of two independent PKMYT1 antibodies confirmed complete protein loss. Scale bar, 50 μm

Appendix Figure S 2

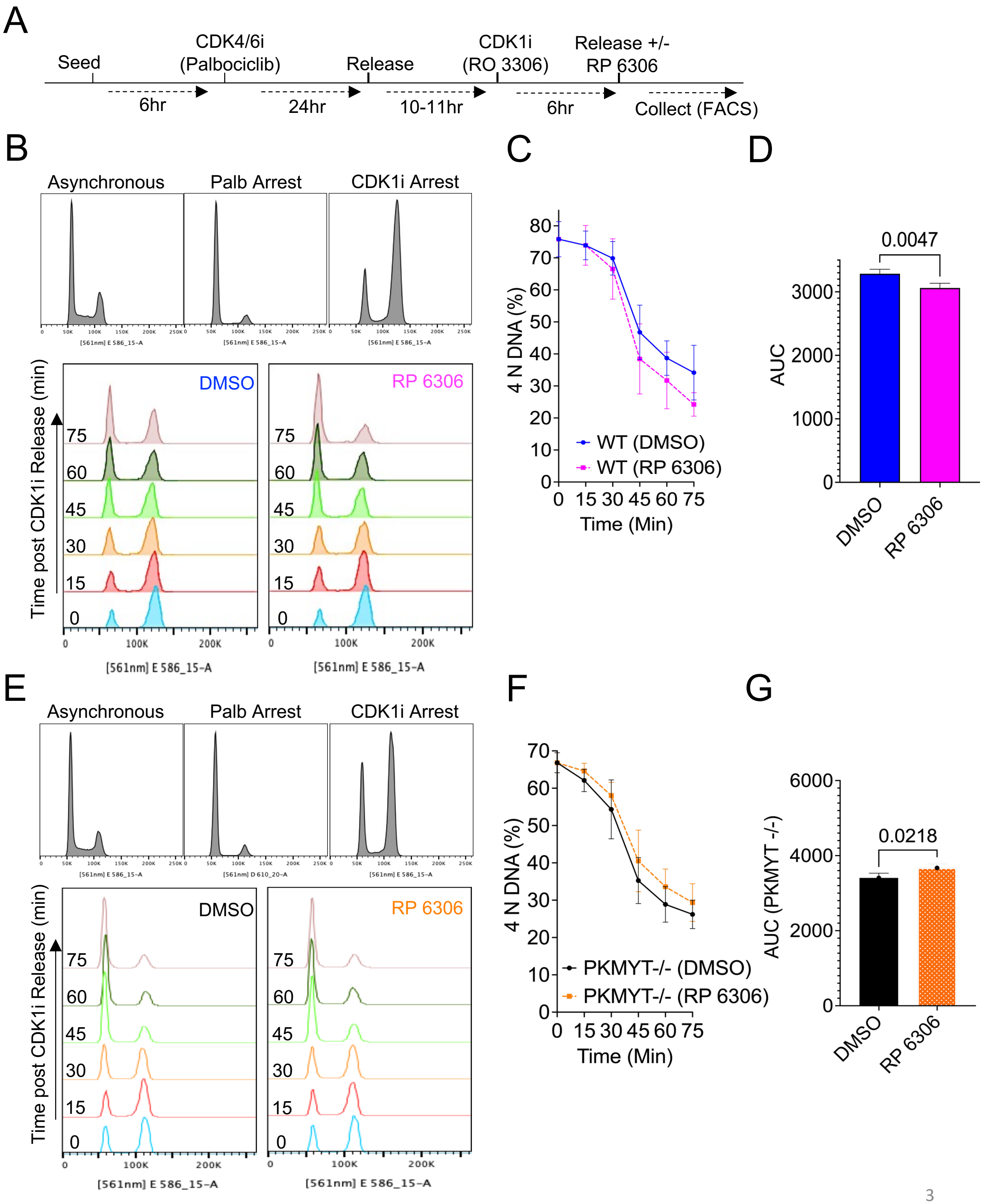

Appendix Figure S 2. Inhibiting PKMYT1 significantly accelerates the G2/M to G1 transition.

(A) Schematic representation of the refined synchronization protocol. RPE-1 cells were synchronized in G1 using the CDK4/6 inhibitor palbociclib (150 nM), followed by a sequential arrest at the G2/M boundary using the CDK1 inhibitor RO 3306 (10μM). (B, E) Representative propidium iodide (PI) flow cytometry DNA content profiles of (B) Wild-type (WT) and (E) PKMYT1−/− cells. Following release from the G2/M block, cells were treated with either DMSO or RP 6306 (500 nM) and sampled every 15 minutes. (C, F) Quantification of the percentage of the population remaining with 4N DNA content over time for (C) WT and (F) PKMYT1−/− cells. Data are presented as mean ± SD, n = 3 biological replicates. (D, G) Statistical comparison of the rate of mitotic progression for (D) WT and (G) PKMYT1−/− cells were performed on the Area Under the Curve (AUC) by Student's t-test.

Appendix Figure S 3

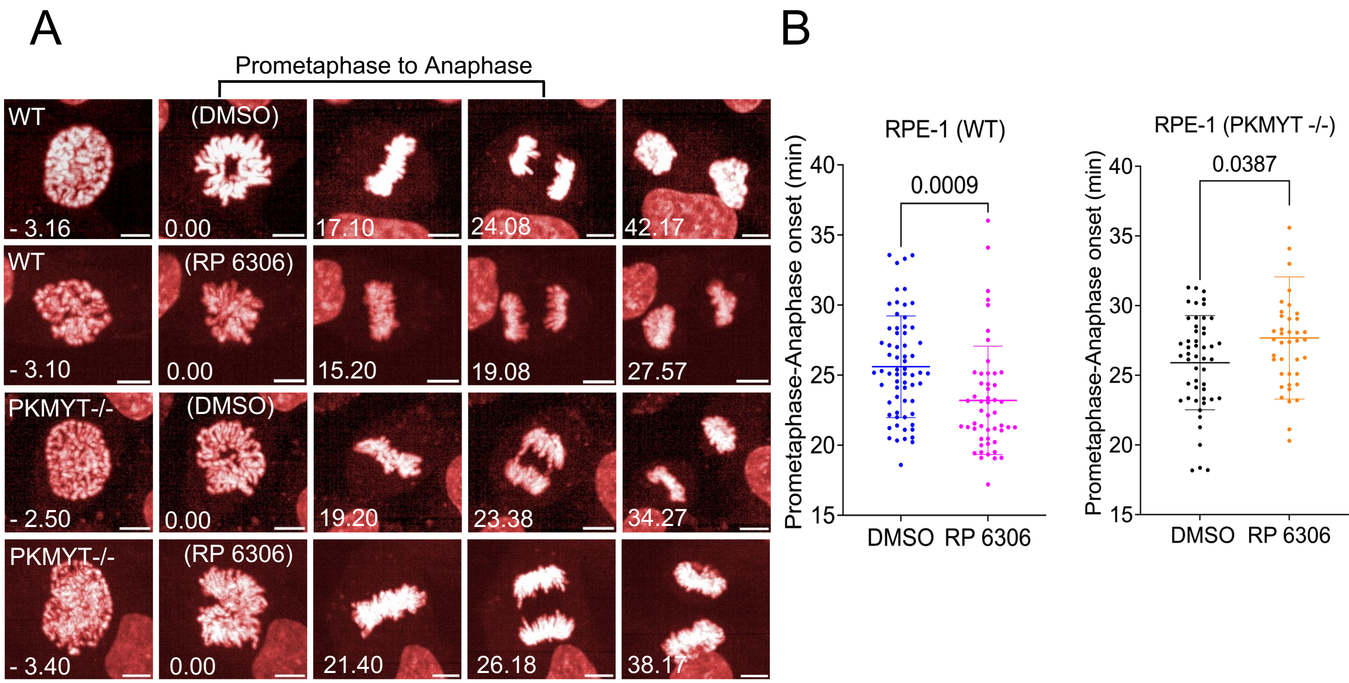

Appendix Figure S 3. Acute PKMYT1 inhibition triggers an accelerated prometaphase-to-anaphase transition in RPE-1 cells

(A) Representative maximum intensity projections of time-lapse confocal images showing RPE-1 (WT) or PKMYT1−/− cells progressing through mitosis. Chromatin was visualized using SiR-DNA. Cells were monitored for 7–8 minutes prior to a brief (<1 min) media exchange with pre-warmed medium containing either DMSO or RP 6306 (500 nM). Subsequent imaging was performed at 1.5–2 minute intervals for 50 minutes. Scale bar, 5µm. (B) Quantification of the duration from prometaphase to anaphase onset in the single-cell assay described in (A). Only cells that were in prometaphase at the time of media exchange were included in the quantification to measure the acute effect of PKMYT1 inhibition. Each data point represents an individual cell. Results for DMSO and RP-6306 represent the mean ± SD of at least 15 biological replicates, encompassing a total of >40 cells per condition. Statistical significance was determined by a Student’s t-test.

Appendix Figure S 4

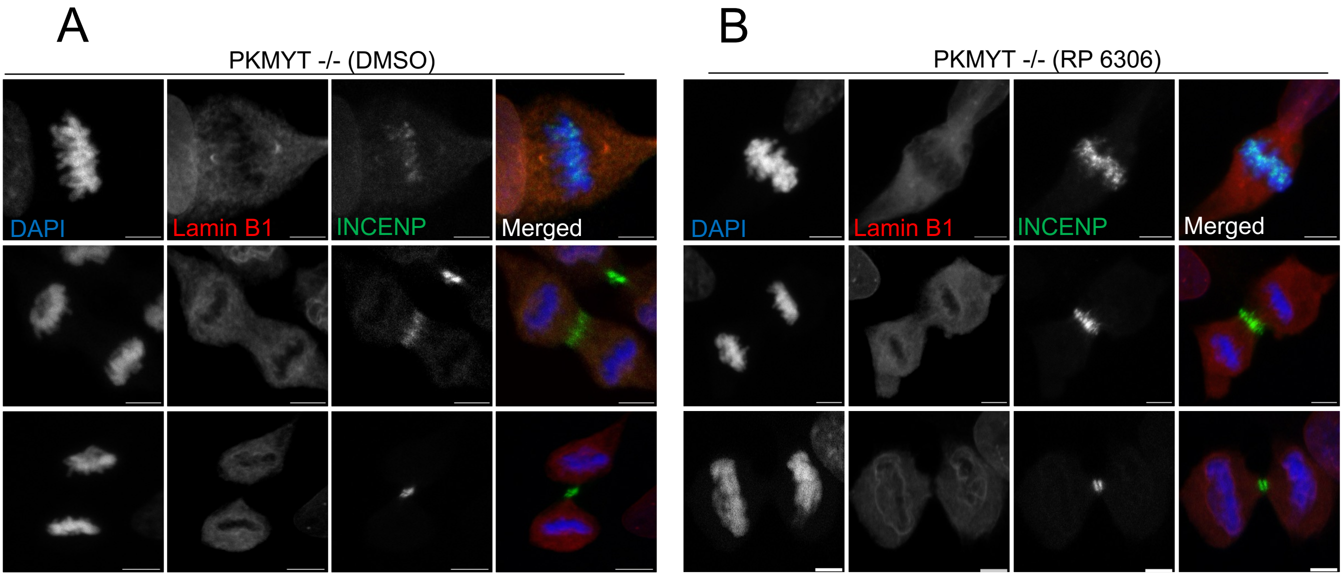

Appendix Figure S 4. Spatial Localization of INCENP and Lamin B in RPE-1 (PKMYT1 -/-)

(A, B) Representative maximum intensity projections of confocal images showing asynchronous PKMYT1−/− RPE-1 cells progressing through mitosis. Cells were treated with either (A) DMSO or (B) RP-6306 (500 nM) for 1 hour prior to fixation. "Cells were immunolabeled for Lamin B1 to visualize nuclear envelope reformation and INCENP to track chromosomal passenger complex (CPC) relocalization; DNA was counterstained with DAPI. Scale bar, 5 µm.

Appendix Figure S 5

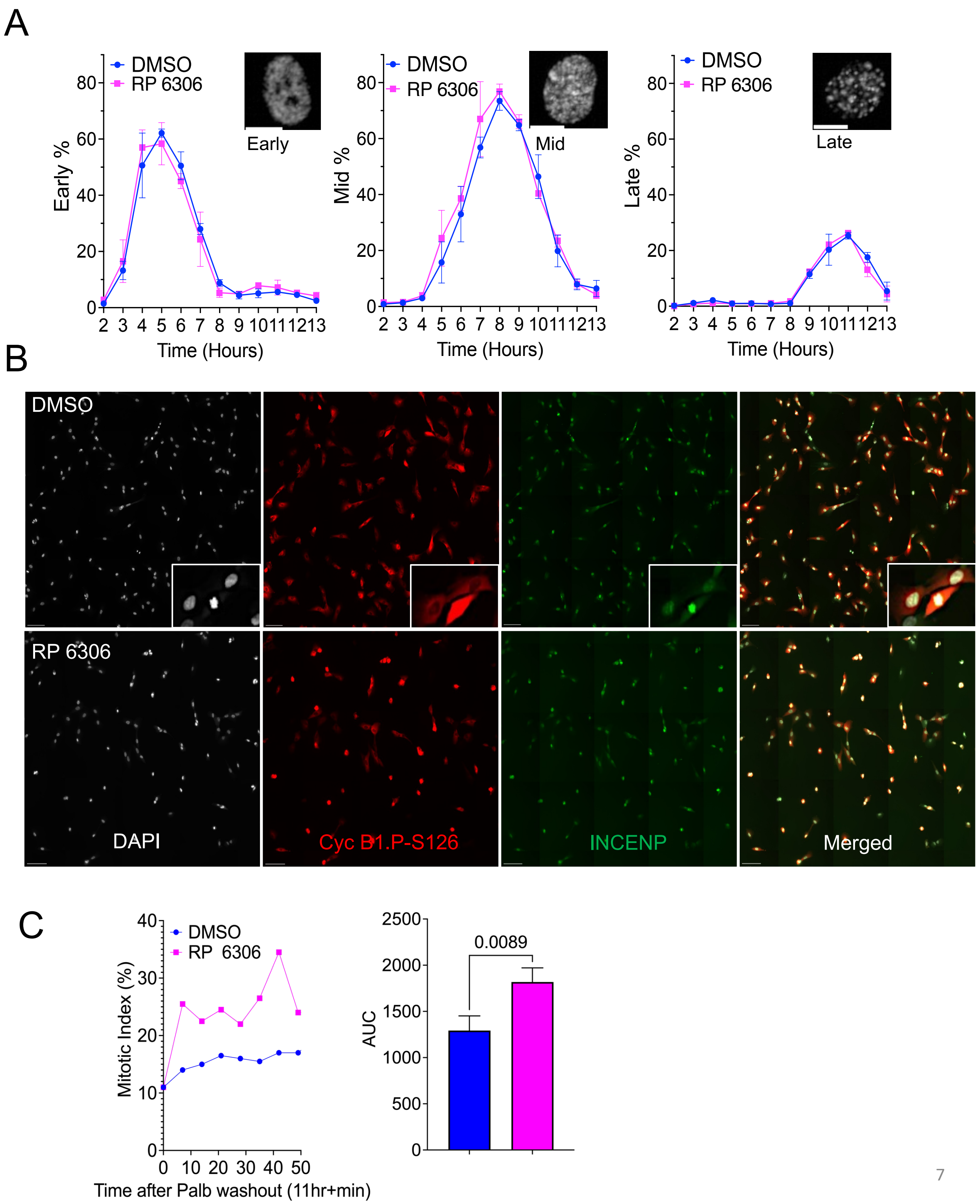

Appendix Figure S. 5. PKMYT1 activity is required for regulated mitotic entry but not for S-phase progression.

(A) Quantification of S-phase progression in RPE-1 cells. Progression through S phase was quantified in WT RPE1 cells synchronized with palbociclib (150 nM) for 24 hours. Cells were then released into fresh medium containing either DMSO or RP 6306. Cells were pulsed with EdU and fixed hourly up to 13 hours post-release for confocal imaging. The DNA staining pattern was used to define the stage of S phase (e.g., early, mid, or late). Representative images of these patterns are shown in the boxed regions. Data are presented as mean ± SEM, n = 3 biological replicates. Scale bar, 10 µm.

(B) Representative confocal microscopy images of WT RPE-1 cells. Cells were synchronized with palbociclib (150 nM) for 24 hours and released into fresh medium. At 11 hours post-release, cells were treated to either DMSO or RP 6306. Cells were fixed every 10 minutes for up to 50 minutes and imaged using confocal microscopy. Cells were stained with DAPI (DNA), phospho-Serine 126 Cyclin B1 (a mitotic marker), and INCENP. Scale bar, 100 µm. Mitotic cells were identified by nuclear translocation of phospho-Serine 126 Cyclin B1 (magnified inset). (C) Quantification of the mitotic index of RPE1 cells from the images described in (B). The statistical analysis was calculated using the Area Under the Curve (AUC) from n=2 biological experiments by a Student's t-test.

Appendix Figure S 6

A

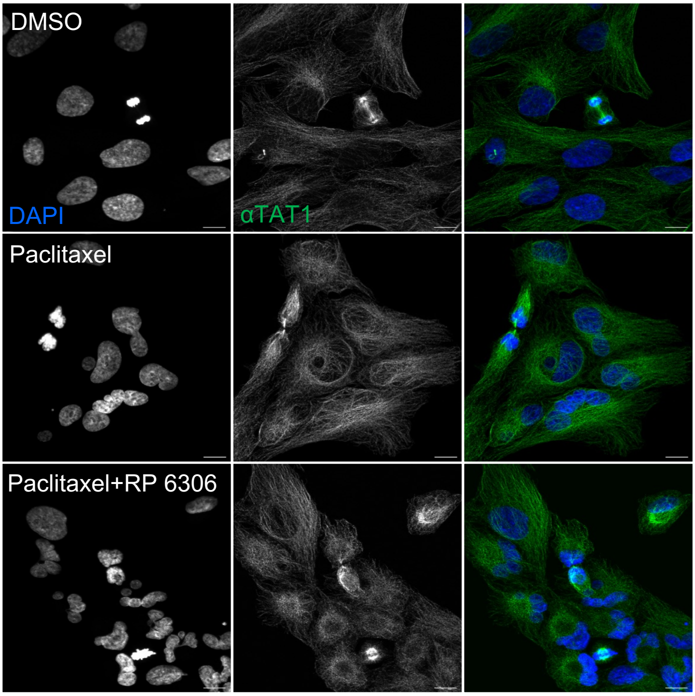

Appendix Figure S 6. Tubulin structure of cells undergoing mitotic slippage following Paclitaxel treatment.
Representative maximum intensity projections of confocal images of RPE-1 WT cells. Cells were treated with 5 nM Paclitaxel in the presence of either DMSO or RP-6306 (500 nM) for 10 hours. After treatment, cells were fixed and stained with DAPI (DNA) and α-TAT1 to visualize the microtubule network. Scale bar, 50 µm.
