# Supplementary material for: PKMYT1 has an important role in the timing and fidelity of chromosome segregation
Source: EMBO Rep. 2026 Jun 5;27(13):3564–84. doi: 10.1038/s44319-026-00809-1 (PMC13354794; doi:10.1038/s44319-026-00809-1)
Supplement: Supplementary file 3 — Source data Fig. 1 [file 44319_2026_809_MOESM3_ESM.zip › Source_Data_Figure_1/1J/README file.rtf]

Representative confocal images of RPE-1 cells (from Figure 1G) treated with DMSO. The images illustrate normal mitotic progression following release from nocodazole arrest
