# Supplementary figures and images for: PKMYT1 has an important role in the timing and fidelity of chromosome segregation
Source: EMBO Rep. 2026 Jun 5;27(13):3564–84. doi: 10.1038/s44319-026-00809-1 (PMC13354794; doi:10.1038/s44319-026-00809-1)

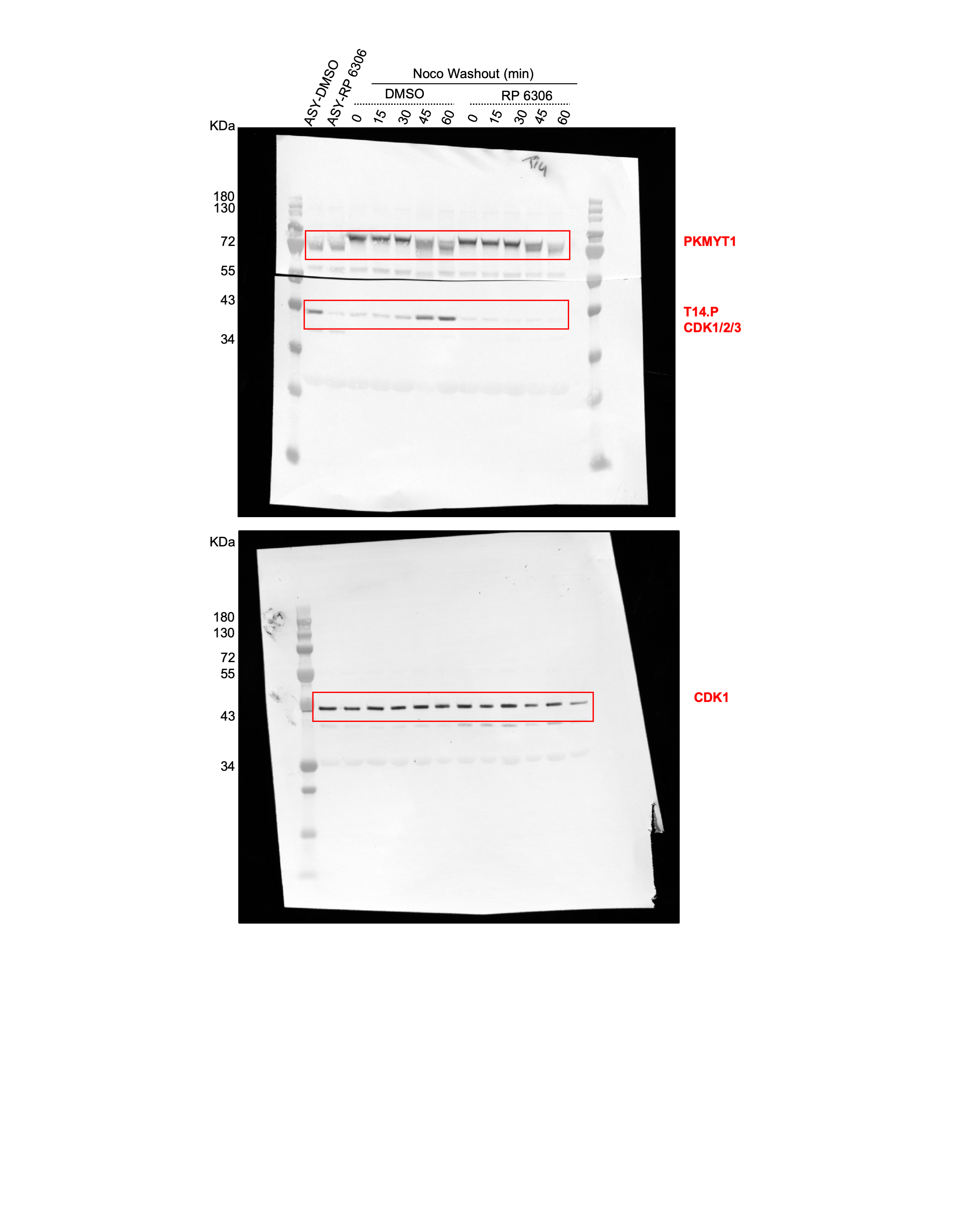

Supplement: Supplementary file 3 — Source data Fig. 1 [file 44319_2026_809_MOESM3_ESM.zip › Source_Data_Figure_1/1F/1F.tiff]

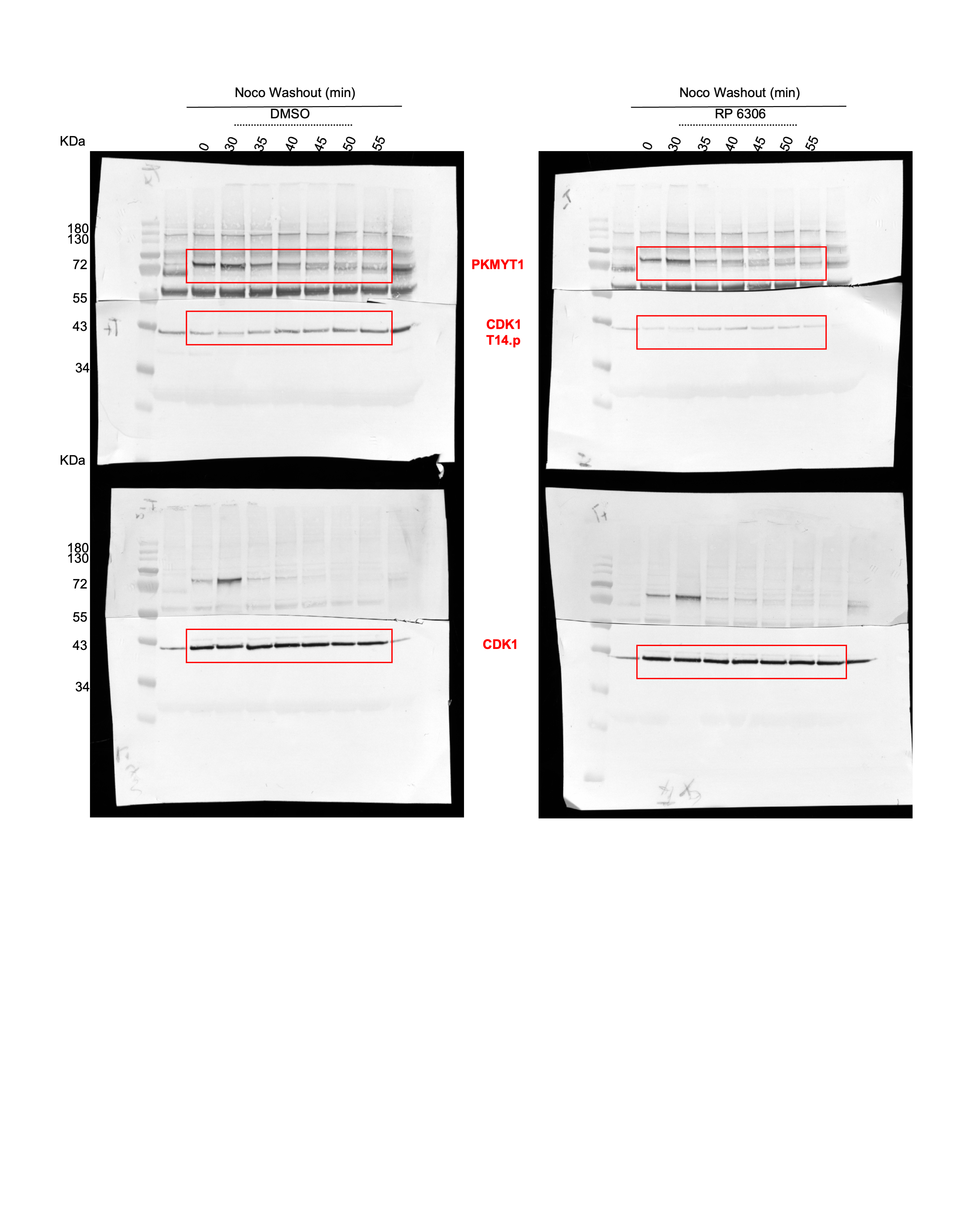

Supplement: Supplementary file 3 — Source data Fig. 1 [file 44319_2026_809_MOESM3_ESM.zip › Source_Data_Figure_1/1G/1G.tiff]

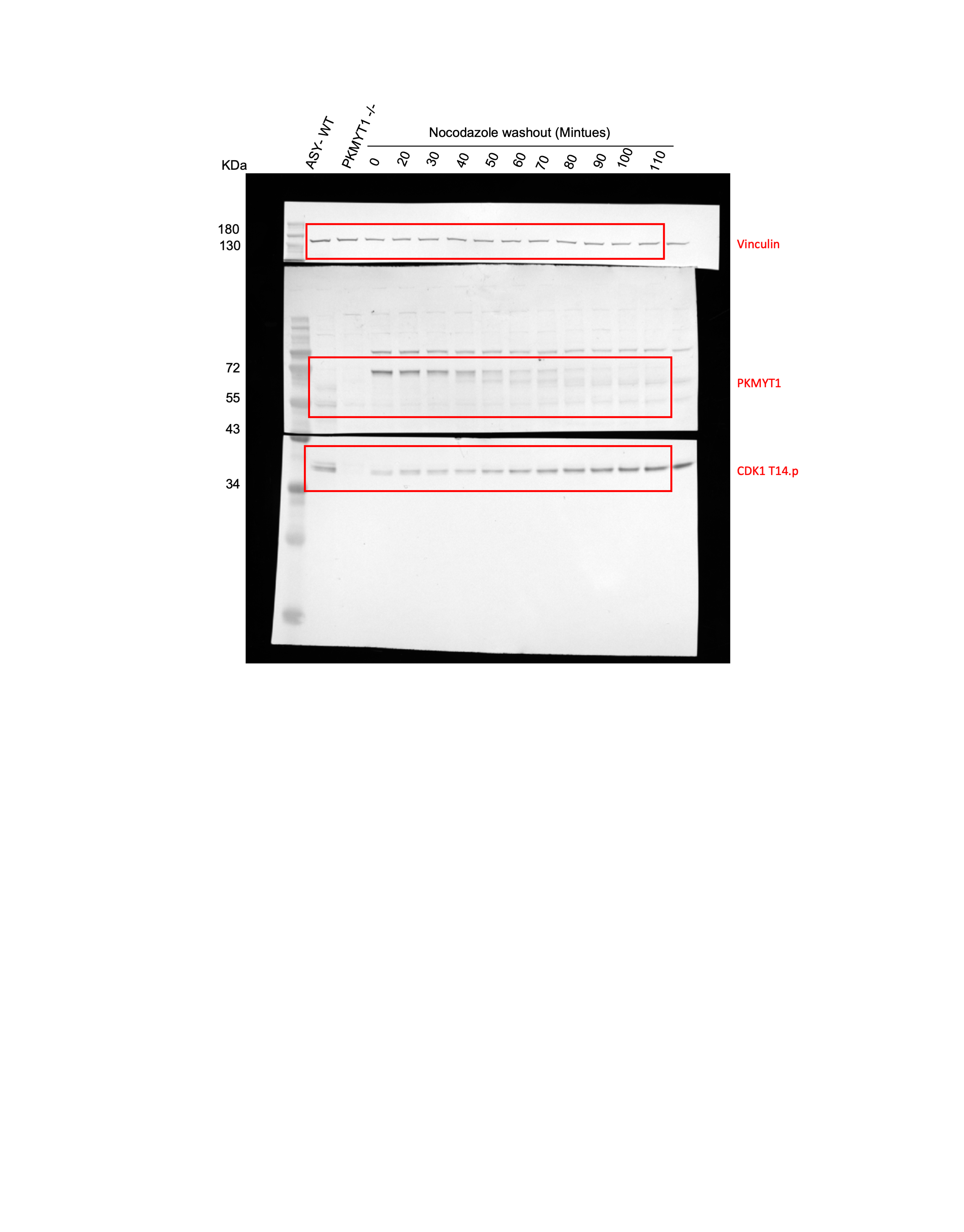

Supplement: Supplementary file 3 — Source data Fig. 1 [file 44319_2026_809_MOESM3_ESM.zip › Source_Data_Figure_1/1B/1B.tiff]

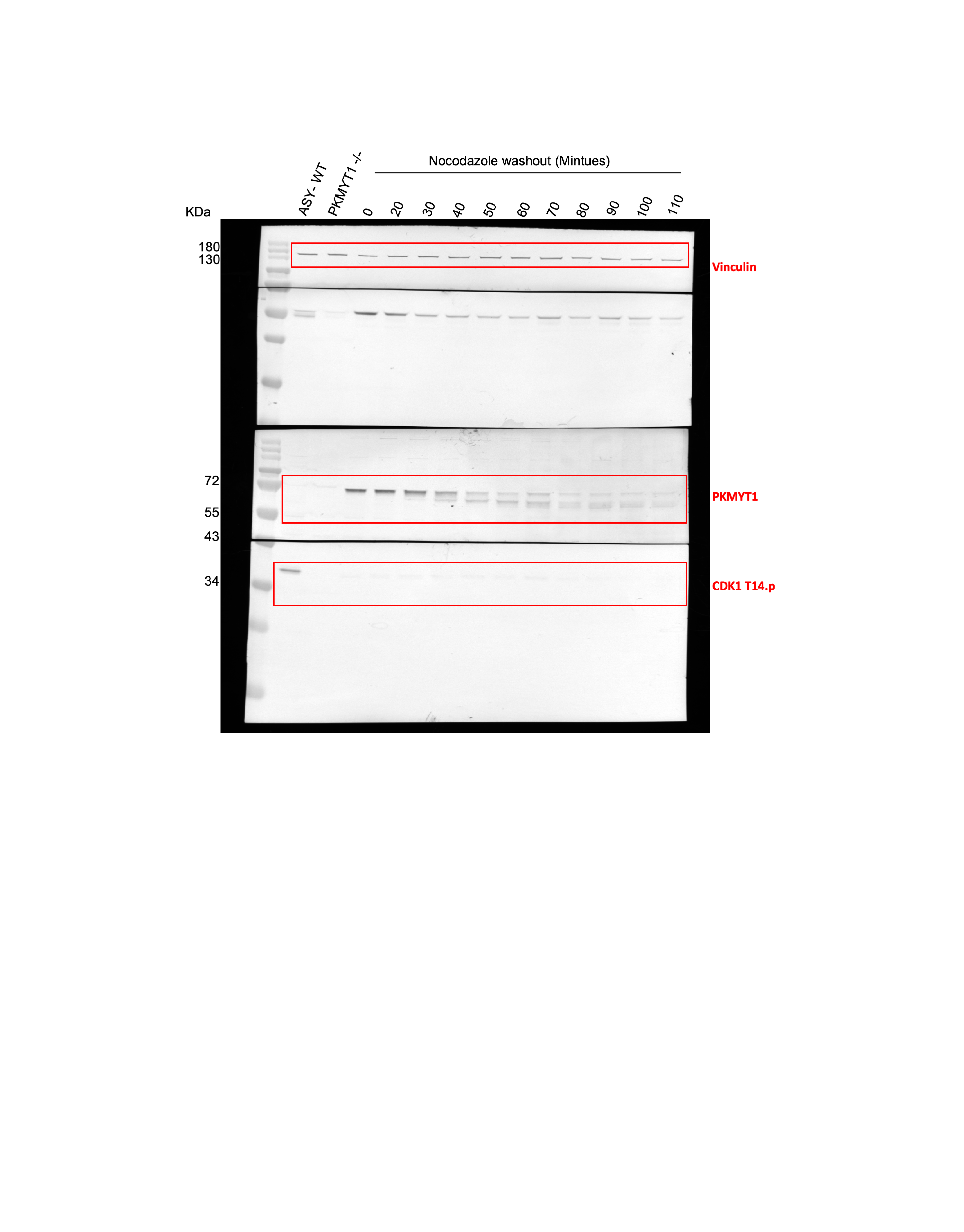

Supplement: Supplementary file 3 — Source data Fig. 1 [file 44319_2026_809_MOESM3_ESM.zip › Source_Data_Figure_1/1C/1C.tiff]

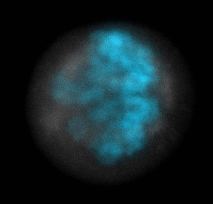

Supplement: Supplementary file 3 — Source data Fig. 1 [file 44319_2026_809_MOESM3_ESM.zip › Source_Data_Figure_1/1J/1J-0 arrrest .tif]

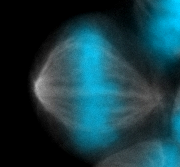

Supplement: Supplementary file 3 — Source data Fig. 1 [file 44319_2026_809_MOESM3_ESM.zip › Source_Data_Figure_1/1J/1J-35 min .tif]

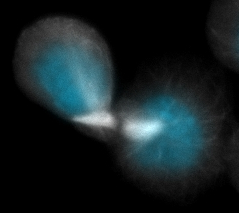

Supplement: Supplementary file 3 — Source data Fig. 1 [file 44319_2026_809_MOESM3_ESM.zip › Source_Data_Figure_1/1J/1J-55 min.tif]

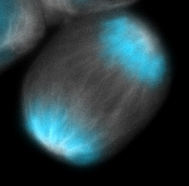

Supplement: Supplementary file 3 — Source data Fig. 1 [file 44319_2026_809_MOESM3_ESM.zip › Source_Data_Figure_1/1J/1J-45 Min.tif]

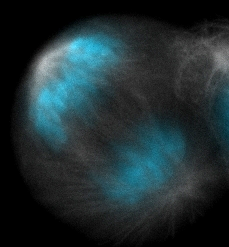

Supplement: Supplementary file 3 — Source data Fig. 1 [file 44319_2026_809_MOESM3_ESM.zip › Source_Data_Figure_1/1J/1J-40 min .tif]

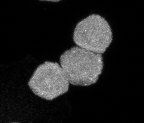

Supplement: Supplementary file 4 — Source data Fig. 2 [file 44319_2026_809_MOESM4_ESM.zip › Source_Data_Figure_2/2B/2B egg5 arrest Lamin-B1.tif]

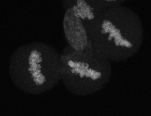

Supplement: Supplementary file 4 — Source data Fig. 2 [file 44319_2026_809_MOESM4_ESM.zip › Source_Data_Figure_2/2B/2B APC:Ci DAPItif.tif]

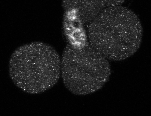

Supplement: Supplementary file 4 — Source data Fig. 2 [file 44319_2026_809_MOESM4_ESM.zip › Source_Data_Figure_2/2B/2B APC:Ci Lamin B1.tif]

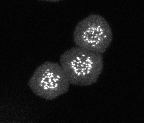

Supplement: Supplementary file 4 — Source data Fig. 2 [file 44319_2026_809_MOESM4_ESM.zip › Source_Data_Figure_2/2B/2B egg5 arrest Incenp.tif]

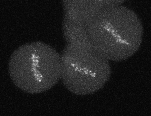

Supplement: Supplementary file 4 — Source data Fig. 2 [file 44319_2026_809_MOESM4_ESM.zip › Source_Data_Figure_2/2B/2B APC:Ci Incenp.tif]

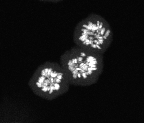

Supplement: Supplementary file 4 — Source data Fig. 2 [file 44319_2026_809_MOESM4_ESM.zip › Source_Data_Figure_2/2B/2B egg5 arrest DAPI.tif]

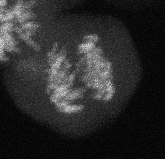

Supplement: Supplementary file 4 — Source data Fig. 2 [file 44319_2026_809_MOESM4_ESM.zip › Source_Data_Figure_2/2C/DMSO/2C early anaphase DAPI.tif]

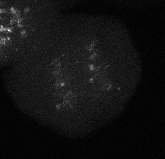

Supplement: Supplementary file 4 — Source data Fig. 2 [file 44319_2026_809_MOESM4_ESM.zip › Source_Data_Figure_2/2C/DMSO/2C early anaphase Incenp.tif]

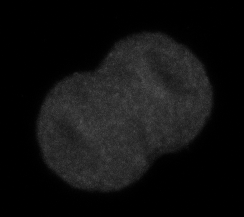

Supplement: Supplementary file 4 — Source data Fig. 2 [file 44319_2026_809_MOESM4_ESM.zip › Source_Data_Figure_2/2C/DMSO/2C anaphase Midzone lamin B.tif]

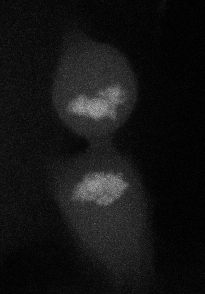

Supplement: Supplementary file 4 — Source data Fig. 2 [file 44319_2026_809_MOESM4_ESM.zip › Source_Data_Figure_2/2C/DMSO/2C telophase DAPI.tif]

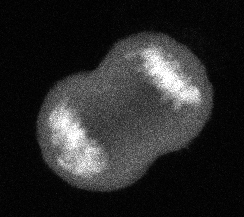

Supplement: Supplementary file 4 — Source data Fig. 2 [file 44319_2026_809_MOESM4_ESM.zip › Source_Data_Figure_2/2C/DMSO/2C anaphase Midzone DAPI.tif]

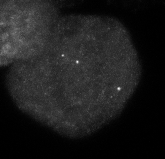

Supplement: Supplementary file 4 — Source data Fig. 2 [file 44319_2026_809_MOESM4_ESM.zip › Source_Data_Figure_2/2C/DMSO/2C early anaphase Lamin B.tif]

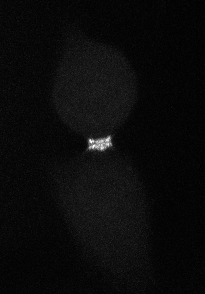

Supplement: Supplementary file 4 — Source data Fig. 2 [file 44319_2026_809_MOESM4_ESM.zip › Source_Data_Figure_2/2C/DMSO/2C telophase Incenp.tif]

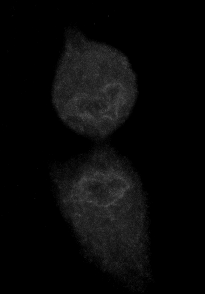

Supplement: Supplementary file 4 — Source data Fig. 2 [file 44319_2026_809_MOESM4_ESM.zip › Source_Data_Figure_2/2C/DMSO/2C telophase Lamin B.tif]

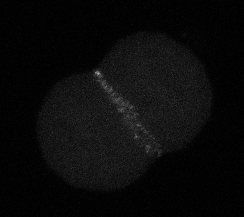

Supplement: Supplementary file 4 — Source data Fig. 2 [file 44319_2026_809_MOESM4_ESM.zip › Source_Data_Figure_2/2C/DMSO/2C anaphase Midzone Incenp.tif]

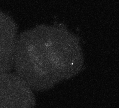

Supplement: Supplementary file 4 — Source data Fig. 2 [file 44319_2026_809_MOESM4_ESM.zip › Source_Data_Figure_2/2C/AZD 1775/2C early anaphase Incenp AZD.tif]

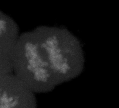

Supplement: Supplementary file 4 — Source data Fig. 2 [file 44319_2026_809_MOESM4_ESM.zip › Source_Data_Figure_2/2C/AZD 1775/2C early anaphase DAPI AZD.tif]

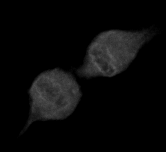

Supplement: Supplementary file 4 — Source data Fig. 2 [file 44319_2026_809_MOESM4_ESM.zip › Source_Data_Figure_2/2C/AZD 1775/2C Telophase AZD Lamin B.tif]

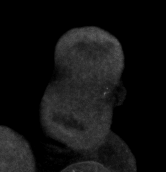

Supplement: Supplementary file 4 — Source data Fig. 2 [file 44319_2026_809_MOESM4_ESM.zip › Source_Data_Figure_2/2C/AZD 1775/2C Anaphase AZD lamin B.tif]

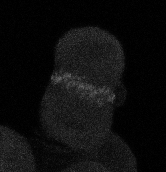

Supplement: Supplementary file 4 — Source data Fig. 2 [file 44319_2026_809_MOESM4_ESM.zip › Source_Data_Figure_2/2C/AZD 1775/2C Anaphase AZD Incenp.tif]

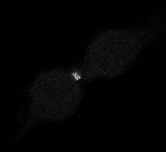

Supplement: Supplementary file 4 — Source data Fig. 2 [file 44319_2026_809_MOESM4_ESM.zip › Source_Data_Figure_2/2C/AZD 1775/2C Telophase AZD Incenp.tif]

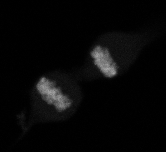

Supplement: Supplementary file 4 — Source data Fig. 2 [file 44319_2026_809_MOESM4_ESM.zip › Source_Data_Figure_2/2C/AZD 1775/2C Telophase AZD DAPI.tif]

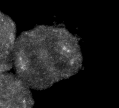

Supplement: Supplementary file 4 — Source data Fig. 2 [file 44319_2026_809_MOESM4_ESM.zip › Source_Data_Figure_2/2C/AZD 1775/2C early anaphase Lamin B AZD.tif]

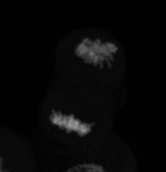

Supplement: Supplementary file 4 — Source data Fig. 2 [file 44319_2026_809_MOESM4_ESM.zip › Source_Data_Figure_2/2C/AZD 1775/2C Anaphase AZD DAPI.tif]

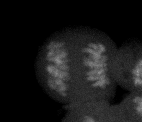

Supplement: Supplementary file 4 — Source data Fig. 2 [file 44319_2026_809_MOESM4_ESM.zip › Source_Data_Figure_2/2C/RP 6306/2C early anaphase DAPI RP.tif]

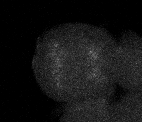

Supplement: Supplementary file 4 — Source data Fig. 2 [file 44319_2026_809_MOESM4_ESM.zip › Source_Data_Figure_2/2C/RP 6306/2C early anaphase Incenp RP.tif]

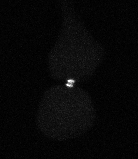

Supplement: Supplementary file 4 — Source data Fig. 2 [file 44319_2026_809_MOESM4_ESM.zip › Source_Data_Figure_2/2C/RP 6306/2C telophase Incenp RP.tif]

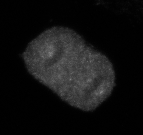

Supplement: Supplementary file 4 — Source data Fig. 2 [file 44319_2026_809_MOESM4_ESM.zip › Source_Data_Figure_2/2C/RP 6306/2C anaphase Lamin B RP.tif]

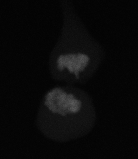

Supplement: Supplementary file 4 — Source data Fig. 2 [file 44319_2026_809_MOESM4_ESM.zip › Source_Data_Figure_2/2C/RP 6306/2C telophase DAPI RP.tif]

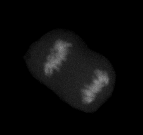

Supplement: Supplementary file 4 — Source data Fig. 2 [file 44319_2026_809_MOESM4_ESM.zip › Source_Data_Figure_2/2C/RP 6306/2C anaphase DAPI RP.tif]

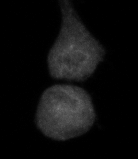

Supplement: Supplementary file 4 — Source data Fig. 2 [file 44319_2026_809_MOESM4_ESM.zip › Source_Data_Figure_2/2C/RP 6306/2C telophase Lamin B RP.tif]

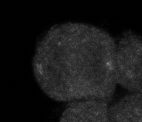

Supplement: Supplementary file 4 — Source data Fig. 2 [file 44319_2026_809_MOESM4_ESM.zip › Source_Data_Figure_2/2C/RP 6306/2C early anaphase Lamin B RP.tif]

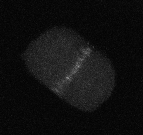

Supplement: Supplementary file 4 — Source data Fig. 2 [file 44319_2026_809_MOESM4_ESM.zip › Source_Data_Figure_2/2C/RP 6306/2C anaphase Incenp RP.tif]

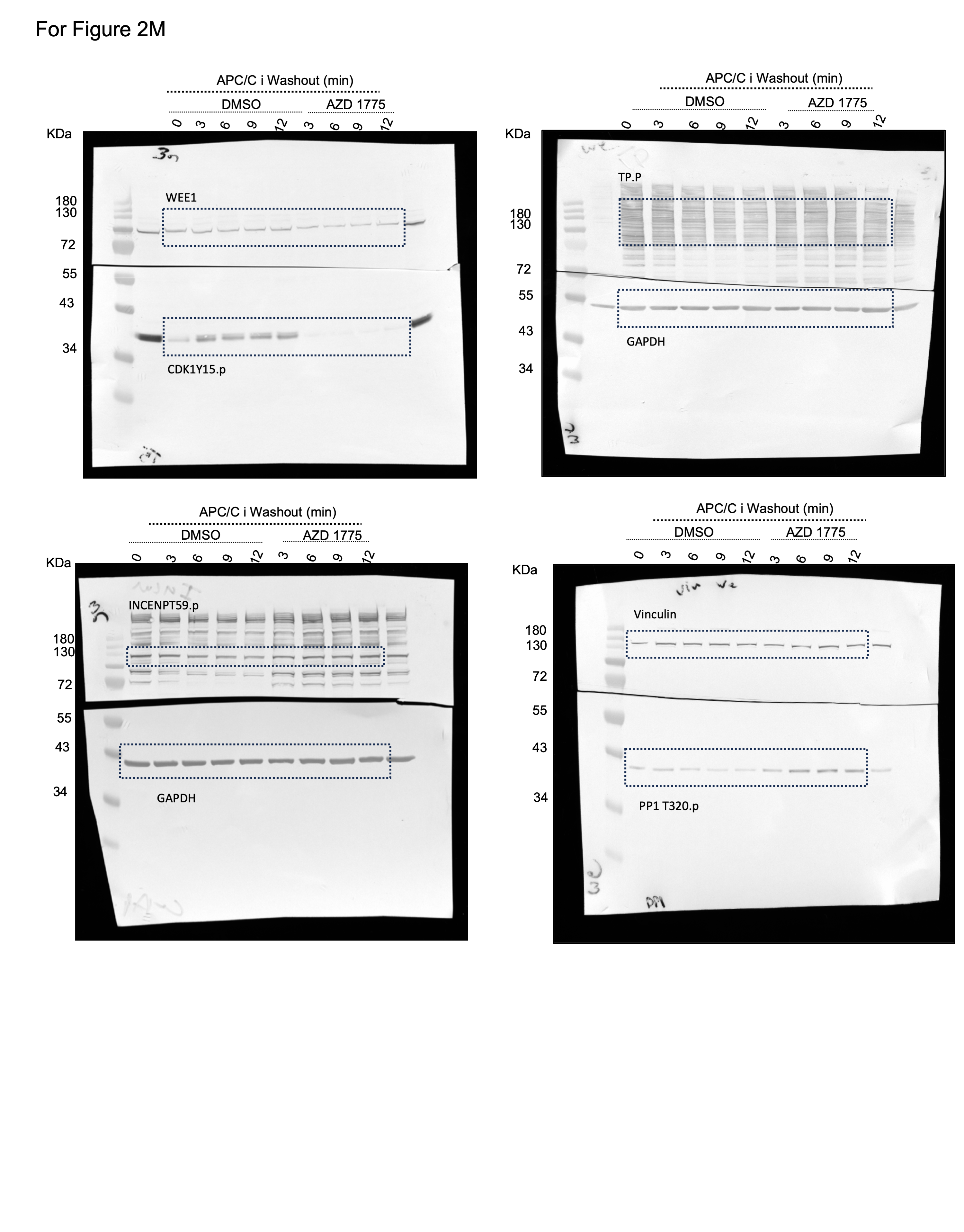

Supplement: Supplementary file 5 — Source data Fig. 3 [file 44319_2026_809_MOESM5_ESM.zip › Source_Data_Figure_3/3B/3B.tiff]

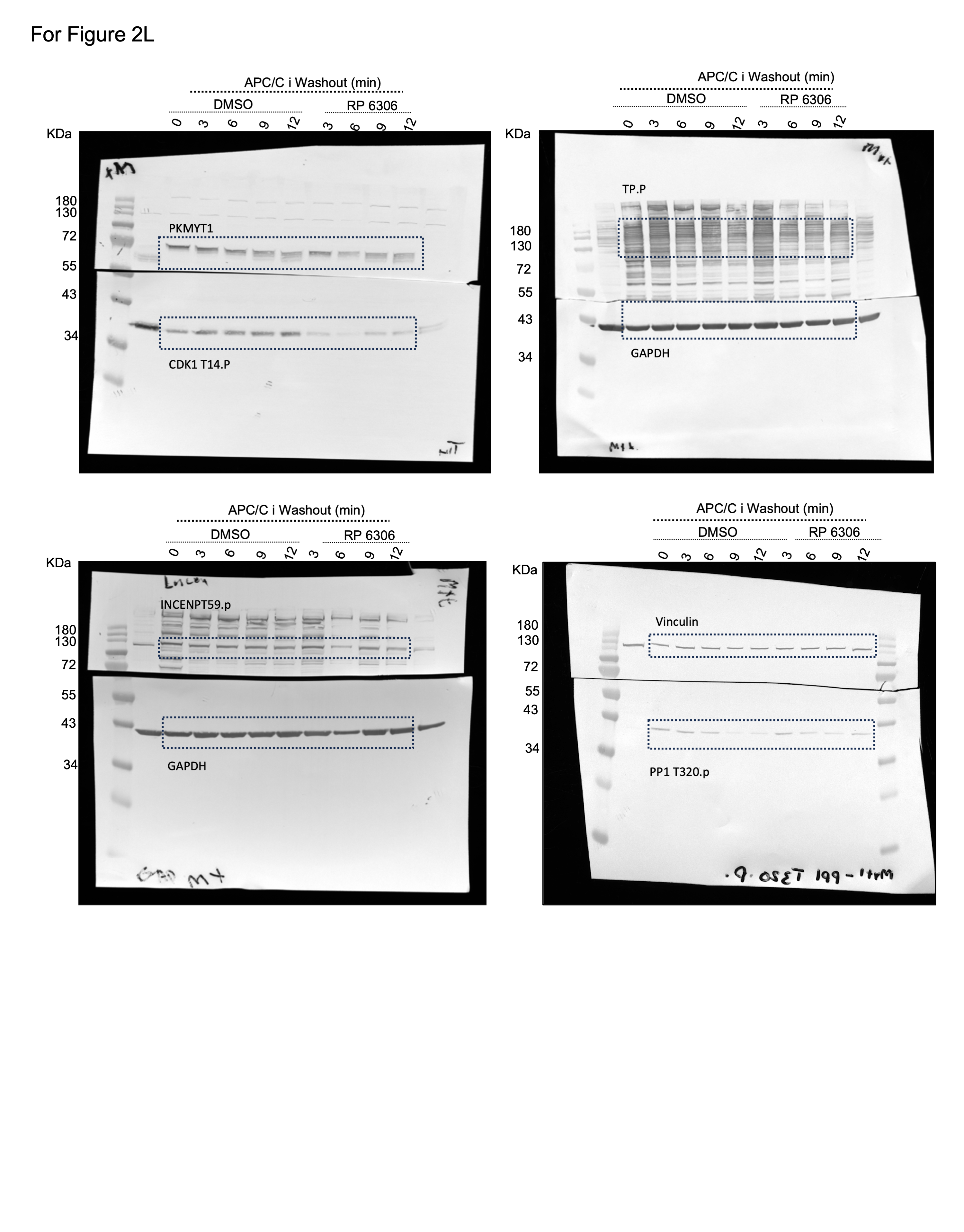

Supplement: Supplementary file 5 — Source data Fig. 3 [file 44319_2026_809_MOESM5_ESM.zip › Source_Data_Figure_3/3A/3A.tiff]

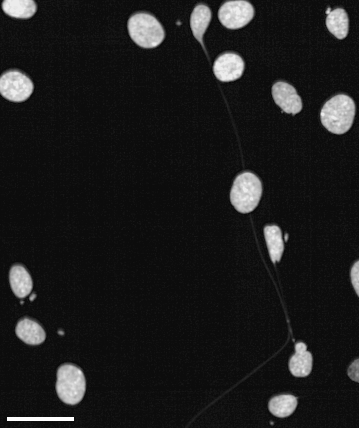

Supplement: Supplementary file 6 — Source data Fig. 4 [file 44319_2026_809_MOESM6_ESM.zip › Source_Data_Figure_4/4C/4C.tif]

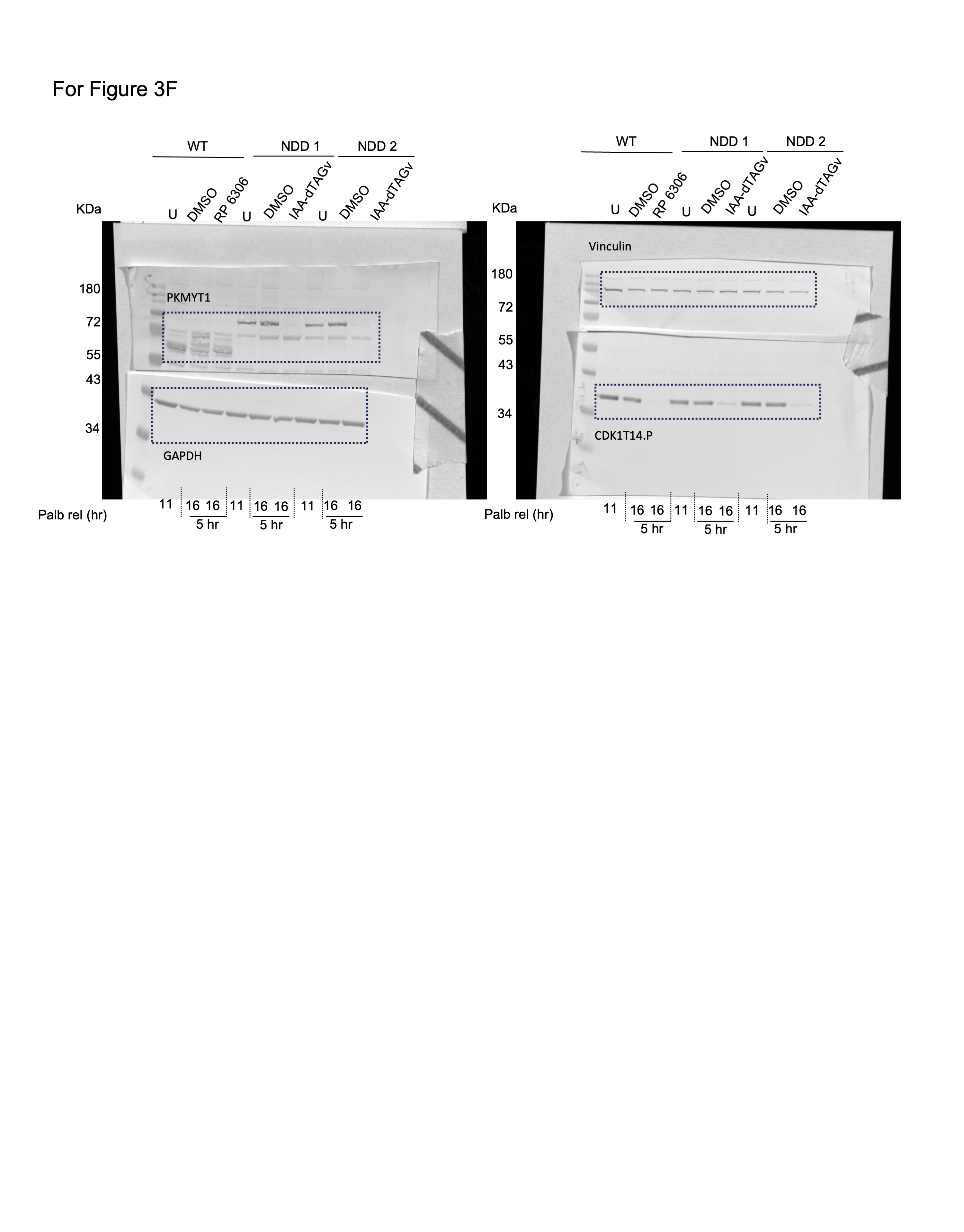

Supplement: Supplementary file 6 — Source data Fig. 4 [file 44319_2026_809_MOESM6_ESM.zip › Source_Data_Figure_4/4F/4F.tiff]

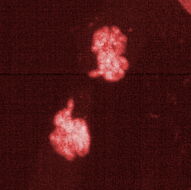

Supplement: Supplementary file 7 — Source data Fig. 5 [file 44319_2026_809_MOESM7_ESM.zip › Source_Data_Figure_5/5A/5A AZD 1775/5A AZD 1775 48.04 min.tif]

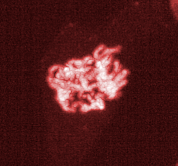

Supplement: Supplementary file 7 — Source data Fig. 5 [file 44319_2026_809_MOESM7_ESM.zip › Source_Data_Figure_5/5A/5A AZD 1775/5A AZD 1775 0.0 min.tif]

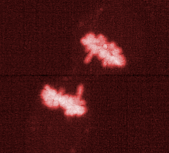

Supplement: Supplementary file 7 — Source data Fig. 5 [file 44319_2026_809_MOESM7_ESM.zip › Source_Data_Figure_5/5A/5A AZD 1775/5A AZD 1775 36.10 min.tif]

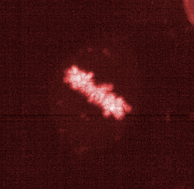

Supplement: Supplementary file 7 — Source data Fig. 5 [file 44319_2026_809_MOESM7_ESM.zip › Source_Data_Figure_5/5A/5A AZD 1775/5A AZD 1775 28.12 min.tif]

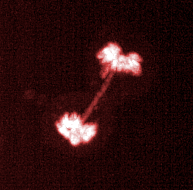

Supplement: Supplementary file 7 — Source data Fig. 5 [file 44319_2026_809_MOESM7_ESM.zip › Source_Data_Figure_5/5A/5A PKMYT -:-/5A PKMYT1 -:- 30.03 min.tif]

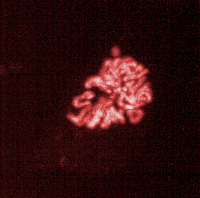

Supplement: Supplementary file 7 — Source data Fig. 5 [file 44319_2026_809_MOESM7_ESM.zip › Source_Data_Figure_5/5A/5A PKMYT -:-/5A PKMYT -:- 0.0 min.tif]

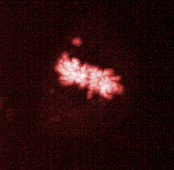

Supplement: Supplementary file 7 — Source data Fig. 5 [file 44319_2026_809_MOESM7_ESM.zip › Source_Data_Figure_5/5A/5A PKMYT -:-/5A PKMYT -:- 22.17 min.tif]

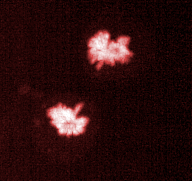

Supplement: Supplementary file 7 — Source data Fig. 5 [file 44319_2026_809_MOESM7_ESM.zip › Source_Data_Figure_5/5A/5A PKMYT -:-/5A PKMYT -:- 34.39 min.tif]

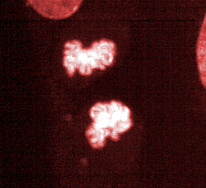

Supplement: Supplementary file 7 — Source data Fig. 5 [file 44319_2026_809_MOESM7_ESM.zip › Source_Data_Figure_5/5A/5A DMSO/5A DMSO 41.17 min .tif]

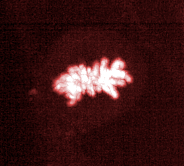

Supplement: Supplementary file 7 — Source data Fig. 5 [file 44319_2026_809_MOESM7_ESM.zip › Source_Data_Figure_5/5A/5A DMSO/5A DMSO 23.24 min.tif]

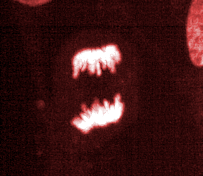

Supplement: Supplementary file 7 — Source data Fig. 5 [file 44319_2026_809_MOESM7_ESM.zip › Source_Data_Figure_5/5A/5A DMSO/5A DMSO 30.05 min.tif]

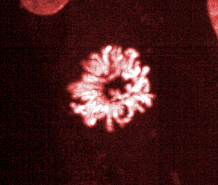

Supplement: Supplementary file 7 — Source data Fig. 5 [file 44319_2026_809_MOESM7_ESM.zip › Source_Data_Figure_5/5A/5A DMSO/5A DMSO 0.0 min.tif]

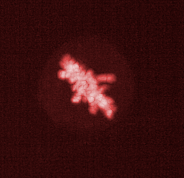

Supplement: Supplementary file 7 — Source data Fig. 5 [file 44319_2026_809_MOESM7_ESM.zip › Source_Data_Figure_5/5A/5A RP 6306/5A RP 6306 24.14 min.tif]

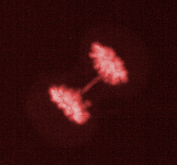

Supplement: Supplementary file 7 — Source data Fig. 5 [file 44319_2026_809_MOESM7_ESM.zip › Source_Data_Figure_5/5A/5A RP 6306/5A RP 6306 31.34 min.tif]

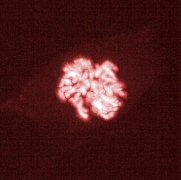

Supplement: Supplementary file 7 — Source data Fig. 5 [file 44319_2026_809_MOESM7_ESM.zip › Source_Data_Figure_5/5A/5A RP 6306/5A RP 6306 0.0 min.tif]

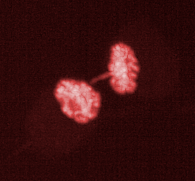

Supplement: Supplementary file 7 — Source data Fig. 5 [file 44319_2026_809_MOESM7_ESM.zip › Source_Data_Figure_5/5A/5A RP 6306/5A RP 6306 41.04. mintif.tif]

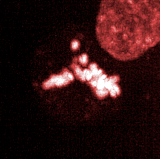

Supplement: Supplementary file 7 — Source data Fig. 5 [file 44319_2026_809_MOESM7_ESM.zip › Source_Data_Figure_5/5C/DMSO/5C DMSO 2.09 hr.tif]

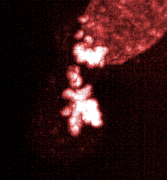

Supplement: Supplementary file 7 — Source data Fig. 5 [file 44319_2026_809_MOESM7_ESM.zip › Source_Data_Figure_5/5C/DMSO/5C DMSO 2.27 hr.tif]

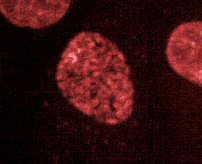

Supplement: Supplementary file 7 — Source data Fig. 5 [file 44319_2026_809_MOESM7_ESM.zip › Source_Data_Figure_5/5C/DMSO/5C DMSO -00.10 hr.tif]

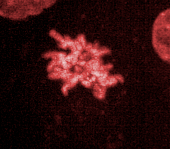

Supplement: Supplementary file 7 — Source data Fig. 5 [file 44319_2026_809_MOESM7_ESM.zip › Source_Data_Figure_5/5C/DMSO/5C DMSO 0.00 hr.tif]

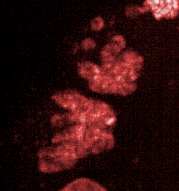

Supplement: Supplementary file 7 — Source data Fig. 5 [file 44319_2026_809_MOESM7_ESM.zip › Source_Data_Figure_5/5C/DMSO/5C DMSO 2.55 hr.tif]

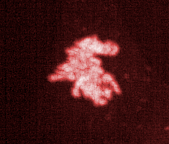

Supplement: Supplementary file 7 — Source data Fig. 5 [file 44319_2026_809_MOESM7_ESM.zip › Source_Data_Figure_5/5C/AZD 1775/5C AZD 1775 0.0 min.tif]

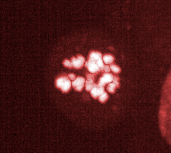

Supplement: Supplementary file 7 — Source data Fig. 5 [file 44319_2026_809_MOESM7_ESM.zip › Source_Data_Figure_5/5C/AZD 1775/5C AZD 1775 11.25 min.tif]

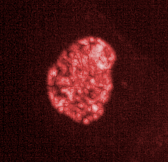

Supplement: Supplementary file 7 — Source data Fig. 5 [file 44319_2026_809_MOESM7_ESM.zip › Source_Data_Figure_5/5C/AZD 1775/5C AZD 1775 -00.10 min.tif]

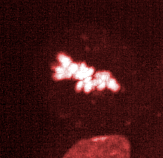

Supplement: Supplementary file 7 — Source data Fig. 5 [file 44319_2026_809_MOESM7_ESM.zip › Source_Data_Figure_5/5C/AZD 1775/5C AZD 1775 1.20 hr .tif]

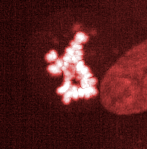

Supplement: Supplementary file 7 — Source data Fig. 5 [file 44319_2026_809_MOESM7_ESM.zip › Source_Data_Figure_5/5C/AZD 1775/5C AZD 1775 7.25 hr.tif]

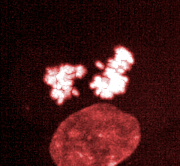

Supplement: Supplementary file 7 — Source data Fig. 5 [file 44319_2026_809_MOESM7_ESM.zip › Source_Data_Figure_5/5C/RP 6306/5C RP 6306 01.27 hr.tif]

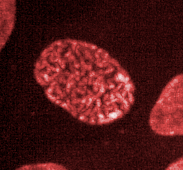

Supplement: Supplementary file 7 — Source data Fig. 5 [file 44319_2026_809_MOESM7_ESM.zip › Source_Data_Figure_5/5C/RP 6306/5C RP 6306 -0.10 hr.tif]

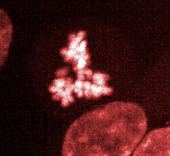

Supplement: Supplementary file 7 — Source data Fig. 5 [file 44319_2026_809_MOESM7_ESM.zip › Source_Data_Figure_5/5C/RP 6306/5C RP 6306 1.10 hr.tif]

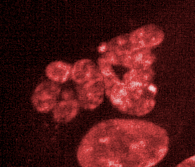

Supplement: Supplementary file 7 — Source data Fig. 5 [file 44319_2026_809_MOESM7_ESM.zip › Source_Data_Figure_5/5C/RP 6306/5C RP 6306 2.0 hr.tif]

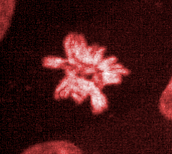

Supplement: Supplementary file 7 — Source data Fig. 5 [file 44319_2026_809_MOESM7_ESM.zip › Source_Data_Figure_5/5C/RP 6306/5C RP 6306 0.00.tif]
