# Supplementary material for: PKMYT1 has an important role in the timing and fidelity of chromosome segregation
Source: EMBO Rep. 2026 Jun 5;27(13):3564–84. doi: 10.1038/s44319-026-00809-1 (PMC13354794; doi:10.1038/s44319-026-00809-1)
Supplement: Supplementary file 7 — Source data Fig. 5 [file 44319_2026_809_MOESM7_ESM.zip › Source_Data_Figure_5/5C/RP 6306/Movie_5C_Description.rtf]

This movie provides the source data for the snapshots shown in Figure 5C. It depicts live RPE-1 cells treated with 5 nM of Paclitaxel with RP 6306 (500 nM).
