# Supplementary material for: PKMYT1 has an important role in the timing and fidelity of chromosome segregation
Source: EMBO Rep. 2026 Jun 5;27(13):3564–84. doi: 10.1038/s44319-026-00809-1 (PMC13354794; doi:10.1038/s44319-026-00809-1)
Supplement: Supplementary file 8 — Expanded View Figures [file 44319_2026_809_MOESM8_ESM.pdf]

# Expanded View Figures

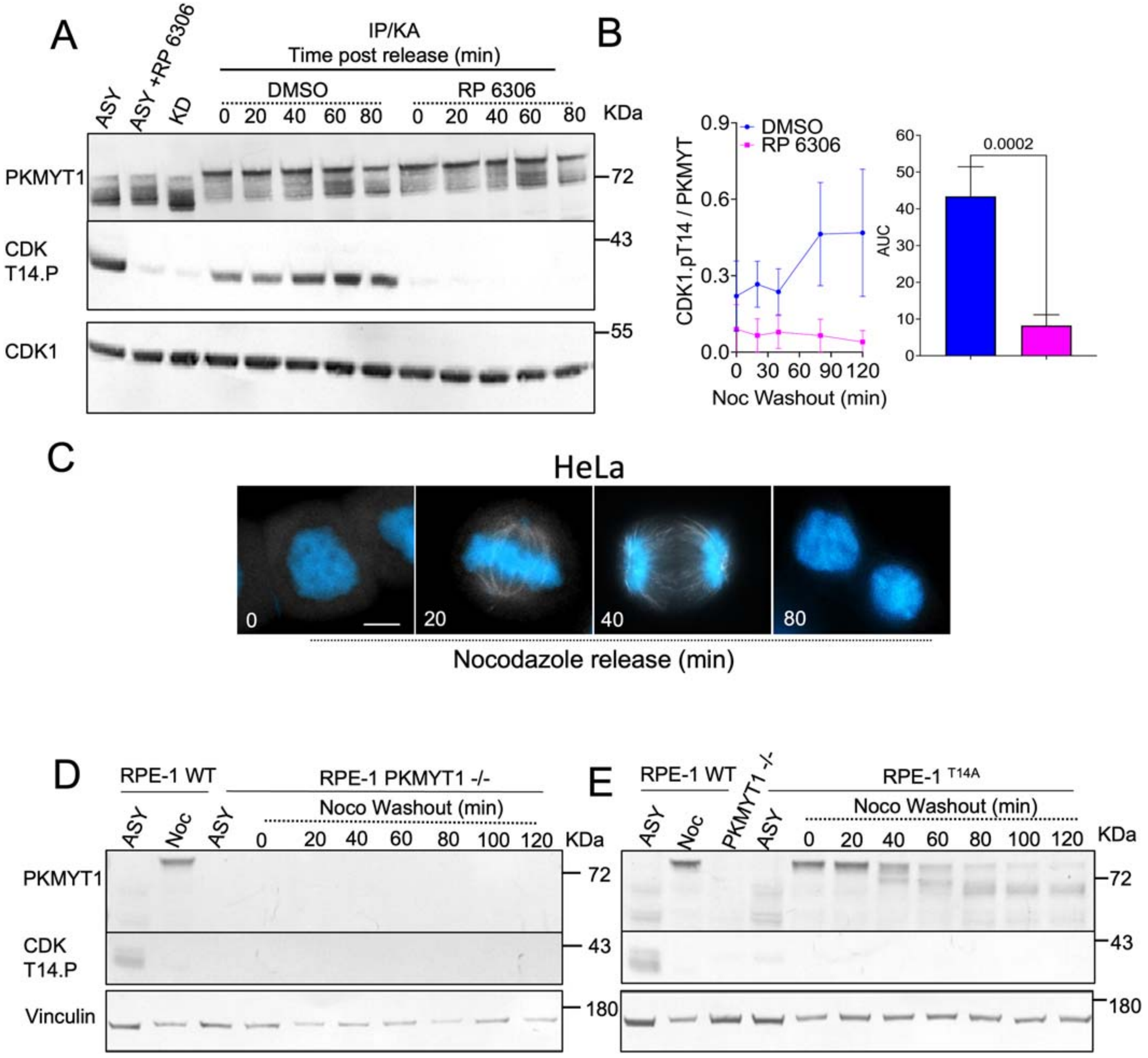


**Figure EV1. Validation of PKMYT1 Kinase Activity and CDK1 Phosphorylation Kinetics in Mitotic Exit.**

(**A**) In vitro kinase assay was performed using PKMYT1 immunoprecipitated from mitotic HeLa cell. Lysates were collected every 20 min following release from a nocodazole shake-off. The reaction was conducted as described in Fig. 1**F**, **G**. The samples were analyzed by 12% SDS-PAGE and immunoblotted for PKMYT1, P-T14-CDK1. Total CDK1 was detected on a parallel membrane as a sample processing and loading control. "ASY" indicates an asynchronous cell culture control and "KD" indicates Kinase-Dead purified PKMYT1. (**B**) Quantification of P-T14-CDK1 phosphorylation relative to PKMYT1 levels from the in vitro kinase assays shown in (**A**). Data were presented as mean ± SD, $n = 3$ biological replicates. Statistical analysis was performed using the area under the curve (AUC) by a Student's *t*-test. (**C**) Representative confocal microscopy images of HeLa cells fixed post-nocodazole release, showing progression through mitosis. Scale bar, 5 µm. (**D**, **E**) Analysis of mitotic lysates from two cell lines with altered PKMYT1 activity: RPE1 PKMYT1 knockout (−/−) and RPE1 $CDK1^{T14A}$. Cells were arrested with nocodazole and then released into fresh media. Cell lysates were collected every 10 min post-release, separated by 12% SDS-PAGE, and immunoblotted for PKMYT1, phospho-threonine 14 CDK1 (P-T14-CDK1), with Vinculin run on a parallel membrane as a sample processing control. Data from the PKMYT1 −/− cell line are shown in (**D**), and data from the CDK1 $^{T14A}$ cell line are shown in (**E**).

 © The Author(s)

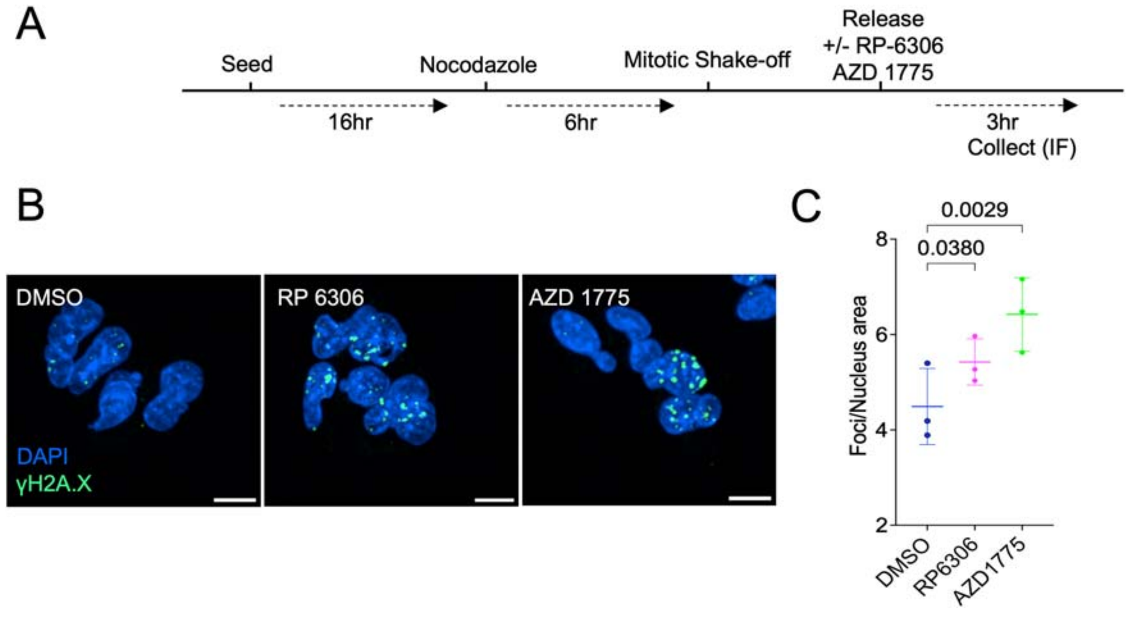


**Figure EV2. Mitotic PKMYT1 and WEE1 Inhibition Induces DNA Damage.**

(**A**) Outline of the experimental workflow for nocodazole shake-off of RPE1 cells. (**B**) Representative maximum intensity projections of confocal images of cells post-nocodazole release, exposed to either DMSO, RP 6306 (500 nM), or AZD 1775 (300 nM). Cells were stained with DAPI (DNA) and γH2A.X as an indicator for double-strand breaks. Scale bar, 10 μm. (**C**) Quantification of γH2A.X-positive foci per nucleus. Mean ± SD, $n = 3$ biological replicates is shown. One-way ANOVA was used for statistical analysis.

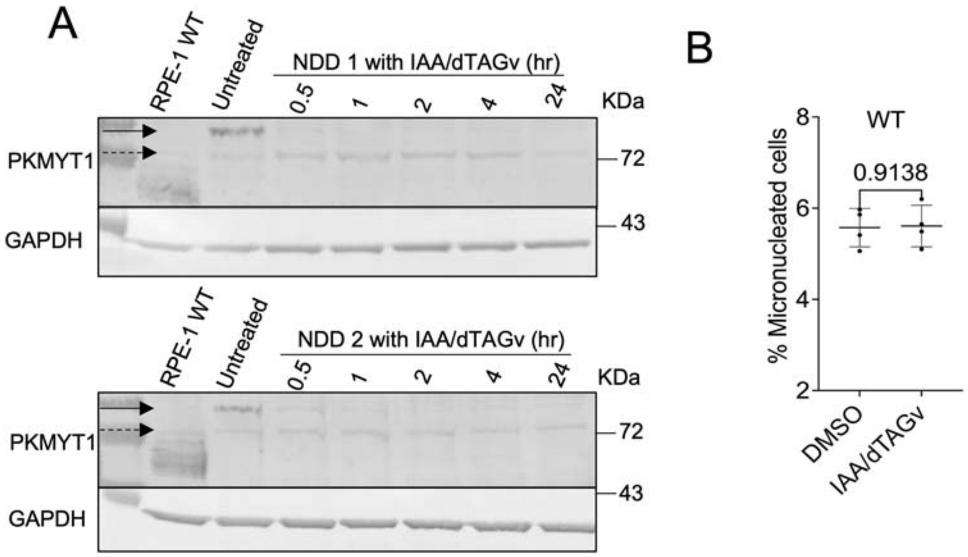


**Figure EV3. Use of a degron-tagged system to induce acute PKMYT1 degradation.**

(**A**) Acute degradation of degron-tagged PKMYT1 (clones NDD 1 and NDD 2). Cells with degron-tagged PKMYT1 were treated with 5-Ph-IAA-dTAGv for the indicated time points. Cell lysates were collected, separated by 12% SDS-PAGE, and immunoblotted for PKMYT1 and GAPDH detected on the same membrane to serve as a loading control. The solid arrow indicates the tagged PKMYT1, and the dotted arrow indicates a non-specific band. (**B**) Rate of micronucleation of WT RPE1 cells treated with IAA-dTAGv. Untagged RPE1 cells were synchronized with palbociclib for 24 h and then released into fresh medium. After 11 h, cells were treated with either DMSO or 5-Ph-IAA -dTAGv for 5 h. Cells were then fixed and stained for DNA to quantify micronucleation. Data represent the mean ± SD, $n = 4$ biological replicates. Statistical significance was determined using a Student's *t*-test.

 © The Author(s)

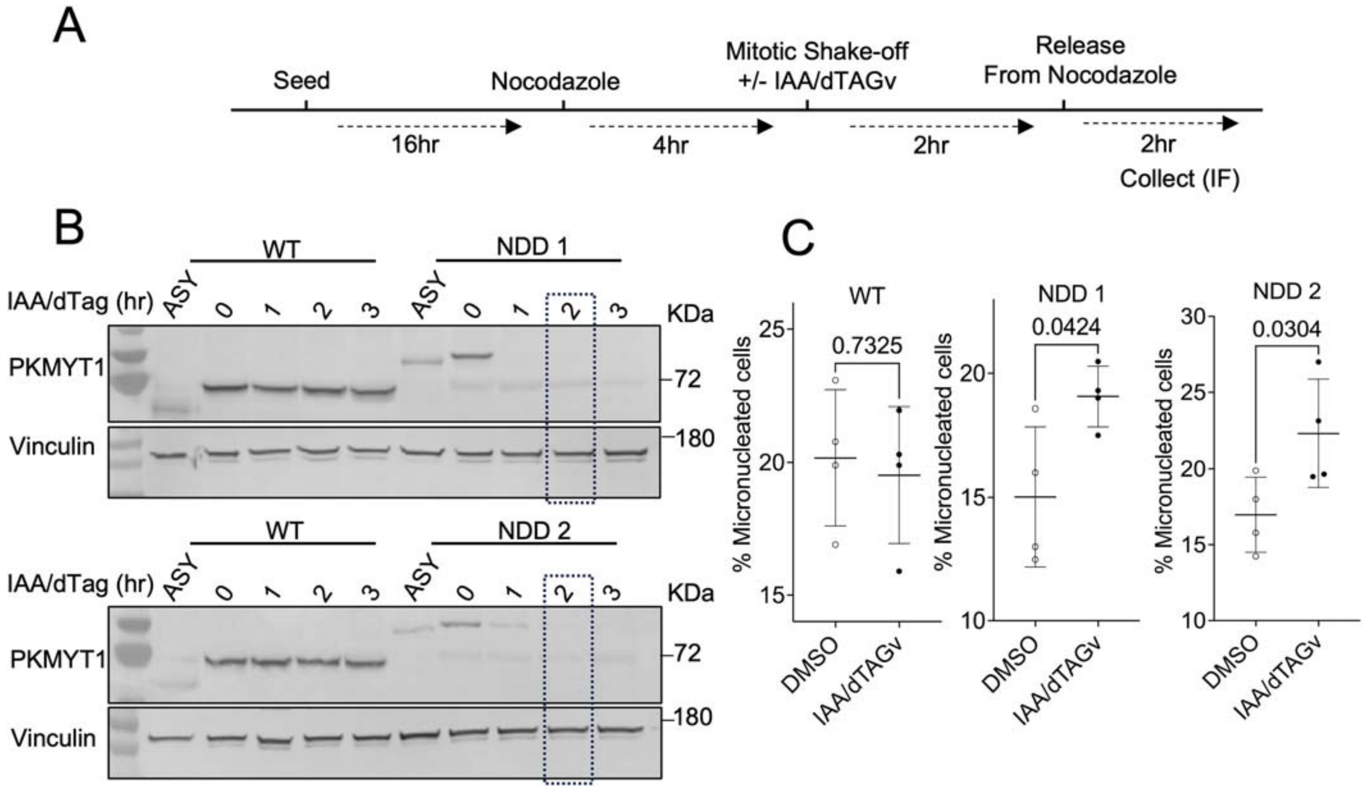


**Figure EV4. Use of a dual-degron system to induce acute mitotic PKMYT1 degradation.**

(**A**) Outline of the experimental workflow for nocodazole shake-off of RPE1 cells. RPE1 wild-type (WT) and two RPE1 clones expressing degron-tagged PKMYT1 (clones NDD 1 and NDD 2) were synchronized as indicated. (**B**) WT and NDD clones were treated with DMSO or a ligand (0.5 µM 5-Ph-IAA and 0.1 µM dTAGv). Cell lysates were collected every hour for 3 h while cells remained arrested in nocodazole. Equal amounts of protein lysate were resolved by 12% SDS-PAGE. Membranes were immunoblotted for PKMYT1, while Vinculin was detected on a parallel membrane to serve as a sample processing control. ASY denotes asynchronous cells. (**C**) Quantification of the micronucleation rate in WT and NDD clones post-nocodazole washout. WT and NDD clones were synchronized as in (**A**) and treated with DMSO or the degradation ligand (0.5 µM 5-Ph-IAA and 0.1 µM dTAGv) for 2 h during the arrest. Cells were then released into fresh media to allow for mitotic progression. After 2 h of nocodazole washout, cells were fixed and stained with DAPI to visualize micronucleation. Data represent the mean ± SD, $n = 4$ biological replicates. Statistical significance was determined using a Student's *t*-test.
