# Supplementary material for: PKMYT1 has an important role in the timing and fidelity of chromosome segregation
Source: EMBO Rep. 2026 Jun 5;27(13):3564–84. doi: 10.1038/s44319-026-00809-1 (PMC13354794; doi:10.1038/s44319-026-00809-1)
Supplement: Supplementary file 2 — Peer Review File [file 44319_2026_809_MOESM2_ESM.pdf]

EMBO reports

PKMYT1 has an Important Role in the Timing and Fidelity of Chromosome Segregation

Asma Belbelazi, Alexandra Hendry, Eleanor Trotter, Charlie Greenaway-Wells, Zoe Edwards, Keren Dawson, Jonathon Pines, and Iain Hagan

Corresponding author(s): Asma Belbelazi (asma.belbelazi@cruk.manchester.ac.uk)

Review Timeline:

Submission Date:	13th Oct 25
Editorial Decision:	2nd Dec 25
Revision Received:	27th Feb 26
Editorial Decision:	10th Apr 26
Revision Received:	5th May 26
Accepted:	18th May 26

Editor: Achim Breiling

Transaction Report:

(Note: With the exception of the correction of typographical or spelling errors that could be a source of ambiguity, letters and reports are not edited. Depending on transfer agreements, referee reports obtained elsewhere may or may not be included in this compilation. Referee reports are anonymous unless the Referee chooses to sign their reports.)

1st Editorial Decision 2nd Dec 2025

Dear Dr. Belbelazi,

Thank you for the submission of your manuscript to EMBO reports. I have now received reports from two of the three referees that were asked to evaluate your study, which can be found at the end of this email. A third referee agreed to look into this, but never submitted a report, despite several reminders. I thus decided to proceed without that input.

As you will see, the referees think that these findings are of interest. However, they have several comments, concerns, and suggestions, indicating that a major revision of the manuscript is necessary to allow publication of the study in EMBO reports. As the reports are below, and all the referee concerns need to be addressed, I will not detail them here.

Given the constructive referee comments, I would thus like to invite you to revise your manuscript with the understanding that the concerns of the referees must be addressed in the revised manuscript and/or in a detailed point-by-point response. Acceptance of your manuscript will depend on a positive outcome of a second round of review. It is EMBO reports policy to allow a single round of revision only and acceptance of the manuscript will therefore depend on the completeness of your responses included in the next, final version of the manuscript.

Revised manuscripts should be submitted within three months of a request for revision. Please contact me to discuss the revision (also by video chat) if you have questions or comments regarding the revision, or should you need additional time.

When submitting your revised manuscript, please also carefully review the instructions that follow below.

PLEASE NOTE THAT upon resubmission revised manuscripts are subjected to an initial quality control prior to exposition to re-review. Upon failure in the initial quality control, the manuscripts are sent back to the authors, which may lead to delays. Frequent reasons for such a failure are the lack of the data availability section (please see below) and the presence of statistics based on n=2 (the authors are then asked to present scatter plots or provide more data points).

When submitting your revised manuscript, we will require:

1) a .docx formatted version of the final manuscript text (including legends for main figures, EV figures and tables), but without the figures included. Figure legends should be compiled at the end of the manuscript text.

2) individual production quality figure files as .eps, .tif, .jpg (one file per figure), of main figures and EV figures. Please upload these as separate, individual files upon re-submission.

The Expanded View format, which will be displayed in the main HTML of the paper in a collapsible format, has replaced the Supplementary information. You can submit up to 6 images as Expanded View. Please follow the nomenclature Figure EV1, Figure EV2 etc. The figure legend for these should be included in the main manuscript document file in a section called Expanded View Figure Legends after the main Figure Legends section. Additional Supplementary material should be supplied as a single pdf file labeled Appendix. The Appendix should have page numbers and needs to include a table of content on the first page (with page numbers) and legends for all content. Please follow the nomenclature Appendix Figure Sx, Appendix Table Sx etc. throughout the text, and also label the figures and tables according to this nomenclature.

For more details, please refer to our guide to authors:
http://www.embopress.org/page/journal/14693178/authorguide#manuscriptpreparation

Please consult our guide for figure preparation:
http://wol-prod-cdn.literatumonline.com/pb-assets/embo-site/EMBOPress_Figure_Guidelines_061115-1561436025777.pdf

See also the guidelines for figure legend preparation:
https://www.embopress.org/page/journal/14693178/authorguide#figureformat

3) a .docx formatted letter INCLUDING the reviewers' reports and your detailed point-by-point responses to their comments. As part of the EMBO Press transparent editorial process, the point-by-point response is part of the Review Process File (RPF), which will be published alongside your paper.

4) a complete author checklist, which you can download from our author guidelines (https://www.embopress.org/page/journal/14693178/authorguide). Please insert page numbers in the checklist to indicate where the requested information can be found in the manuscript. The completed author checklist will also be part of the RPF.

Please also follow our guidelines for the use of living organisms, and the respective reporting guidelines:
http://www.embopress.org/page/journal/14693178/authorguide#livingorganisms

5) that primary datasets produced in this study (e.g. RNA-seq, ChIP-seq, structural and array data) are deposited in an appropriate public database. If no primary datasets have been deposited, please also state this in a dedicated section (e.g. 'No primary datasets have been generated and deposited'), see below.

See also: http://embor.embopress.org/authorguide#datadeposition

Please remember to provide a reviewer password if the datasets are not yet public.

The accession numbers and database should be listed in a formal "Data Availability" section that follows the model below. This is now mandatory (like the COI statement). Please note that the Data Availability Section is restricted to new primary data that are part of this study. This section is mandatory. As indicated above, if no primary datasets have been deposited, please state this in this section

Data availability

The datasets produced in this study are available in the following databases:

- RNA-Seq data: Gene Expression Omnibus GSE46843 (https://www.ncbi.nlm.nih.gov/geo/query/acc.cgi?acc=GSE46843)
- [data type]: [name of the resource] [accession number/identifier/doi] ([URL or identifiers.org/DATABASE:ACCESSION])

*** Note - All links should resolve to a page where the data can be accessed. ***

Moreover, I have these editorial requests:

6) We now request the publication of original source data with the aim of making primary data more accessible and transparent to the reader. You will receive a separate email with instructions for providing source data with your revised manuscript, including information how to upload and organize the files.

7) Our journal encourages inclusion of *data citations in the reference list* to directly cite datasets that were re-used and obtained from public databases. Data citations in the article text are distinct from normal bibliographical citations and should directly link to the database records from which the data can be accessed. In the main text, data citations are formatted as follows: "Data ref: Smith et al, 2001" or "Data ref: NCBI Sequence Read Archive PRJNA342805, 2017". In the Reference list, data citations must be labeled with "[DATASET]". A data reference must provide the database name, accession number/identifiers and a resolvable link to the landing page from which the data can be accessed at the end of the reference. Further instructions are available at: http://www.embopress.org/page/journal/14693178/authorguide#referencesformat

8) Regarding data quantification and statistics, please make sure that the number "n" for how many independent experiments were performed, their nature (biological versus technical replicates), the bars and error bars (e.g. SEM, SD) and the test used to calculate p-values is indicated in the respective figure legends (also for EV and Appendix figures). Please also check that all the p-values are explained in the legend, and that these fit to those shown in the figure. Please provide statistical testing where applicable. Please avoid the phrase 'independent experiment' but clearly state if these were biological or technical replicates. Please also indicate (e.g. with n.s.) if testing was performed, but the differences are not significant. In case n=2, please show the data as separate datapoints without error bars and statistics. See also: http://www.embopress.org/page/journal/14693178/authorguide#statisticalanalysis

If n<5, please show single datapoints for diagrams.

9) Please confirm that for all Western blot panels (main and EV figures) the loading control was run on the same gel as the other proteins detected. Please note that we discourage comparisons between samples on different gels/blots, even if the samples derive from one experiment, as confounding factors reduce comparability. If unavoidable, the figure legend must state that the samples derive from the same experiment and that gels/blots were processed in parallel. If a 'representative' loading control is shown for multiple gels/blots, the intra-gel controls should be shown in the source data files, and the figure legends should describe the data displayed accurately. See our author guidelines:

https://www.embopress.org/page/journal/14693178/authorguide#datapresentationformat (section 'Electrophoretic gels and blots').

and

https://www.embopress.org/image-integrity

10) Please also note our reference format:
http://www.embopress.org/page/journal/14693178/authorguide#referencesformat

11) We updated our journal's competing interests policy in January 2022 and request authors to consider both actual and perceived competing interests. Please review the policy https://www.embopress.org/competing-interests and update your competing interests if necessary. Please name this section 'Disclosure and Competing Interests Statement' and put it after the Acknowledgements section.

12) We now use CRediT to specify the contributions of each author in the journal submission system. CRediT replaces the author contribution section. Please use the free text box to provide more detailed descriptions and do NOT provide your final manuscript text file with an author contributions section. See also our guide to authors:
https://www.embopress.org/page/journal/14693178/authorguide#authorshipguidelines

13) All Materials and Methods need to be described in the main text using our 'Structured Methods' format, which is required for all research articles. According to this format, the Methods section should include a Reagents and Tools Table (listing key reagents, experimental models, software, and relevant equipment and including their sources and relevant identifiers), uploaded as separate file, and a Methods section in which we encourage the authors to describe their methods using a step-by-step protocol format with bullet points, to facilitate the adoption of the methodologies across labs. More information on how to adhere to this format as well as downloadable templates (.doc) for the Reagents and Tools Table can be found in our author guidelines (section 'Structured Methods'):

https://www.embopress.org/page/journal/14693178/authorguide#manuscriptpreparation

14) Please order the manuscript sections like this, using only these names:
Title page - Abstract (max. 175 words) - Keywords - Introduction - Results - Discussion - Methods - Data availability section - Acknowledgements (please include here all the funding information) - Disclosure and Competing Interests Statement - References - Figure legends - Expanded View Figure legends

15) Please make sure that all the funding information is also entered into the online submission system and that it is complete and similar to the one in the acknowledgement section of the manuscript text file.

We would also welcome the submission of cover suggestions, or motifs to be used by our Graphics Illustrator in designing a cover.

As part of the EMBO publication's Transparent Editorial Process, EMBO reports publishes online a Review Process File (RPF) to accompany accepted manuscripts. This File will be published in conjunction with your paper and will include the referee reports, your point-by-point response and all pertinent correspondence relating to the manuscript.

You are able to opt out of this by letting the editorial office know (emboreports@embo.org). If you do opt out, the Review Process File link will point to the following statement: "No Review Process File is available with this article, as the authors have chosen not to make the review process public in this case."

I look forward to seeing a revised form of your manuscript when it is ready.

Yours sincerely,

Achim Breiling
Senior Editor
EMBO Reports

Referee #1:

This study by Belbelazi et al. presents a potentially significant and novel mitotic function for PKMYT1, expanding beyond its well-established role as a G2/M regulator. The authors propose that PKMYT1 is crucial for maintaining spindle assembly checkpoint (SAC) integrity and ensuring accurate chromosome segregation, acting in concert with Cyclin B1 degradation to fine-tune CDK1-Cyclin B1 activity during the metaphase-to-anaphase transition. Their findings suggest that PKMYT1 loss disrupts the fidelity of this transition, leading to nuclear atypia and genomic instability.

While the manuscript offers intriguing insights on the PKMYT1 role in cell cycle regulation, the precise mechanisms by which PKMYT1 modulates intra-mitotic functions remain quite unclear. With this in mind, it is crucial that approaches and experimental controls are of high quality. The data presented are generally well conducted and presented, however, several key claims necessitate additional supporting evidence to strengthen the authors conclusions.

Major Points:

1. Reliance on cell cycle block-and-release experiments: The authors reliance on cell cycle inhibitory block-and-release experiments, often involving multiple sequential blocks and releases, raises concerns about potential artifacts inherent to the experimental setup. To address this, the conclusions in Extended Figure 1C-D (accelerated mitotic exit after PKMYT1 inhibition) must be supported by additional evidence. The observed effect size is modest, and inhibitors are never fully specific. Essential controls include repeating these experiments using the RPE-1 PKMYT1 knockout cells and including RP-6306 in both WT and KO background as a control for inhibitor specificity.
2. Need for validation in PKMYT1 knockout cells: Similar to point #1, interpretations based on Figure 2 (D-G, and L, N-O) rely on experiments that should be rigorously validated in PKMYT1 knockout cells, both with and without RP-6306 treatment, to eliminate potential off-target effects and confirm the direct role of PKMYT1.
3. Discriminating G2 vs. mitotic inhibition effects: The manuscript needs to explicitly differentiate between the effects of PKMYT1 inhibition in late G2 phase versus in mitosis itself. In the current experimental design (Figures 3 and 4), where inhibitors are applied before cells enter mitosis, it is unclear whether the observed genomic instability and micronuclei formation result from a direct role of PKMYT1 in mitosis or from premature mitotic entry due to G2 inhibition (owing to issue such as unrepaired DNA damage or an unsatisfied decatenation checkpoint). To address this, the authors should for example use their imaging data to perform in silico synchronization, tracking only cells that have already entered mitosis at the time of inhibitor addition. This would allow for a more precise assessment of PKMYT1's mitotic function.
4. Limited analysis of biological impact: The authors should provide additional evidence for the biological impact of PKMYT1 ablation. Specifically, are PKMYT1-deficient RPE-1 cells more sensitive to SAC- or spindle-targeting compounds compared to wild-type RPE-1 cells? This would provide further support for a direct role of PKMYT1 in SAC function.
5. Need for mechanistic exploration in discussion: While the authors are acknowledged experts in the cell cycle field, the mechanistic explanation of PKMYT1's intra-mitotic functions in the discussion is convoluted and must be improved. The authors need to reconcile the seemingly contradictory observations regarding CDK1-Cyclin B1 activity (PKMYT1 blocks CDK1 in G2 phase, but supports intra-mitotic CDK1 activity). The authors do suggest a direct role of PKMYT1 activity to promote CDK1-Cyclin B1 activity in the main text related to figure 2. Perhaps recognizing the difficulty of solving all functional-mechanistic aspects in a single manuscript, they should discuss potential functional-mechanistic explanations. Thus, the discussion section should more clearly articulate how PKMYT1 mechanistically can promote CDK1-Cyclin B1 activity.

Minor points:
6. Limited evidence for "essential role": The authors claim an "essential role" for PKMYT1, but lack direct evidence that PKMYT1 activity specifically in mitosis is required for cell viability. Consider rephrasing this claim to reflect the observed effects on SAC integrity and chromosome segregation, rather than an absolute requirement for cell survival. Potentially, data related to point #4 above may support this claim.
7. Claim regarding CDK1 phosphorylation: The manuscript states that PKMYT1 modifies both Tyr15/Thr14 of CDK1. Recent studies using RP-6306 (e.g., Gallo et al., 2022; Benada et al., 2023) suggest that PKMYT1 primarily phosphorylates Thr14 in human cells. Did the authors observe any impact on Tyr15 phosphorylation using PKMYT1 inhibitors or the PKMYT1 degron in mitosis? This discrepancy should be addressed.
8. Missing data on knockout efficiency: The PKMYT1 knockout efficiency for all three clones used in Extended Figures 1A and B should be explicitly presented to ensure the validity of the experimental results.
9. Limited analysis of cGAS-STING pathway activation: To strengthen the link between PKMYT1 inhibition and genomic instability, the authors could include data on cGAS and p-STAT3 detection by western blot and/or immunofluorescence to provide more direct evidence for cGAS-STING pathway activation.
10. Lack of DNA Damage marker analysis: The inclusion of a DNA damage marker (e.g., γH2AX or 53BP1) alongside the micronuclei readout would provide further support for the authors' claim that PKMYT1 promotes genome stability.
11. Missing information on RP-6306 concentration: The concentration of RP-6306 used in Extended Figure 1C should be included in the figure legend for clarity and reproducibility.

Referee #2:

In this manuscript, Belbelazi et al. investigate the function of the Cdk1-inhibitory kinase PKMYT1 beyond its canonical role at the G2-M transition. They report that the enzymatic activity of PKMYT1 increases by dephosphorylation in late mitosis and is required for the proper function of the spindle assembly checkpoint. Consequently, depletion or chemical inhibition of PKMYT1 results in premature anaphase and chromosome missegregation, followed by micronuclei formation and activation of cGAS.

The authors convincingly demonstrate by clear-cut data that PKMYT1 is activated in late mitosis and that PKMYT1 is required for faithful chromosome segregation. However, as outlined below, other statements of the manuscript are not sufficiently supported by the data presented.

Major points of criticism:
The authors posit that anaphase occurs prematurely in the absence of PKMYT1 (activity). Nevertheless, the corresponding data are not very compelling: At 40 min the cell cycle profile in Extended Fig. 1C not only shows a smaller G2/M peak for RP6306-treated cells; the G1 peak is smaller, too. And why is the time between metaphase and anaphase not discernibly shorter for RP6306-treated cells in Figs. 1D/E?

Similarly, the weakening of the SAC upon chemical inhibition of PKMYT1 is little convincing. As illustrated in Figure 4D, the average length of mitosis is only marginally shortened by the addition of RP6306 to taxol-treated cells.
Even less convincing are the Western blots based on which the authors conclude that Cdk1 inactivation is accelerated in absence of PKMYT1 activity (Fig. 2L). The purported "start contrast" between the RP6306 treatment and the DMSO control is not discernible. Moreover, the kinetics do not fit: According to Fig. 1, the activation of PKMYT1 by dephosphorylation only begins 30 min after release from nocodazole. How then can the addition of RP6306 have an effect on the dephosphorylation of Cdk1 substrates as early as 3 min after the wash-out of nocodazole (Fig. 2)?

Given the canonical role of PKMYT1, it is counterintuitive and and confusing that absence of this kinase should result in accelerated inactivation of Cdk1-cyclin B1. Yet, the authors do not address this issue in the entire manuscript. At least in the discussion they should speculate about a potential mechanism that could provide an explanation for their observation.

Minor points:
Why was the phospho-S126 cyclin B1 antibody not included in figure 2?
PKMYT1 is a dual specific kinase that also phosphorylates Y15 of Cdk1. Why then was the assessment of this inhibitory phosphorylation not included in figure 2L?
On page 8 the authors write: "Although cells treated with either inhibitor appeared to progress through mitosis with no apparent delays,..." This contradicts the claim that inhibition of PKMYT1 or Wee1 results in shortening or extension of late mitosis, respectively.

Another contradiction: On page 8 the authors write: "This observation suggests that inhibition of either WEE1 or PKMYT1 may compromise the SAC, allowing anaphase to proceed despite incorrect chromosome attachment, and it has been previously reported that WEE1 activity is required for SAC strength (Visconti et al., 2015)." Yet, on the next page they write: "Consistent with previous literature, the WEE1 inhibitor prolonged mitotic arrest compared to the DMSO control."

Page 5 (middle): ...from 30 to 55 minutes... (not 30 to 60).

Flow cytometry of PI stained cells cannot "confirm" an accumulation at the end of G2 phase, as stated on page 7 (middle).

Comments from Referee 1:

Major Points:

1. Reliance on cell cycle block-and-release experiments: The authors reliance on cell cycle inhibitory block-and-release experiments, often involving multiple sequential blocks and releases, raises concerns about potential artifacts inherent to the experimental setup. To address this, the conclusions in Extended Figure 1C-D (accelerated mitotic exit after PKMYT1 inhibition) must be supported by additional evidence. The observed effect size is modest, and inhibitors are never fully specific. Essential controls include repeating these experiments using the RPE-1 PKMYT1 knockout cells and including RP-6306 in both WT and KO background as a control for inhibitor specificity.

We thank the referee for this constructive comment.

We agree that it is essential to ensure that the mitotic acceleration phenotype does not stem from off-target effects of the PKMYT1 inhibitor (RP 6306). To address this, we repeated the experiments shown in Extended Figure 1C-D using a more refined synchronization protocol. We employed sequential treatment with CDK4/6 and CDK1 inhibitors to accumulate cells at the G2/M boundary. We then released both wild-type (WT) and PKMYT1−/− cells into mitosis, adding the PKMYT1 inhibitor at the moment of release. We observed that RP-6306 accelerated mitotic progression in WT cells, whereas no acceleration was observed in PKMYT1−/− cells. This lack of an additive effect in the knockout background confirms the specificity of the inhibitor in promoting mitotic acceleration. These data are now presented in Appendix Fig S. 2 and discussed on page 4.

Furthermore, to confirm the specificity of the drug without pre-synchronization— and to decouple the mitotic function of PKMYT1 from its role in late G2—we performed single-cell live imaging. In these experiments, the inhibitor was added specifically to cells already in prometaphase, and we measured the duration from prometaphase to anaphase onset. Consistent with our other findings, adding the PKMYT1 inhibitor during prometaphase accelerated mitotic progression in WT cells, whereas no acceleration was observed in PKMYT1−/− cells. These data are presented in Appendix Fig S. 3 and discussed on pages 4 and 5.

2. Need for validation in PKMYT1 knockout cells: Similar to point #1, interpretations based on Figure 2 (D-G, and L, N-O) rely on experiments that should be rigorously validated in PKMYT1 knockout cells, both with and without RP-6306 treatment, to eliminate potential off-target effects and confirm the direct role of PKMYT1.

We thank the referee for this insightful observation.

As shown in Appendix Fig S 2 and 3, the evidence suggests that the acceleration of mitotic progression is a specific consequence of PKMYT1 loss. To further ensure that our phenotypic markers were not affected by off-target activity, we examined the timing and pattern of CPC relocalization and NEBD in an asynchronous setting using PKMYT1−/− cells. We observed that the PKMYT1 inhibitor had no impact on the kinetics or localization of these markers in the knockout background (Appendix Fig S 4). This confirms that the premature relocalization of the CPC—

which we used as a primary indicator of anaphase onset—is specifically linked to PKMYT1 inhibition and does not stem from off-target effects of the inhibitor.

The Results section has been updated on page 6 to include these data.

3. Discriminating G2 vs. mitotic inhibition effects: The manuscript needs to explicitly differentiate between the effects of PKMYT1 inhibition in late G2 phase versus in mitosis itself. In the current experimental design (Figures 3 and 4), where inhibitors are applied before cells enter mitosis, it is unclear whether the observed genomic instability and micronuclei formation result from a direct role of PKMYT1 in mitosis or from premature mitotic entry due to G2 inhibition (owing to issue such as unrepaired DNA damage or an unsatisfied decatenation checkpoint). To address this, the authors should for example use their imaging data to perform in silico synchronization, tracking only cells that have already entered mitosis at the time of inhibitor addition. This would allow for a more precise assessment of PKMYT1's mitotic function.

We appreciate the referee highlighting the need to distinguish between PKMYT1 activity in late G2 and mitosis. This allowed us to clarify our model and provide more precise evidence of its mitotic role.

We have added several lines of evidence to demonstrate that the observed genomic instability is a direct result of mitotic PKMYT1 deficiency.

1. Mitotic DNA Damage Analysis: We have added Figure EV. 2, which shows that inhibition of either PKMYT1 or WEE1 specifically during mitosis increases DNA damage. By using a nocodazole shake-off protocol to isolate mitotic cells before drug treatment, we ensured that the observed increase in γH2AX staining occurs independently of any G2 carry-over effects. This is discussed on page 8.
2. Validation via Acute Protein Depletion: To further isolate the mitotic requirements of PKMYT1, we used the auxin-inducible degron (AID) system to acutely deplete PKMYT1 in nocodazole-arrested cells. Upon release, we observed a significant increase in the formation of micronuclei. Because the protein was depleted only after the cells had already entered mitosis, this confirms that the genomic instability is specific to PKMYT1's mitotic function and is not a G2 artifact. These data are shown in Figure EV. 4 and described on page 9.
3. Live-Cell Tracking of Prometaphase Cells: Regarding the results in Figure 4, we have clarified in the text that this analysis was conducted specifically on cells exposed to the drug during mitosis. Only cells that were already in prophase at the start of imaging/treatment were included in the analysis. This ensures the observed phenotypes are not a result of premature mitotic entry. The text has been updated on pages 9 and 10 to make this methodology explicit.

We would like to thank the referee once again for highlighting these points, which have significantly improved the clarity and impact of our manuscript.

4. Limited analysis of biological impact: The authors should provide additional evidence for the biological impact of PKMYT1 ablation. Specifically, are PKMYT1-deficient RPE-1 cells more sensitive to SAC- or spindle-targeting compounds compared to wild-type RPE-1 cells? This would provide further support for a direct role of PKMYT1 in SAC function.

We thank the referee for the suggestion to further explore the biological impact of PKMYT1 loss on the SAC.

As our study is presented as a Short Report, we focused on providing a foundational characterization of PKMYT1's mitotic role. To address the reviewer's query, we have added Appendix Fig S. 6 to the revised manuscript. While the acceleration of mitotic slippage was established in our primary figures, this new evidence demonstrates that cells escaping mitotic arrest under PKMYT1 inhibition maintain a tubulin structure similar to the control cells (DMSO). This structural preservation is key; it demonstrates that the accelerated exit is not a secondary result of microtubule instability or spindle damage. Instead, it suggests that PKMYT1 specifically contributes to the robustness of SAC-mediated arrest.

While these data demonstrate that PKMYT1 is required for full SAC robustness, we have modified the main text to explicitly state that while PKMYT1 contributes to SAC strength, it is not essential for its primary activation. We agree that a more detailed dissection of the specific molecular pathway involved will be an important area for future study.

5. Need for mechanistic exploration in discussion: While the authors are acknowledged experts in the cell cycle field, the mechanistic explanation of PKMYT1's intra-mitotic functions in the discussion is convoluted and must be improved. The authors need to reconcile the seemingly contradictory observations regarding CDK1-Cyclin B1 activity (PKMYT1 blocks CDK1 in G2 phase, but supports intra-mitotic CDK1 activity). The authors do suggest a direct role of PKMYT1 activity to promote CDK1-Cyclin B1 activity in the main text related to figure 2. Perhaps recognizing the difficulty of solving all functional-mechanistic aspects in a single manuscript, they should discuss potential functional-mechanistic explanations. Thus, the discussion section should more clearly articulate how PKMYT1 mechanistically can promote CDK1-Cyclin B1 activity.

We thank the referee for this thoughtful critique.

We agree that the dual role of PKMYT1—acting as a biochemical inhibitor of CDK1 during G2 while appearing to support CDK1-mediated mitotic functions— is counterintuitive and requires clarification, along with a potential explanation for this phenotype.

To address this, we have added a dedicated section to the Discussion (pages 11–12) that reconciles these observations. We now propose that PKMYT1 modulates the activity of the remaining CDK1-Cyclin B1 pool during the metaphase-to-anaphase transition. In this model, the loss of PKMYT1 leads to the hyper-activation of CDK1-Cyclin B1, which in turn accelerates negative feedback loops. This explains

the counterintuitive result where inhibiting mitotic PKMYT1 ultimately leads to accelerated mitotic exit. We also suggest an alternative explanation, that PKMYT1 could regulate CDH1, the APC/C co-activator that replaces CDC20 as cells exit mitosis and is not subject to inhibition by the SAC.

Minor points:

6. Limited evidence for "essential role": The authors claim an "essential role" for PKMYT1, but lack direct evidence that PKMYT1 activity specifically in mitosis is required for cell viability. Consider rephrasing this claim to reflect the observed effects on SAC integrity and chromosome segregation, rather than an absolute requirement for cell survival. Potentially, data related to point #4 above may support this claim.

We thank the referee for the suggestion to refine our terminology.

We agree that our data specifically demonstrates a requirement for PKMYT1 in ensuring the accuracy of mitotic events rather than absolute cell viability. We have updated the manuscript to state that PKMYT1 is required for fidelity of mitotic progression and that its loss leads to genomic instability. We have rephrased these claims in the main text to more accurately reflect the observed phenotypes.

7. Claim regarding CDK1 phosphorylation: The manuscript states that PKMYT1 modifies both Tyr15/Thr14 of CDK1. Recent studies using RP-6306 (e.g., Gallo et al., 2022; Benada et al., 2023) suggest that PKMYT1 primarily phosphorylates Thr14 in human cells. Did the authors observe any impact on Tyr15 phosphorylation using PKMYT1 inhibitors or the PKMYT1 degron in mitosis? This discrepancy should be addressed.

We thank the referee for highlighting the substrate specificity of PKMYT1.

While PKMYT1 has the biochemical capacity to phosphorylate both T14 and Y15, we agree that its primary physiological role in human cells is directed toward T14.

To address this, we have included western blot analysis in Appendix Fig S. 1C, which demonstrates that the genetic loss of PKMYT1 significantly reduces the phospho-T14 signal while leaving phospho-Y15 levels largely unaffected when WEE1 is active. This confirms that in our system, PKMYT1 is the primary kinase for T14, consistent with recent reports (Gallo et al., 2022; Benada et al., 2023). The Results section on page 4 has been updated to reflect these findings.

8. Missing data on knockout efficiency: The PKMYT1 knockout efficiency for all three clones used in Extended Figures 1A and B should be explicitly presented to ensure the validity of the experimental results.

We thank the referee for noting the importance of verifying knockout efficiency.

We have now included this validation in Appendix Fig S. 1C. We performed Western blot analysis using two different PKMYT1 antibodies across all three knockout

clones. The results clearly demonstrate the complete loss of PKMYT1 protein. Furthermore, we show that the loss of PKMYT1 leads to a significant reduction in CDK1 phospho-T14 levels compared to wild-type cells, further confirming the functional ablation of the kinase. These data are now presented in Appendix Fig S. 1C, and the corresponding text has been updated on page 4.

9. Limited analysis of cGAS-STING pathway activation: To strengthen the link between PKMYT1 inhibition and genomic instability, the authors could include data on cGAS and p-STAT3 detection by western blot and/or immunofluorescence to provide more direct evidence for cGAS-STING pathway activation.

We thank the referee for the suggestion to provide more direct evidence of cGAS-STING activation. While we agree that this pathway is a critical consequence of PKMYT1 loss, the primary focus of this Short Report is the mitotic function of PKMYT1 and of genomic instability.

We have already provided functional evidence of this inflammatory signaling through RT-qPCR analysis showing an increase in IL-6 expression following PKMYT1 inhibition. Given that the formation of micronuclei (the physical trigger for cGAS) is clearly documented in our data, we believe the IL-6 data sufficiently demonstrates the biological consequence of this instability. Therefore, we have opted to keep the manuscript focused on the upstream mitotic mechanisms rather than expanding into a full characterization of the STING signaling axis, which we believe falls outside the scope of this focused report.

10. Lack of DNA Damage marker analysis: The inclusion of a DNA damage marker (e.g., γH2AX or 53BP1) alongside the micronuclei readout would provide further support for the authors' claim that PKMYT1 promotes genome stability.

We thank the referee for this suggestion.

We agree that direct evidence of DNA damage is essential to support our claims regarding genomic stability. As we previously mentioned, we have addressed this by performing immunofluorescence analysis of the DNA double-strand break marker γH2AX. As shown in the new Fig EV. 2, inhibition of either PKMYT1 or WEE1 results in a significant increase in the number of γH2AX foci per nucleus. These results provide direct evidence that PKMYT1 activity is required to prevent DNA damage during mitosis. We have updated the Results section (page 8) to include this new data.

11. Missing information on RP-6306 concentration: The concentration of RP-6306 used in Extended Figure 1C should be included in the figure legend for clarity and reproducibility.

We thank the referee for ensuring the reproducibility of our data by requesting these details. We have now added the concentration of RP-6306 used in the experiments to the figure legends.

Comments from Referee 2:

Major Points:

1. The authors posit that anaphase occurs prematurely in the absence of PKMYT1 (activity). Nevertheless, the corresponding data are not very compelling: At 40 min the cell cycle profile in Extended Fig. 1C not only shows a smaller G2/M peak for RP6306-treated cells; the G1 peak is smaller, too. And why is the time between metaphase and anaphase not discernibly shorter for RP6306-treated cells in Figs. 1D/E?

We thank the referee for this insightful evaluation of our initial timing data.

We agree that the prolonged exposure (24 hr) to the CDK1i inhibitor in the original experiment may have introduced confounding variations in the cell cycle profile. To improve the clarity of these profiles, we implemented a more refined synchronization protocol using sequential CDK4/6 inhibition followed by a short-duration exposure to the CDK1i. This approach minimizes the time cells spend arrested at the G2/M boundary and prevents systemic toxicity. As shown in the updated Appendix Fig S. 2, this protocol resulted in significantly cleaner FACS profiles with balanced G1 and G2/M populations. These new results consistently demonstrate that PKMYT1 inhibition accelerates the 4N-to-2N transition. We have replaced the previous FACS data with these new results.

Regarding the second point, the original Figure 1 was intended primarily to demonstrate that mitotic PKMYT1 is dephosphorylated during mitosis, reflecting an increase in its activity as evidenced by T14 phosphorylation on CDK1. We acknowledge that this specific experiment was not optimized to capture kinetic acceleration; the use of nocodazole to arrest cells can introduce variability in microtubule assembly kinetics that can mask differences in mitotic timing.

To provide definitive evidence of acceleration, we performed live-cell imaging on asynchronous cells to measure the natural duration of mitosis from prometaphase to anaphase onset. As shown in the new Appendix Fig S. 3, treatment with RP 6306 significantly shortens the duration of mitosis compared to DMSO controls. The text on page 4-5 has been updated to reflect these improved experimental methodologies and the resulting data.

2. Similarly, the weakening of the SAC upon chemical inhibition of PKMYT1 is little convincing. As illustrated in Figure 4D, the average length of mitosis is only marginally shortened by the addition of RP6306 to taxol-treated cells. Even less convincing are the Western blots based on which the authors conclude that Cdk1 inactivation is accelerated in absence of PKMYT1 activity (Fig. 2L). The purported "start contrast" between the RP6306 treatment and the DMSO control is not discernible. Moreover, the kinetics do not fit: According to Fig. 1, the activation of PKMYT1 by dephosphorylation only begins 30 min after release from nocodazole. How then can the addition of RP6306 have an effect on the dephosphorylation of Cdk1 substrates as early as 3 min after the wash-out of nocodazole (Fig. 2)?

We thank the reviewer for these observations, which have helped us clarify the distinction between PKMYT1 and other cell cycle regulators and identify a labelling error in our initial submission.

1. Regarding the Spindle Assembly Checkpoint (SAC) and Mitotic Slippage:

The reviewer is correct that the acceleration of mitotic exit in the presence of Paclitaxel is marginal compared to "core" SAC components like MPS1. In the revised manuscript, we have updated our phrasing to state that PKMYT1 contributes to SAC maintenance rather than being a "crucial" or "essential" component. To rule out structural interference, we added Appendix Fig S. 6A, which shows that cells treated with the combination of Paclitaxel and RP 6306 maintain a tubulin pattern similar to Paclitaxel alone. This indicates that PKMYT1 influences the SAC signalling threshold rather than disrupting microtubule structures directly.

2. Clarification of "Stark Contrast":

We have revised the text to replace "stark contrast" with "in contrast." This change reflects our intended meaning: that the phenotype of PKMYT1 inhibition during metaphase is qualitatively different from that of WEE1 inhibition, rather than claiming a massive shift in absolute kinetics.

3. Correction of the Washout Protocol:

We apologize for a labeling error in the Western Blots in Figure 2. The experiment was performed using an APC/C inhibitor (APC/Ci) washout, not a nocodazole washout.

This distinction is vital for the kinetics:

- Nocodazole washout requires significant time for cells to reassemble microtubules and align chromosomes on the metaphase plate.
- APC/Ci washout involves cells that are already aligned at metaphase and are "primed" for segregation.

Upon the removal of the APC/Ci, the cells are ready to immediately segregate chromosomes. This explains why the drop in CDK1 activity is observable as early as 3 minutes post-washout. We have corrected the labels in Figure 2L.

We thank the referee once again for these insightful comments; addressing them has significantly strengthened the clarity and technical accuracy of our manuscript.

3. Given the canonical role of PKMYT1, it is counterintuitive and and confusing that absence of this kinase should result in accelerated inactivation of Cdk1-cyclin B1. Yet, the authors do not address this issue in the entire manuscript. At least in the discussion they should speculate about a potential mechanism that could provide an explanation for their observation.

We thank the referee for this note.

We agree that the accelerated inactivation of CDK1 upon PKMYT1 inhibition is a counterintuitive phenotype given PKMYT1's canonical role as an inhibitory kinase. We have added a paragraph to the Discussion highlighting this observation and providing potential mechanistic explanations.

Minor points:

4. Why was the phospho-S126 cyclin B1 antibody not included in figure 2?

We recognize that the choice of markers is critical for interpreting mitotic exit kinetics. Due to the limited number of antibodies available per slide, we strategically chose two high-value markers to comprehensively assess the impact of inhibiting PKMYT1 or WEE1 on later mitotic stages: first, CPC (Chromosomal Passenger Complex) relocalization, which serves as a highly sensitive and temporally precise marker for the Metaphase-to-Anaphase transition; and second, Nuclear Envelope Reformation (NER), an event tightly controlled by the rapid drop in CDK1 activity as the cell exits mitosis.

5. PKMYT1 is a dual specific kinase that also phosphorylates Y15 of Cdk1. Why then was the assessment of this inhibitory phosphorylation not included in figure 2L?

We thank the referee for this note.

From the biochemical characterization of the PKMYT1−/− clones (added as a new panel in Appendix Fig S. 1C), western blot analysis shows that while PKMYT1 is depleted, Y15 phosphorylation remains comparable to wild-type (WT) levels, whereas the T14 signal is significantly reduced. This observation is consistent with several previous studies indicating that, while PKMYT1 is a dual-specificity kinase, it preferentially phosphorylates T14 on CDK1 in a cellular context.

Based on this clear evidence that T14 is the primary site sensitive to PKMYT1 status in our model, we utilized the T14 phosphorylation signal as an indicator of PKMYT1 activity.

6. On page 8 the authors write: "Although cells treated with either inhibitor appeared to progress through mitosis with no apparent delays,..." This contradicts the claim that inhibition of PKMYT1 or Wee1 results in shortening or extension of late mitosis, respectively.

We thank the referee for identifying this inconsistency.

Our original phrasing was intended to convey that the treatment did not result in a mitotic stall or failure to exit. To ensure alignment with our kinetic data, we have revised the text on page 9 to read:
"Despite apparently typical progression through mitosis, we observed that..."

7. Another contradiction: On page 8 the authors write: "This observation suggests that inhibition of either WEE1 or PKMYT1 may compromise the SAC, allowing anaphase to proceed despite incorrect chromosome attachment, and it has been previously reported that WEE1 activity is required for SAC strength (Visconti et al., 2015)." Yet, on the next page they write: "Consistent with previous literature, the WEE1 inhibitor prolonged mitotic arrest compared to the DMSO control."

We thank the referee for spotting this inconsistency in our initial interpretation.

We have revised the text on page 9 to more accurately reflect the discrete regulatory functions of these kinases in mitotic regulation. The updated text now states:
"These observations indicate that PKMYT1 is required for SAC-mediated regulation, as its inhibition allows anaphase to proceed despite improper chromosome attachments. While WEE1 has been previously reported to contribute to SAC strength (Visconti et al., 2015), our findings indicate that PKMYT1 may also play a role in governing the timing of this transition."

8. Page 5 (middle): ...from 30 to 55 minutes... (not 30 to 60).

We thank the referee for identifying this discrepancy. We have corrected the text on page 5 to reflect the accurate time interval of 30 to 55 minutes.

9. Flow cytometry of PI stained cells cannot "confirm" an accumulation at the end of G2 phase, as stated on page 7 (middle).

We agree with the referee that PI staining alone identifies DNA content but does not definitively distinguish between late G2 and M phases. We have modified the text on page 8 to more accurately state:"...as indicated by FACS analysis,".

Dear Dr. Belbelazi,

Thank you for the submission of your revised manuscript to our editorial offices. I have now received the reports from the two referees that I asked to re-evaluate the study, you will find below. As you will see, the referees now fully support the publication of your study in EMBO reports.

Before we can proceed with formal acceptance, I have the editorial requests below I ask you to address in a final revised manuscript. Please also provide a final p-b-p-response to the editorial requests.

Editorial requests:

- There is an author name discrepancy: It is Eleanor Wendy Trotter on the title page vs. Eleanor Trotter in the submission system. Please check and make sure the exact same name is used in the final submission.

- We will publish your manuscript as Report (as also indicated by you in the submission system). For a Scientific Report we require that results and discussion sections are combined in a single chapter called "Results & Discussion". Please do this for your manuscript. For more details, please refer to our guide to authors:

https://link.springer.com/journal/44319/submission-guidelines#cms-Article-types

- We do not allow figures with more than one page (Figure 2 in this submission). Moreover, there are two figures 4. Please rearrange the figures to have 5 final one paged main figures. Then please update all the callouts.

- Please make sure that the nomenclature for the figures and their callouts are uniform throughout the manuscript text and the legends: Figure Xy, Figure EVx and Appendix Figure Sx. Please do not add spaces or points, except you want to shorten the word figure (Fig.).

- Please order the manuscript sections like this, using only these names:
Title page - Abstract - Keywords - Introduction - Results & Discussion - Methods - Data availability section - Acknowledgements (including the funding information) - Disclosure and Competing Interests Statement - References - Figure legends - Expanded View Figure legends

- Please check again that the number "n" for how many independent experiments were performed, their nature (biological versus technical replicates), the bars and error bars (e.g. SEM, SD) and the test used to calculate p-values is indicated in the respective figure legends (main and EV figures). Please also check that all the p-values are explained in the legend, and that these fit to those shown in the figure. Please provide statistical testing where applicable. Please avoid the phrase 'independent experiment' but clearly state if these were biological or technical replicates. Please also indicate (e.g. with n.s.) if testing was performed, but the differences are not significant. In case n=2, please show the data as separate datapoints without error bars and statistics. See also:

https://link.springer.com/journal/44319/submission-guidelines#cms-Figure-and-data-presentation

If n<5, please show single datapoints for diagrams. Moreover:
- Please note that the exact p values are not provided in the legends of figures 1H, I; 2D, G, J, K; 3D, E, G; 3D, E, G; 4B, D; EV1B; EV2C, EV4C
- Please note that the error bars are not defined in the legend of figure EV3B.

- Please use our reference format:
https://link.springer.com/journal/44319/submission-guidelines#cms-Reference-guidelines

- There are 11 movies uploaded, they all play, but their legends are missing. Each movie should have a legend provided as a text file and then ZIPed up with its movie file so that we have 11 separate zip folders. The nomenclature for movies is 'Movie EVx'. Please use this for filenames and callouts. Presently, the file names of the movie files have figure panel names. Are these rather source data files?

- Please make sure that each final figure panel (of main, EV and Appendix figures) is called out separately and that the panels are called out sequentially. Presently, there is no callout for Figure EV2. Please check. Moreover, 'Video 1' and 'Video 2' is called out. Are these callouts for movies? Please use the nomenclature 'Movie EVx' and provide callouts for all 11 movies.

- Please provide the Appendix file as PDF. The ToC needs to have the full names of each figure: "Appendix Figure Sx", instead of "Sx". Please also add a title to the first page of the Appendix ("Appendix for ..." - followed by the title of the paper - please do not add author names or affiliations).

- Please make sure that different microscopic images in composite panels are clearly separated by white lines (like in panels EV1C or Appendix Fig. S1A and S5B - these three are fine). Presently, images in panels 1J, 2B/C, 4A/B, EV2B, S3A, S4A/B and S6A are not well separated. Please fix this.

- Please confirm that for all Western blot panels the lanes shown (including the loading control) were run on the same gel as the other proteins detected. I am not sure this is always the case. Please note that we discourage comparisons between samples on different gels/blots, even if the samples derive from one experiment, as confounding factors reduce comparability. If unavoidable, the figure legend must state that the samples derive from the same experiment and that gels/blots were processed in parallel. If a 'representative' loading control is shown for multiple gels/blots, the intra-gel controls should be shown in the source data files, and the figure legends should describe the data displayed accurately. See our author guidelines:

https://link.springer.com/journal/44319/submission-guidelines#cms-Figure-and-data-presentation (section 'Electrophoretic gels and blots').

- We noted an image reuse between Figure 4C and Figure 4B Video. Please indicate this in the respective figure legend. I guess one is showing the full image that you then took the insert from?

Moreover, the resolution of the submitted figures is too low for blot and microscopy images. This reduction in resolution is commonly caused by converting original 16-bit TIFF files to RGB format for publication. While this is not inherently problematic, it can raise concerns about image integrity for critical readers.

To avoid any misunderstanding and to meet EMBO Press standards, we kindly ask that you:

* Resubmit the complete figure set at the captured original data resolution.

* Apply the same resolution standards to the blot source data files, which are also currently below the required quality threshold. Please upload the blot source data files as .tiff or pdf. Please do not use PowerPoint.

- Thanks for providing the source data. Please upload the source data as one folder per final main figure, grouping together the separate files (in excel format) for the panels, and ZIPed together.

In addition, I would need from you uploaded separately:
- a short, two-sentence summary of the manuscript (not more than 35 words).
- two to four short (!) bullet points highlighting the key findings of your study (two lines each).
- a schematic summary figure as separate file that provides a sketch of the major findings (not a data image) in jpeg or tiff format (with the exact width of 550 pixels and a height of exactly 300 pixels) that can be used as a visual synopsis on our website.

I look forward to seeing the further revised version of your manuscript when it is ready.

Please let me know if you have questions regarding the revision.

Best,

Achim Breiling
Senior Editor
EMBO Reports

Referee #1:

The authors have addressed my revision points, and I have no further requests.

Referee #2:

In their revised manuscript Hagan and coworkers have adequately addressed all points previously raised by this referee. In doing so, they further improved the quality of their manuscript although the overall impact of PKMYT1 on the timing and fidelity of chromosome segregation appears rather small.

Dear Dr. Breiling,

Thank you for the opportunity to address the final editorial requirements for our manuscript. Below, we provide a point-by-point response detailing the modifications made.

Please do let me know if there is any further information required for the final processing of our manuscript.

Many Thanks,

Asma

Editorial requests:

- There is an author name discrepancy: It is Eleanor Wendy Trotter on the title page vs. Eleanor Trotter in the submission system. Please check and make sure the exact same name is used in the final submission.

We confirm that the correct full name is Eleanor Wendy Trotter, as listed on the manuscript title page. While we have attempted to update this in the submission system, the changes have not saved permanently. We would be grateful if the editorial office could ensure the system record is updated to match the title page for the final publication.

- We will publish your manuscript as Report (as also indicated by you in the submission system). For a Scientific Report we require that results and discussion sections are combined in a single chapter called "Results & Discussion". Please do this for your manuscript. For more details, please refer to our guide to authors:

https://link.springer.com/journal/44319/submission-guidelines#cms-Article-types [link.springer.com]

As requested, we have merged the Results and Discussion sections into a single chapter titled "Results and Discussion" to comply with the format requirements for a Scientific Report.

- We do not allow figures with more than one page (Figure 2 in this submission). Moreover, there are two figures 4. Please rearrange the figures to have 5 final one paged main figures. Then please update all the callouts.

We have restructured the figures as requested. The manuscript now contains five single-page main figures. All figure callouts within the text have been updated to reflect this new numbering.

- Please make sure that the nomenclature for the figures and their callouts are uniform throughout the manuscript text and the legends: Figure Xy, Figure EVx and Appendix

Figure Sx. Please do not add spaces or points, except you want to shorten the word figure (Fig.).

We have standardized the figure nomenclature throughout the manuscript.

- Please order the manuscript sections like this, using only these names: Title page - Abstract - Keywords - Introduction - Results & Discussion - Methods - Data availability section - Acknowledgements (including the funding information) - Disclosure and Competing Interests Statement - References - Figure legends - Expanded View Figure legends

We have reorganized the manuscript sections to follow the requested order and nomenclature.

- Please check again that the number "n" for how many independent experiments were performed, their nature (biological versus technical replicates), the bars and error bars (e.g. SEM, SD) and the test used to calculate p-values is indicated in the respective figure legends (main and EV figures). Please also check that all the p-values are explained in the legend, and that these fit to those shown in the figure. Please provide statistical testing where applicable. Please avoid the phrase 'independent experiment' but clearly state if these were biological or technical replicates. Please also indicate (e.g. with n.s.) if testing was performed, but the differences are not significant. In case n=2, please show the data as separate datapoints without error bars and statistics. See also:

https://link.springer.com/journal/44319/submission-guidelines#cms-Figure-and-data-presentation [link.springer.com]

If n<5, please show single datapoints for diagrams. Moreover:
- Please note that the exact p values are not provided in the legends of figures 1H, I; 2D, G, J, K; 3D, E, G; 3D, E, G; 4B, D; EV1B; EV2C, EV4C
- Please note that the error bars are not defined in the legend of figure EV3B.

We have audited all main and Expanded View figure legends to ensure full compliance with the data presentation guidelines. Specifically:

- We have replaced the phrase "independent experiments" with a clear indication of biological replicates.
- We have defined all error bars (e.g., mean ± SD or SEM) and identified the specific statistical tests used.
- Exact P values have been added to the Figures 1H, I; 2D, G, J, K; 3D, E, G; 4B, D; EV1B; EV2C, and EV4C.
- The error bars for Figure EV3B are now clearly defined in the legend.
-

- Please use our reference format: https://link.springer.com/journal/44319/submission-guidelines#cms-Reference-guidelines [link.springer.com]

We have updated the references to strictly adhere to the journal's guidelines. All citations in the text and the final reference list have been formatted accordingly.

- There are 11 movies uploaded, they all play, but their legends are missing. Each movie should have a legend provided as a text file and then ZIPed up with its movie file so that we have 11 separate zip folders. The nomenclature for movies is 'Movie EVx'. Please use this for filenames and callouts. Presently, the file names of the movie files have figure panel names. Are these rather source data files?

We have confirmed that these movies are indeed source data. They represent the full time-lapse sequences from which the representative snapshots in the figures were taken. Accordingly, they have been moved to the source data section. We have provided a corresponding text file for each movie to link the footage to the specific figure panels.

- Please make sure that each final figure panel (of main, EV and Appendix figures) is called out separately and that the panels are called out sequentially. Presently, there is no callout for Figure EV2. Please check. Moreover, 'Video 1' and 'Video 2' is called out. Are these callouts for movies? Please use the nomenclature 'Movie EVx' and provide callouts for all 11 movies.

We have updated the manuscript to ensure all figure panels (Main, EV, and Appendix) are called out separately. To maintain the narrative flow of the Results & Discussion while adhering to sequential formatting, we have introduced some panels for Figures 2, 3 and 4 in sequential groupings (e.g., Fig. 3A–J) at the beginning of the relevant sections. This ensures that every panel is introduced in alphabetical order upon its first mention. We have also added the missing callout for Fig. EV2.

Regarding the movies, we have determined that these files serve as source data (representing the full time-lapse sequences for the representative snapshots shown in the main figures) rather than standalone supplemental movies. Consequently, we have removed the 'Video' callouts from the main text and moved the movies to the source data section, accompanied by descriptive text files.

- Please provide the Appendix file as PDF. The ToC needs to have the full names of each figure: "Appendix Figure Sx", instead of "Sx". Please also add a title to the first page of the Appendix ("Appendix for ..." - followed by the title of the paper - please do not add author names or affiliations).

We have provided the Appendix as a PDF file. The first page now features the title "Appendix for PKMYT1 has an Important Role in the Timing and Fidelity of Chromosome Segregation" without author names or affiliations. Additionally, the Table of Contents has been updated to use the full nomenclature "Appendix Figure Sx" for all entries.

- Please make sure that different microscopic images in composite panels are clearly separated by white lines (like in panels EV1C or Appendix Fig. S1A and S5B - these three are fine). Presently, images in panels 1J, 2B/C, 4A/B, EV2B, S3A, S4A/B and S6A are not well separated. Please fix this.

We have revised all microscopic panels to ensure clear separation between individual images. Also, we have added distinct white borders/lines to panels 1J, 2B, 2C, 4A, 4B, EV2B, S3A, S4A, S4B, and S6A.

- Please confirm that for all Western blot panels the lanes shown (including the loading control) were run on the same gel as the other proteins detected. I am not sure this is always the case. Please note that we discourage comparisons between samples on different gels/blots, even if the samples derive from one experiment, as confounding factors reduce comparability. If unavoidable, the figure legend must state that the samples derive from the same experiment and that gels/blots were processed in parallel. If a 'representative' loading control is shown for multiple gels/blots, the intra-gel controls should be shown in the source data files, and the figure legends should describe the data displayed accurately. See our author guidelines:

https://link.springer.com/journal/44319/submission-guidelines#cms-Figure-and-data-presentation [link.springer.com] (section 'Electrophoretic gels and blots').

We confirm that for all Western blot panels, the protein of interest and its corresponding loading control were derived from the same experiment. The figure legends have been updated to explicitly state whether the loading control was processed on the same membrane or a parallel membrane to clearly demonstrate this to the reader. All lysates were pre-normalized prior to loading to ensure accurate comparability, and all gels were processed simultaneously under identical conditions. Full uncropped blots are provided in the Source Data for verification.

- We noted an image reuse between Figure 4C and Figure 4B Video. Please indicate this in the respective figure legend. I guess one is showing the full image that you then took the insert from?

We would like to clarify that the perceived "image reuse" occurred because the Figure 4B video was rendered as a static frame in the PDF. In the previous version, the first frame of the video appeared identical to the snapshot in Figure 5C because they were part of the same data series. To resolve this and avoid any further confusion, we have removed the video from the main figures and moved it to the Source Data section. Figure 5C now stands as the representative static panel, while the full motion sequence is available for review in the source data files.

Moreover, the resolution of the submitted figures is too low for blot and microscopy images. This reduction in resolution is commonly caused by converting original 16-bit TIFF files to RGB format for publication. While this is not inherently problematic, it can raise concerns about image integrity for critical readers.

To avoid any misunderstanding and to meet EMBO Press standards, we kindly ask that you:

* Resubmit the complete figure set at the captured original data resolution.

* Apply the same resolution standards to the blot source data files, which are also currently below the required quality threshold. Please upload the blot source data files as .tiff or pdf. Please do not use PowerPoint.

We have resubmitted all figures and source data files at a higher resolution to meet the journal’s quality standards and ensure image integrity. All blot source data have been uploaded as TIFF files.

- Thanks for providing the source data. Please upload the source data as one folder per final main figure, grouping together the separate files (in excel format) for the panels, and ZIPed together.

The source data has been reorganized as requested. We have provided one folder per final main figure, each containing the separate files for the individual panels.

In addition, I would need from you uploaded separately:
a short, two-sentence summary of the manuscript (not more than 35 words).

two to four short (!) bullet points highlighting the key findings of your study (two lines each).

- a schematic summary figure as separate file that provides a sketch of the major findings (not a data image) in jpeg or tiff format (with the exact width of 550 pixels and a height of exactly 300 pixels) that can be used as a visual synopsis on our website.

We have provided the following as separate files in the final submission:

- A two-sentence summary (under 35 words).
- Three bullet points highlighting the key findings.
- A schematic summary figure.

Dr. Asma Belbelazi
Cancer Research UK Manchester Institute,
The University of Manchester
Wilmslow Road,
Manchester M20 4BX
United Kingdom

Dear Dr. Belbelazi,

Thank you for the submission of your final revised manuscript to our editorial offices. I now went through this and your final p-b-p-response and consider the remaining editorial requests as adequately addressed.

I am thus very pleased to accept your manuscript for publication in the next available issue of EMBO reports. Thank you for your contribution to our journal.

Your manuscript will be processed for publication by EMBO Press. It will be copy edited and you will receive page proofs prior to publication. Please note that you will be contacted by Springer Nature Author Services to complete licensing and payment information.

You may qualify for financial assistance for your publication charges - either via a Springer Nature fully open access agreement or an EMBO initiative. Check your eligibility: https://link.springer.com/journal/44319/how-to-publish-with-us

Should you be planning a Press Release on your article, please get in contact with embo_production@springernature.com as early as possible in order to coordinate publication and release dates.

If you have any questions, please do not hesitate to contact the Editorial Office. Thank you for your contribution to EMBO Reports.

Yours sincerely,

Achim Breiling
Senior Editor
EMBO Reports

>>> Please note that it is EMBO Reports policy for the transcript of the editorial process (containing referee reports and your response letter) to be published as an online supplement to each paper. If you do NOT want this, you will need to inform the Editorial Office via email immediately. More information is available here: https://link.springer.com/partners/embo-press/editorial-policies#Peer%20review

EMBO Press Author Checklist

Corresponding Author Name: Asma Belbelazi	
Journal Submitted to: EMBO reports	
Manuscript Number: EMBOR-2025-62870V1	

USEFUL LINKS FOR COMPLETING THIS FORM
The EMBO Journal - Author Guidelines
EMBO Reports - Author Guidelines
Molecular Systems Biology - Author Guidelines
EMBO Molecular Medicine - Author Guidelines

Reporting Checklist for Life Science Articles (updated January
This checklist is adapted from Materials Design Analysis Reporting (MDAR) Checklist for Authors. MDAR establishes a minimum set of requirements in transparent reporting in the life sciences (see Statement of Task: 10.31222/osf.io/9sm4x). Please follow the journal's guidelines in preparing your
Please note that a copy of this checklist will be published alongside your article.

Abridged guidelines for figures
1. Data
The data shown in figures should satisfy the following conditions:
- the data were obtained and processed according to the field's best practice and are presented to reflect the results of the experiments in an accurate and unbiased manner.
- ideally, figure panels should include only measurements that are directly comparable to each other and obtained with the same assay.
- plots include clearly labeled error bars for independent experiments and sample sizes. Unless justified, error bars should not be shown for technical
- if n<5, the individual data points from each experiment should be plotted. Any statistical test employed should be justified.
- Source Data should be included to report the data underlying figures according to the guidelines set out in the authorship guidelines on Data

2. Captions
Each figure caption should contain the following information, for each panel where they are relevant:
- a specification of the experimental system investigated (eg cell line, species name).
- the assay(s) and method(s) used to carry out the reported observations and measurements.
- an explicit mention of the biological and chemical entity(ies) that are being measured.
- an explicit mention of the biological and chemical entity(ies) that are altered/varied/perturbed in a controlled manner.
- the exact sample size (n) for each experimental group/condition, given as a number, not a range;
- a description of the sample collection allowing the reader to understand whether the samples represent technical or biological replicates (including how many animals, litters, cultures, etc.).
- a statement of how many times the experiment shown was independently replicated in the laboratory.
- definitions of statistical methods and measures:
  - common tests, such as t-test (please specify whether paired vs. unpaired), simple χ2 tests, Wilcoxon and Mann-Whitney tests, can be unambiguously identified by name only, but more complex techniques should be described in the methods section;
  - are tests one-sided or two-sided?
  - are there adjustments for multiple comparisons?
  - exact statistical test results, e.g., P values = x but not P values < x;
  - definition of 'center values' as median or average;
  - definition of error bars as s.d. or s.e.m.

Please complete ALL of the questions below.
Select "Not Applicable" only when the requested information is not relevant for your study.

Materials

Newly Created Materials	Information included in the manuscript?	In which section is the information available? (Reagents and Tools Table, Materials and Methods, Figures, Data Availability Section)
New materials and reagents need to be available; do any restrictions apply?	Not Applicable	

Antibodies	Information included in the manuscript?	In which section is the information available? (Reagents and Tools Table, Materials and Methods, Figures, Data Availability Section)
For **antibodies** provide the following information:<br>- Commercial antibodies: RRID (if possible) or supplier name, catalogue number and or/clone number<br>- Non-commercial: RRID or citation	Yes	Reagent and Tool table, Method

DNA and RNA sequences	Information included in the manuscript?	In which section is the information available? (Reagents and Tools Table, Materials and Methods, Figures, Data Availability Section)
**Short novel DNA or RNA including primers, probes**: provide the sequences.	Yes	Method

Cell materials	Information included in the manuscript?	In which section is the information available? (Reagents and Tools Table, Materials and Methods, Figures, Data Availability Section)
**Cell lines:** Provide species information, strain. Provide accession number in repository **OR** supplier name, catalog number, clone number, and/**OR** RRID.	Yes	Reagent and Tool table, Method
**Primary cultures:** Provide species, strain, sex of origin, genetic modification status.	Not Applicable	
Report if the cell lines were recently **authenticated** (e.g., by STR profiling) and tested for mycoplasma contamination.	Yes	Method

Experimental animals	Information included in the manuscript?	In which section is the information available? (Reagents and Tools Table, Materials and Methods, Figures, Data Availability Section)
**Laboratory animals or Model organisms:** Provide species, strain, sex, age, genetic modification status. Provide accession number in repository **OR** supplier name, catalog number, clone number, **OR** RRID.	Not Applicable	
**Animal observed in or captured from the field:** Provide species, sex, and age where possible.	Not Applicable	
Please detail **housing and husbandry conditions**.	Not Applicable	

Plants and microbes	Information included in the manuscript?	In which section is the information available? (Reagents and Tools Table, Materials and Methods, Figures, Data Availability Section)
**Plants:** provide species and strain, ecotype and cultivar where relevant, unique accession number if available, and source (including location for collected wild specimens).	Not Applicable	
**Microbes:** provide species and strain, unique accession number if available, and source.	Not Applicable	

Human research participants	Information included in the manuscript?	In which section is the information available? (Reagents and Tools Table, Materials and Methods, Figures, Data Availability Section)
If collected and within the bounds of privacy constraints report on age, sex and gender or ethnicity for all study participants.	Not Applicable	

Core facilities	Information included in the manuscript?	In which section is the information available? (Reagents and Tools Table, Materials and Methods, Figures, Data Availability Section)
If your work benefited from core facilities, was their service mentioned in the acknowledgments section?	Yes	Main Manuscript

Design

Study protocol	Information included in the manuscript?	In which section is the information available? (Reagents and Tools Table, Materials and Methods, Figures, Data Availability Section)
If study protocol has been **pre-registered, provide DOI in the manuscript**. For clinical trials, provide the trial registration number **OR** cite DOI.	Not Applicable	
Report the **clinical trial registration number** (at ClinicalTrials.gov or equivalent), where applicable.	Not Applicable	

Laboratory protocol	Information included in the manuscript?	In which section is the information available? (Reagents and Tools Table, Materials and Methods, Figures, Data Availability Section)
Provide DOI OR other citation details if **external detailed step-by-step protocols** are available.	Yes	Method

Experimental study design and statistics	Information included in the manuscript?	In which section is the information available? (Reagents and Tools Table, Materials and Methods, Figures, Data Availability Section)
Include a statement about **sample size** estimate even if no statistical methods were used.	Yes	Figure legends
Were any steps taken to minimize the effects of subjective bias when allocating animals/samples to treatment (e.g. **randomization procedure**)? If yes, have they been described?	Not Applicable	
Include a statement about **blinding** even if no blinding was done.	Yes	Method
Describe **inclusion/exclusion criteria** if samples or animals were excluded from the analysis. Were the criteria pre-established?<br><br>If sample or data points were omitted from analysis, report if this was due to attrition or intentional exclusion and provide justification.	Not Applicable	
For every figure, are **statistical tests** justified as appropriate? Do the data meet the assumptions of the tests (e.g., normal distribution)? Describe any methods used to assess it. Is there an estimate of variation within each group of data? Is the variance similar between the groups that are being statistically compared?	Yes	Figure legends

Sample definition and in-laboratory replication	Information included in the manuscript?	In which section is the information available? (Reagents and Tools Table, Materials and Methods, Figures, Data Availability Section)
In the figure legends: state number of times the experiment was **replicated** in laboratory.	Yes	Figure legends
In the figure legends: define whether data describe **technical or biological replicates**.	Yes	Figure legends

Ethics

Ethics	Information included in the manuscript?	In which section is the information available? (Reagents and Tools Table, Materials and Methods, Figures, Data Availability Section)
Studies involving **human participants**: State details of **authority granting ethics approval** (IRB or equivalent committee(s), provide reference number for approval.	Not Applicable	
Studies involving **human participants**: Include a statement confirming that **informed consent** was obtained from all subjects and that the experiments conformed to the principles set out in the WMA Declaration of Helsinki and the Department of Health and Human Services Belmont Report.	Not Applicable	
Studies involving **human participants:** For publication of **patient photos**, include a statement confirming that consent to publish was obtained.	Not Applicable	
Studies involving experimental **animals**: State details of **authority granting ethics approval** (IRB or equivalent committee(s), provide reference number for approval. Include a statement of compliance with ethical regulations.	Not Applicable	
Studies involving **specimen and field samples:** State if relevant **permits** obtained, provide details of authority approving study; if none were required, explain why.	Not Applicable	

Dual Use Research of Concern (DURC)	Information included in the manuscript?	In which section is the information available? (Reagents and Tools Table, Materials and Methods, Figures, Data Availability Section)
Could your study fall under dual use research restrictions? Please check biosecurity documents and list of **select agents and toxins** (CDC): https://www.selectagents.gov/sat/list.htm	Not Applicable	
If you used a select agent, is the security level of the lab appropriate and reported in the manuscript?	Not Applicable	
If a study is subject to dual use research of concern regulations, is the name of the **authority granting approval and reference number** for the regulatory approval provided in the manuscript?	Not Applicable	

Reporting

The MDAR framework recommends adoption of discipline-specific guidelines, established and endorsed through community initiatives. Journals have their own policy about requiring specific guidelines and recommendations to complement MDAR.

Adherence to community standards	Information included in the manuscript?	In which section is the information available? (Reagents and Tools Table, Materials and Methods, Figures, Data Availability Section)
State if relevant guidelines or checklists (e.g., **ICMJE, MIBBI, ARRIVE, PRISMA**) have been followed or provided.	Not Applicable	
For **tumor marker prognostic studies,** we recommend that you follow the **REMARK** reporting guidelines (see link list at top right). See author guidelines, under 'Reporting Guidelines'. Please confirm you have followed these guidelines.	Not Applicable	
For **phase II and III randomized controlled trials**, please refer to the **CONSORT** flow diagram (see link list at top right) and submit the CONSORT checklist (see link list at top right) with your submission. See author guidelines, under 'Reporting Guidelines'. Please confirm you have submitted this list.	Not Applicable	

Data Availability

Data availability	Information included in the manuscript?	In which section is the information available? (Reagents and Tools Table, Materials and Methods, Figures, Data Availability Section)
Have **primary datasets** been deposited according to the journal's guidelines (see 'Data Deposition' section) and the respective accession numbers provided in the Data Availability Section?	Not Applicable	
Were **human clinical and genomic datasets** deposited in a public access-controlled repository in accordance to ethical obligations to the patients and to the applicable consent agreement?	Not Applicable	
Are **computational models** that are central and integral to a study available without restrictions in a machine-readable form? Were the relevant accession numbers or links provided?	Not Applicable	
If publicly available data were reused, provide the respective **data citations in the reference list**.	Not Applicable	
